# Genomic Analysis of the Human Gut Microbiome Suggests Novel Enzymes Involved in Quinone Biosynthesis

**DOI:** 10.3389/fmicb.2016.00128

**Published:** 2016-02-09

**Authors:** Dmitry A. Ravcheev, Ines Thiele

**Affiliations:** Luxembourg Centre for Systems Biomedicine, University of LuxembourgEsch-sur-Alzette, Luxembourg

**Keywords:** human gut microbiome, comparative genomics, quinone biosynthesis, non-orthologous displacements, energy production

## Abstract

Ubiquinone and menaquinone are membrane lipid-soluble carriers of electrons that are essential for cellular respiration. Eukaryotic cells can synthesize ubiquinone but not menaquinone, whereas prokaryotes can synthesize both quinones. So far, most of the human gut microbiome (HGM) studies have been based on metagenomic analysis. Here, we applied an analysis of individual HGM genomes to the identification of ubiquinone and menaquinone biosynthetic pathways. In our opinion, the shift from metagenomics to analysis of individual genomes is a pivotal milestone in investigation of bacterial communities, including the HGM. The key results of this study are as follows. (i) The distribution of the canonical pathways in the HGM genomes was consistent with previous reports and with the distribution of the quinone-dependent reductases for electron acceptors. (ii) The comparative genomics analysis identified four alternative forms of the previously known enzymes for quinone biosynthesis. (iii) Genes for the previously unknown part of the futalosine pathway were identified, and the corresponding biochemical reactions were proposed. We discuss the remaining gaps in the menaquinone and ubiquinone pathways in some of the microbes, which indicate the existence of further alternate genes or routes. Together, these findings provide further insight into the biosynthesis of quinones in bacteria and the physiology of the HGM.

## Introduction

**Figure 1:**
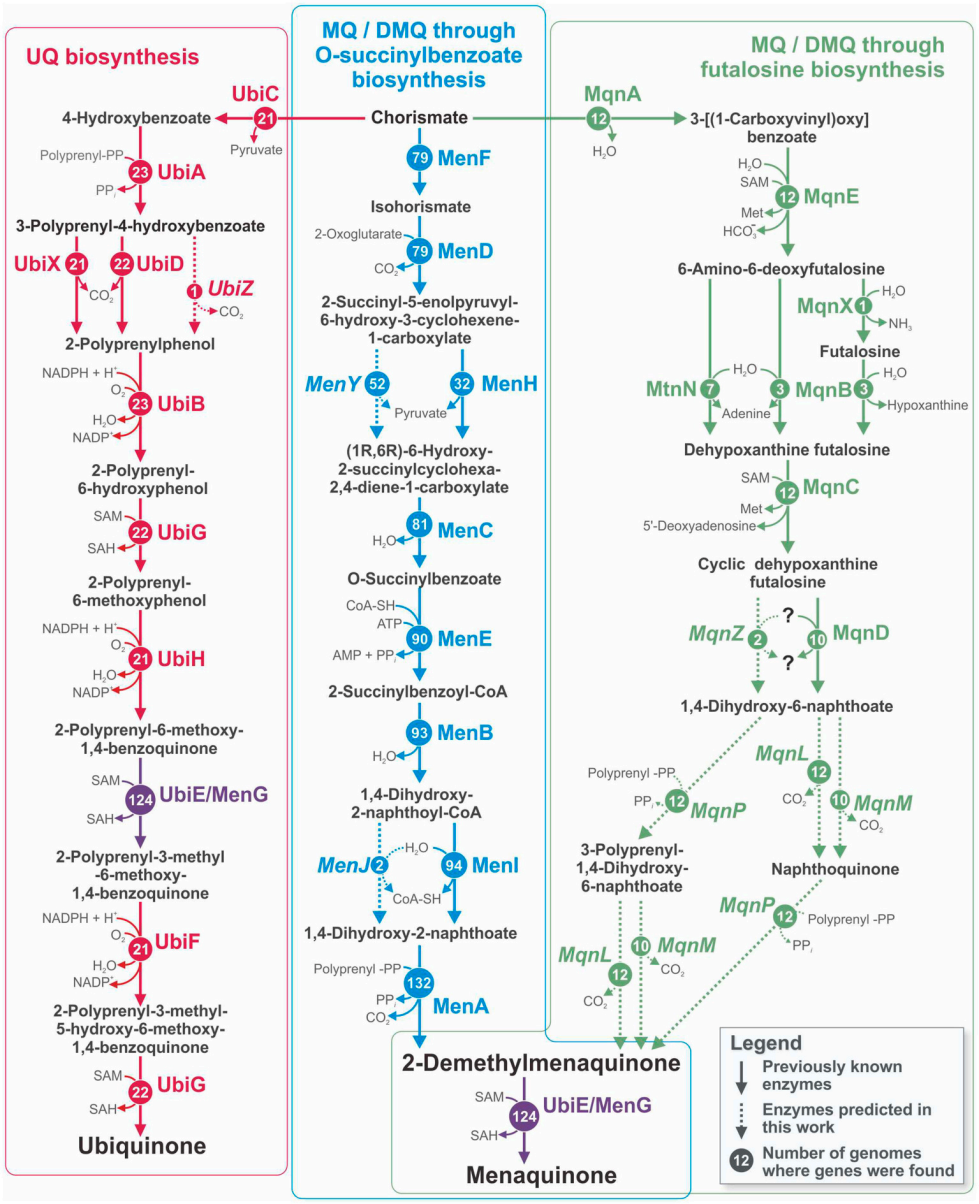
**Pathways for UQ, MK, and DMK biosynthesis in the analyzed genomes**. The names of relevant enzymes are shown for each reaction. Circled numbers show the numbers of genomes in which genes for the corresponding enzyme were found. Solid arrows correspond to previously known enzymes, and dashed arrows together with italicized enzyme names correspond to enzymes predicted in the current work. Because the products of the reactions catalyzed by MenL and MenP are unknown, two possible variants of the pathway are shown. Abbreviations: Met, L-methionine; SAH, S-Adenosyl-L-homocysteine; SAM, S-adenosyl-L-methionine.

Quinones are membrane lipid-soluble carriers of electrons that are essential for cellular respiration (Collins and Jones, 1981). Of the numerous types of quinones used for respiration, the three that are the most studied and most widespread among microorganisms are ubiquinone (UQ, coenzyme Q), menaquinone (MK, vitamin K), and 2-demethylmenaquinone (DMK) (Collins and Jones, 1981; Nowicka and Kruk, 2010). In bacteria, only one pathway has been described for UQ biosynthesis (Meganathan, 2001b), whereas two different pathways are known for MK synthesis (Figure 1). The first “traditional” pathway includes DMK as an immediate precursor of MK, and both MK and DMK are synthesized *via* this pathway (Bentley and Meganathan, 1982; Meganathan, 2001a). In the second pathway, which was more recently discovered, MK is synthesized through futalosine (Hiratsuka et al., 2008). The final steps of this pathway remain unclear, and no information is available about the synthesis of DMK by this pathway (Arakawa et al., 2011; Barta et al., 2014; Zhi et al., 2014). UQ can be synthesized by *Alpha*-, *Beta*-, and *Gammaproteobacteria* and by *Eukaryotes*, whereas MK/DMK can be synthesized only by various groups of *Bacteria* and *Archaea* (Collins and Jones, 1981; Bentley and Meganathan, 1982; Meganathan, 2001a; Nowicka and Kruk, 2010; Zhi et al., 2014). All three pathways begin with chorismate and have no shared enzymes except for the UbiE/MenG methyltransferase, which is involved in both the UQ and the “traditional” MK/DMK pathways (Lee et al., 1997; Figure 1).

Because of the inability of humans to synthesize MK, this compound should be consumed in food. MK is found in meat, dairy, and fermented food products (Walther et al., 2013). MK can also be obtained by the interconversion of a dietary derived phylloquinone (PK). PK is synthetized by plants and *Cyanobacteria* and differs from MK only by its side chain. While MK has a polyprenyl side chain, PK has a phytyl side chain (Nowicka and Kruk, 2010). The extent of PK-derived MK has been estimated to range from 5 to 25% of the digested PK (Shearer and Newman, 2008; Shearer et al., 2012). The dietary requirements of vitamin K (i.e., PK and MK) range from 0.86 to 3.15 μg per day for infants of 0–6 months (Canfield et al., 1990, 1991; Greer et al., 1991; Shearer and Newman, 2008) and from 0.75 to 1.0 μg per kg of body mass per day for adults (Frick et al., 1967; Booth and Al Rajabi, 2008; Shearer et al., 2012).

The classic role of MK in humans is as an enzyme cofactor for the γ-carboxylation of peptide-bound glutamate residues, and evidence has recently been mounting that there is a correlation between human health and dietary MK (Shearer and Newman, 2008, 2014; Booth, 2009; Van Winckel et al., 2009; Walther et al., 2013).

In addition to dietary sources, a portion of the total available MK is synthesized by the human gut microbiome (HGM); however, the extent of MK derived from the HGM has not been determined (Ramotar et al., 1984; Conly and Stein, 1992; Suttie, 1995; LeBlanc et al., 2013; Ramakrishna, 2013). Various HGM communities have been intensively studied in recent years (Eckburg et al., 2005; Gill et al., 2006; Sonnenburg et al., 2006; Kinross et al., 2011; Flint et al., 2012; Lozupone et al., 2012; Leimena et al., 2013; Maurice et al., 2013), but most of these studies have concentrated on the analysis of metagenomic data. Metagenomic analysis is a powerful tool for the determination of HGM composition in healthy and diseased states (Cowan et al., 2005; Kinross et al., 2011; Simon and Daniel, 2011; Cho and Blaser, 2012; Gosalbes et al., 2012; Kelly and Mulder, 2012; Walker et al., 2014) as well as HGM variability related to age (Clemente et al., 2012; Yatsunenko et al., 2012), diet (Kurokawa et al., 2007; Hehemann et al., 2010; Wu et al., 2011), geography (Yatsunenko et al., 2012; Tyakht et al., 2013; Suzuki and Worobey, 2014), host genetics, and lifestyle (Yatsunenko et al., 2012). However, analysis of the completely or partially assembled genomes of representative HGM samples can also yield additional knowledge about the cellular physiology of individual bacterial strains and information about interactions between different organisms. Thus, a shift from metagenomic analysis to the analysis of individual genome sequences may become important in the investigation of the HGM and its interactions with the human organism. To date, there are 382 HGM genomes available through the National Institutes of Health (NIH) Human Microbiome Project (http://www.hmpdacc.org/HMRGD/), providing an excellent opportunity for gaining a further understanding of HGM biology.

## Materials and methods

From the list of human gut microbes found in at least 50% of the analyzed HGMs (Nelson et al., 2010; Qin et al., 2010), we selected 250 genomes of human intestinal inhabitants that were available in the PubSEED (Overbeek et al., 2005; Disz et al., 2010) and Integrated Microbial Genomes (IMG) databases (Markowitz et al., 2014). The following four model genomes were added to the reference set: an inhabitant of the lower gut, *Escherichia coli* K-12 MG1655 (Blattner et al., 1997); an intestinal inflammation-causing agent, *Salmonella enterica* Typhimurium LT2 (Winter et al., 2010); a model organism for the analysis of carbohydrate metabolism in the intestine, *Bacteroides thetaiotaomicron* VPI-5482 (Hooper et al., 2002; Xu et al., 2003); and a model organism related to multiple gut strains, *Bacillus subtilis* 168 (Hong et al., 2009). All 254 of the selected genomes are presented in Table S1 in Supplementary Materials. The added model organisms belong to the three main phyla represented in HGM (Nelson et al., 2010; Qin et al., 2010), Bacteroidetes (*B. thetaiotaomicron*), Firmicutes (*B. subtilis*), and Proteobacteria (*E. coli* and *S. enterica*). We included of the model organisms to expand known metabolic functions in these organisms to the HGM genomes as well as to use their predictions as mean to quality control the predictions for the HGM genomes. For example, usage of genomes for the model organisms can be used to avoid wrong predictions for the functions of novel genes by verifying their absence in the model genomes. Note that three of the four model organisms (*B. thetaiotaomicron, E. coli*, and *S. enterica*) have been also detected in HGM, but in less than 50% of the studied samples (Qin et al., 2010).

The PubSEED platform was used to annotate the genes for quinone biosynthesis proteins. To avoid misannotations, all of the proteins with the same function were checked for orthology. Orthologs were defined as the best bidirectional hits that have a similar genomic context. To search for the best bidirectional hits, a BLAST algorithm (Altschul et al., 1997) implemented in PubSEED and the IMG platform was used (cutoff = e^−20^). Additionally, in the search for orthologs, the GenomeExplorer program package (Mironov et al., 2000) was used, and orthologs were determined as the best bidirectional hits with protein identity no less than 20%. To analyze genomic context and gene occurrence, we used PubSEED and STRING v9.1 (Franceschini et al., 2013) along with phylogenetic trees for protein domains in MicrobesOnline (Dehal et al., 2010). To analyze protein domain structure, we used searches in the Pfam (Finn et al., 2014) and CDD (Marchler-Bauer et al., 2013) databases, and the “Domains and Families” service implemented in the MicrobesOnline platform. Additionally, functional annotations of the analyzed genes were performed using the UniProt (Magrane and Consortium, 2011) and KEGG (Kanehisa et al., 2012) databases.

Multiple protein alignments were performed using the ClustalX v 2.0 tool (protein weight matrix: BLOSUM series; gap opening: 15; gap extension: 0.5) (Larkin et al., 2007). Phylogenetic trees were constructed using the maximum-likelihood method with the default parameters implemented in PhyML-3.0 (Guindon et al., 2010). The obtained trees were visualized and midpoint-rooted using the interactive viewer Dendroscope, version 3.2.10, build 19 (Huson et al., 2007). To clarify the taxonomic affiliations of the analyzed genomes, the NCBI Taxonomy database (http://www.ncbi.nlm.nih.gov/taxonomy) was used. To predict substrate specificities according to the specificity-determining positions (SDP), the SDPfox web tool (Mazin et al., 2010) was used with the maximum percent of gaps allowed in a group in each column being 50%. As an input for the SDP analysis, we used multiple alignments for all UbiA/MqnP, UbiD/MqnL, and UbiX/MqnM proteins found in the analyzed genomes. No preliminary division to into specific groups was done. The specificity groups were determined based on the SDP analysis as follows. An uploaded aligned sequence set was randomly divided into groups (the minimal number of the groups = 2, the number of the sequences = 10), then “SPDgroup” procedure and the “Move sequences according to the best weight” procedure were applied.

All of the annotated genes are represented as a subsystem in PubSEED (http://pubseed.theseed.org/SubsysEditor.cgi?page=ShowSubsystem&subsystem=Quinones_biosynthesis_HGM) and all of the protein sequences for the annotated genes in FASTA format are represented in file Sequences S1 in the Supplementary Materials.

## Results

Here, we present a systematic analysis of the biosynthetic pathways for UQ, MK, and DMK in 254 genomes, including 250 genomes for commonly found in the human gut (Qin et al., 2010) and four genomes for model organisms. Application of a comparative genomic analysis to individual HGM genomes identified the distribution of various pathways for quinone biosynthesis in HGM microorganisms and revealed four alternative forms of known enzymes in these pathways. Our analysis resulted in a prediction of the genes responsible for the last three steps of the futalosine biosynthesis pathway, which were previously unknown. Additionally, we compared the distribution of quinone biosynthetic pathways with the distribution of quinone-dependent reductases for electron acceptors within the HGM genomes.

### Known genes for quinone biosynthesis

To reconstruct the biosynthetic pathways of UQ, MK, and DMK in the 254 studied genomes, we analyzed the distribution of previously known genes involved in these pathways (Figure 1). On the basis of the presence of known genes, a pathway can be classified as complete or incomplete. A pathway was assigned as complete when known genes found in the genome could form an uninterrupted biosynthetic pathway from chorismate to UQ or MK and as incomplete when the pathway was interrupted because of the absence of certain genes. The presence of only one gene was classified as an incomplete pathway. To avoid misannotations, all genes possibly involved in quinone biosynthesis were checked for orthology with the known genes, as described in “Experimental procedures.” The distribution of the genes for UQ biosynthesis (Ubi pathway) among the analyzed genomes was restricted to *Gamma*- and *Betaproteobacteria*. Complete Ubi pathways were found in 19 genomes (Table S1 in Supplementary Materials), whereas incomplete pathways were found in 4 genomes. The pathway for MK/DMK biosynthesis through O-succinylbenzoate (Men pathway) was more broadly distributed among the analyzed genomes. Complete Men pathways were found in 24 genomes of *Firmicutes* and *Proteobacteria*, whereas incomplete pathways were found in 91 genomes belonging to the phyla *Actinobacteria, Bacteroidetes, Firmicutes, Fusobacteria, Proteobacteria*, and *Verrucomicrobia*. Because the enzymes for the last steps of MK synthesis through the futalosine biosynthesis pathway (Mqn pathway) were not known (Arakawa et al., 2011; Barta et al., 2014; Zhi et al., 2014), this pathway was considered complete when all of the enzymes catalyzing the reactions for the synthesis of 1,4-dihydroxy-6-naphthoate from chorismate (Figure 1) were found in the genome. The complete pathway was found in 10 genomes from the phyla *Bacteroidetes, Firmicutes*, and *Proteobacteria*, whereas an incomplete pathway was found in 2 *Firmicutes* genomes.

### Novel enzyme in the Ubi pathway

**Figure 2:**
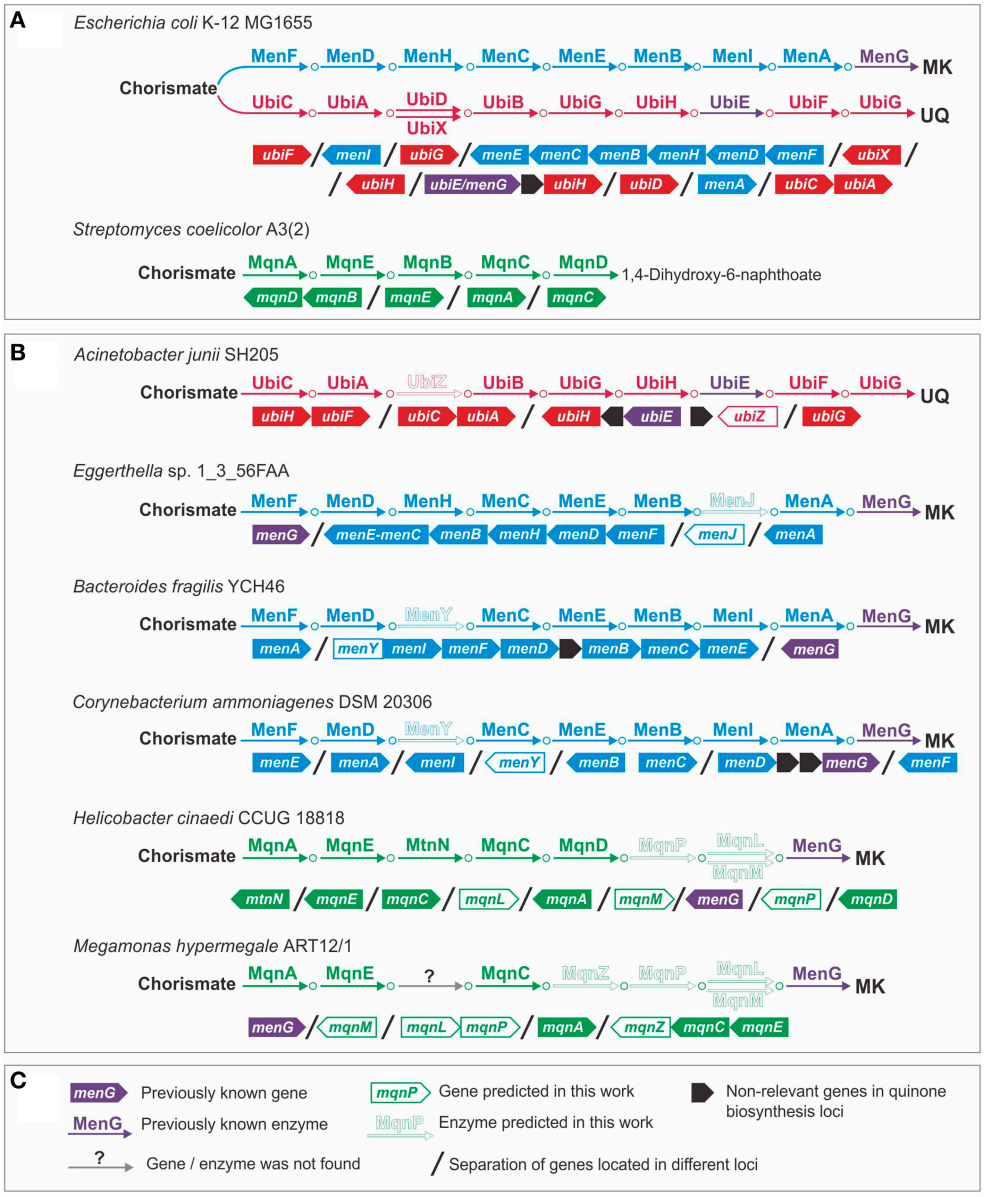
**Non-orthologous replacements and novel genes in quinone biosynthesis pathways. (A)** Model organisms; **(B)** examples of genomes with predicted non-orthologous replacements and novel genes; **(C)** legend.

Using genomic analysis of the Ubi pathway, we predicted an alternative form of 3-polyprenyl-4-hydroxybenzoate carboxy-lyase (EC 4.1.1.-) (Figure 2). Previously, two alternative forms of this enzyme, UbiD and UbiX, were described (Cox et al., 1969; Alexander and Young, 1978; Gulmezian et al., 2007). Analysis of the genome of *Acinetobacter junii* SH205 revealed the presence of all the genes for the Ubi pathway except for *ubiD* and *ubiX*. To find a non-orthologous replacement (Koonin et al., 1996) for this enzyme, we searched for *ubiD* and *ubiX* orthologs in the genomes of organisms related to *A. junii* (Figure 2). These genomes belonged to two families of the order *Pseudomonadales*: *Moraxellaceae* (which includes *A. junii*) and *Pseudomonadaceae*. Orthologs of *Escherichia coli* K-12 MG1655 *ubiD* (*e* ≤ 9e^−54^, identity ≥ 86%) and *ubiX* (*e* ≤ 3e^−60^, identity ≥ 54%) were found in *Pseudomonadaceae* but not in the *Moraxellaceae* genomes. Thus, the *Moraxellaceae* genomes should encode an alternative 3-polyprenyl-4-hydroxybenzoate carboxy-lyase that is non-orthologous to *ubiD* or *ubiX*, and this alternative gene should be present in *Moraxellaceae* but not in *Pseudomonadaceae*. Analysis of gene occurrence by IMG revealed 29 candidates. A hypothetical protein (the locus tag in *A. junii* is HMPREF0026_02430), was considered to be the most probable candidate because of its co-localization on the chromosome with the *ubiE* and *ubiB* genes in the genomes of *Acinetobacter* spp. and *Psychrobacter* sp. PRwf-1. We named this gene *ubiZ*. The number of genomes with a complete Ubi pathway thus increased from 19 to 20.

### Novel enzymes in the men pathway

We predicted alternative genes for 1,4-dihydroxy-2-naphthoyl-CoA thioesterase (EC 3.1.2.28) and for (1R,6R)-2-succinyl-6-hydroxy-2,4-cyclohexadiene-1-carboxylate synthase (EC 4.2.99.20) (Figure 2). The first enzyme is typically encoded by the *menI* gene (Widhalm et al., 2009; Chen et al., 2013), whereas the second enzyme is typically encoded by the *menH* gene (Jiang et al., 2007, 2008). No *menI* orthologs were found in the genomes of *Eggerthella* sp. 1_3_56FAA or *Gordonibacter pamelaeae* 7-10-1-b. A sequence similarity search using the tblastn algorithm (Altschul et al., 1997) revealed in these genomes a distantly related homolog of *menI* (*e* = 1.4, identity = 30%, the locus tag in *G. pamelaeae* is GPA_07290) that was annotated as a hypothetical protein. Because such an *e*-value and identity is not significant, we verified the presence of this protein in the analyzed genomes. The orthologs of this new gene were found only in genomes having the Men pathway but lacking *menI*. Consequently, we propose that this gene, called *menJ*, could replace the function of *menI*. There are no additional corroborations for this prediction, such as a genomic location with other MK synthesis genes or a sequence similarity to functionally relevant proteins. Hence, this prediction remains quite speculative.

A non-orthologous replacement for the *menH* gene was predicted by a detailed analysis of the *menI* genes in the studied genomes. This analysis revealed two types of MenI proteins. The first type has the same domain structure as a protein from *E. coli* and includes a single thioesterase domain (Pfam ID: PF03061). The second type of MenI has the same PF03061 domain at its C-terminus and an additional hydrolase domain (Pfam ID: PF08282) at its N-terminus (Figure S1A in Supplementary Materials). The PF08292 domain belongs to the haloacid dehydrogenase superfamily, the members of which catalyze reactions similar to the one catalyzed by MenH (Koonin and Tatusov, 1994). In the analyzed genomes, we observed two types of orthologs containing the PF08282 domain: the PF08282-MenI fusions and proteins having a single PF08282 domain (Figure S1B in Supplementary Materials). All the proteins that contained a PF08282 domain were present only in genomes lacking the MenH orthologs. Thus, the PF08282-domain protein, called MenY, was proposed to be a non-orthologous displacement for MenH. The prediction of the *menJ* and *menY* genes increased the number of genomes with a complete Men pathway from 24 to 72.

### Novel enzyme in the Mqn pathway

Using a genomic analysis of the Mqn pathway, we predicted an alternative form of 1,4-dihydroxy-6-naphthoate synthase (EC 1.14.-.-) and proposed an enzyme for the previously unknown steps of this pathway. In the genomes of *Mitsuokella multacida* DSM 20544 and *Megamonas hypermegale* ART12/1, no *mqnD* genes were found, but we detected a gene that co-localized together with the *mqnC* and *mqnE* genes (the locus tag in *M. hypermegale* is MHY_27640). In addition to *M. multacida* and *M. hypermegale*, this protein was found to cluster with the *mqnC* and *mqnE* genes in six genomes, including *Selenomonas artemidis* F0399, *Selenomonas flueggei* ATCC 43531, *Selenomonas noxia* ATCC 43541, *Selenomonas* sp. 67H29BP, *Selenomonas* sp. F0430, and *Selenomonas sputigena* ATCC 35185. In further four genomes, *Pelotomaculum thermopropionicum* SI, *Syntrophomonas wolfei* str. Goettingen, *Syntrophothermus lipocalidus* DSM 12680, and *Thermosinus carboxydivorans*, this protein was found to co-localize with the *mqnEAC* operon. Because this gene was found only in conjunction with the other genes in this pathway but not with *mqnD*, we proposed that this gene (*mqnZ*) can replace the gene function of *mqnD*. Unfortunately, sequence analysis of this protein did not provide additional support for its prediction as the only sequence similarity was found with the phosphorylase superfamily (Pfam ID: PF01048) (*e* = 1.14e^−32^).

### Prediction of the last steps of the Mqn pathway

Previously, it has been proposed that the final biosynthetic stages of the Mqn pathway should be catalyzed by a polyprenyltransferase, a carboxy-lyase, and a methyltransferase (Arakawa et al., 2011; Barta et al., 2014; Zhi et al., 2014), but the particular enzymes are unknown. Analysis of genomes containing genes for the Mqn pathway revealed genes that are homologous to *ubiE/menG, ubiA, ubiD*, and *ubiX*. The UbiE/MenG proteins encode S-adenosyl-L-methionine-dependent methyltransferases that transfer a methyl group to the carbon atom of the aromatic ring, which is part of both UQ and MQ. These methyltransferases are involved in both the Ubi and Men pathways (Lee et al., 1997). Thus, we propose that the Mqn co-occurring homologs of UbiE/MenG could be involved in menaquinone synthesis. To test this hypothesis, we constructed a phylogenetic tree for the UbiE/MenG-like proteins co-occurring with the Ubi, Men, and Mqn pathways (Figure S2 in Supplementary Materials). The Mqn co-occurring proteins did not form a separate monophyletic branch but were mixed with the Men co-occurring proteins. Based on this co-occurrence, we propose that the Mqn co-occurring UbiE/MenG proteins play the same role as UbiE/MenG, i.e., catalyzing the methylation of DMK to produce MK.

The UbiA protein catalyzes the attachment of a polyprenyl group to the quinone aromatic ring (Melzer and Heide, 1994). We propose that the Mqn co-occurring homologs of UbiA are involved in the Mqn pathway. On the other hand, in the Mqn pathway a polyprenyl group could be attached to 1,4-dihydroxy-6-naphthoate or its derivatives, whereas UbiA catalyzes the attachment of a polyprenyl group to 4-hydroxybenzoate. Thus, different substrate specificities of UbiA and its Mqn co-occurring homologs could correlate with distinguishable differences on the amino acid sequence level. Correlations between phylogeny and substrate specificity have been reported for various enzymes (Lee et al., 2007; Olivares-Hernández et al., 2010; Reddy et al., 2013; Ratnikov et al., 2014). Consequently, we proposed that UbiA and its Mqn co-occurring homolog form a clearly distinguished branches in their phylogenetical tree. To test this hypothesis, we constructed a phylogenetic tree (Figure S3 in Supplementary Materials) for the three homolog groups: (1) UbiA proteins, (2) the Mqn co-occurring homologs of UbiA, and (3) MenA proteins. Each group in the tree formed a monophyletic branch. The UbiA proteins and the Mqn co-occurring homologs of UbiA (named MqnP) branches were neighboring but were clearly separated from each other. Thus, we propose that UbiA and MqnP have similar functions but have different substrate specificities. The MqnP protein is proposed to be a polyprenyltransferase in the Mqn pathway.

The UbiD and UbiX proteins are carboxy-lyases in the Ubi pathway (Cox et al., 1969; Alexander and Young, 1978; Gulmezian et al., 2007). Homologs for both of these proteins were found in the genomes encoding enzymes for the Mqn pathway. As for the UbiA homologs, we suspected that the Ubi pathway proteins and their Mqn co-occurring homologs would have different substrate specificities and would be clearly separated based on the corresponding phylogenetic trees. Construction of such trees (Figure S4A in Supplementary Materials) confirmed this proposition. The UbiD proteins and their Mqn co-occurring homologs formed monophyletic branches that were clearly separated from each other. The same was true for the UbiX proteins and their Mqn co-occurring homologs (Figure S4B in Supplementary Materials). Based on these results, we propose that the Mqn co-occurring homologs of UbiD and UbiX are carboxy-lyases in the Mqn pathway; we named them MqnL and MqnM, respectively.

Additionally, the presence of SDPs (Kalinina et al., 2004; Mazin et al., 2010) was analyzed for the UbiA/MqnP, UbiD/MqnL, and UbiX/MqnM proteins were found in the HGM genomes. For each of these groups of homologous, 33, 27, and 7 SDPs were determined, respectively (Figure S5 in Supplementary Materials). After grouping by determined SDPs (see “Materials and Methods” for details), each set of homologs was divided to two groups. In all three groups of homologs, SDPs-based groups turned out to be in agreement with the division of phylogenetic trees and our predictions of functions.

Thus, the enzymes for all three steps (polyprenylation, decarboxylation, and methylation) in the transformation of 1,4-dihydroxy-6-naphthoate into MK were predicted. However, the order of these steps is not completely clear. Because the Mqn pathway associated with UbiE/MenG was indistinguishable from the Men pathway, we propose that methylation may be the final step of the Mqn pathway, as is the case for the Men pathway. For the ordering of polyprenylation and decarboxylation, we propose two alternative scenarios (Figure 1). In the first scenario, 1,4-dihydroxy-6-naphthoate is initially polyprenylated to form 3-polyprenyl-1,4-dihydroxy-6-naphthoate, which then is decarboxylated to DMK. In the second scenario, 1,4-dihydroxy-6-naphthoate is initially decarboxylated to form naphthoquinone, which could then be polyprenylated to form DMK. The *mqnP, mqnL*, and *mqnM* genes were all found in each genome containing the Mqn pathway, but these genes were absent in the other genomes, supporting our predictions of their involvement in the Mqn pathway.

### Co-distribution of quinone biosynthetic pathways and quinone-dependent reductases for electron acceptors

Based on the presence of the Ubi, Men, and Mqn pathways in each analyzed genome, we predicted the patterns of the synthesized quinones. The ability to synthesize UQ is directly determined by the presence of the Ubi pathway, whereas the situation for DMK and MK synthesis is more complicated. Because DMK is an intermediate in the Men and Mqn pathways, organisms with genomes having one of these pathways should be able to synthesize DMK. The ability to synthesize MK depends on the presence of the *ubiE/menG* genes. In the presence of the Men or Mqn pathway, having *ubiE/menG* in the genome determines the ability to synthesize both MK and DMK. Meanwhile, the presence of the Men or Mqn pathway without *ubiE/menG* in the genome results in the synthesis of DMK only. Overall, five different patterns of quinone synthesis were found (Table S2 in Supplementary Materials). A total of 19 genomes were able to synthesize UQ, MK, and DMK, 4 genomes were able to synthesize UQ only, 99 genomes were able to synthesize MK and DMK, 8 genomes were able to synthesize DMK only, and, finally, 124 genomes were not able to synthesize any of the quinones.

The distribution of quinone synthesis patterns in the HGM genomes was also compared to the distribution patterns of quinone-dependent reductases for electron acceptors (also referred to as reductases) obtained from a previous analysis of HGM genomes (Ravcheev and Thiele, 2014). Only quinone-interacting reductases were included in our analysis. A reductase was considered as quinone-interacting if orthologs of known quinone-interacting subunits were found in chromosomal gene cluster encoding for this reductase. In total, we analyzed 17 types of reductases with the following electron acceptors: oxygen (Cyo, Qox, and Cyd), nitrate (Nar and Nap), nitrite (Nrf), tetrathionate (Ttr), thiosulfate (Phs and Tsr), sulfite (Dsr), polysulfite (Psr), trimethylamine N-oxide (Tor), dimethyl sulfoxide (Dms), selenate (Ynf), fumarate (Frd), and arsenate (Arx), as well as the Ynf reductase with unknown specificity. Overall, orthologs of 23 quinone-interacting membrane subunits were found in the analyzed genomes. For 14 of these membrane subunits, interactions with quinones have been confirmed experimentally, whereas for the 6 proteins interactions with quinones have been previously predicted based on similarity with known quinone-interacting subunits. For the remaining three proteins, TsrF, DmsH, and YdhD, interactions with quinones were predicted in this study based on sequence similarity with the experimentally validated quinone-interacting NrfD protein from *E. coli* (Table S3 in Supplementary Materials).

**Figure 3:**
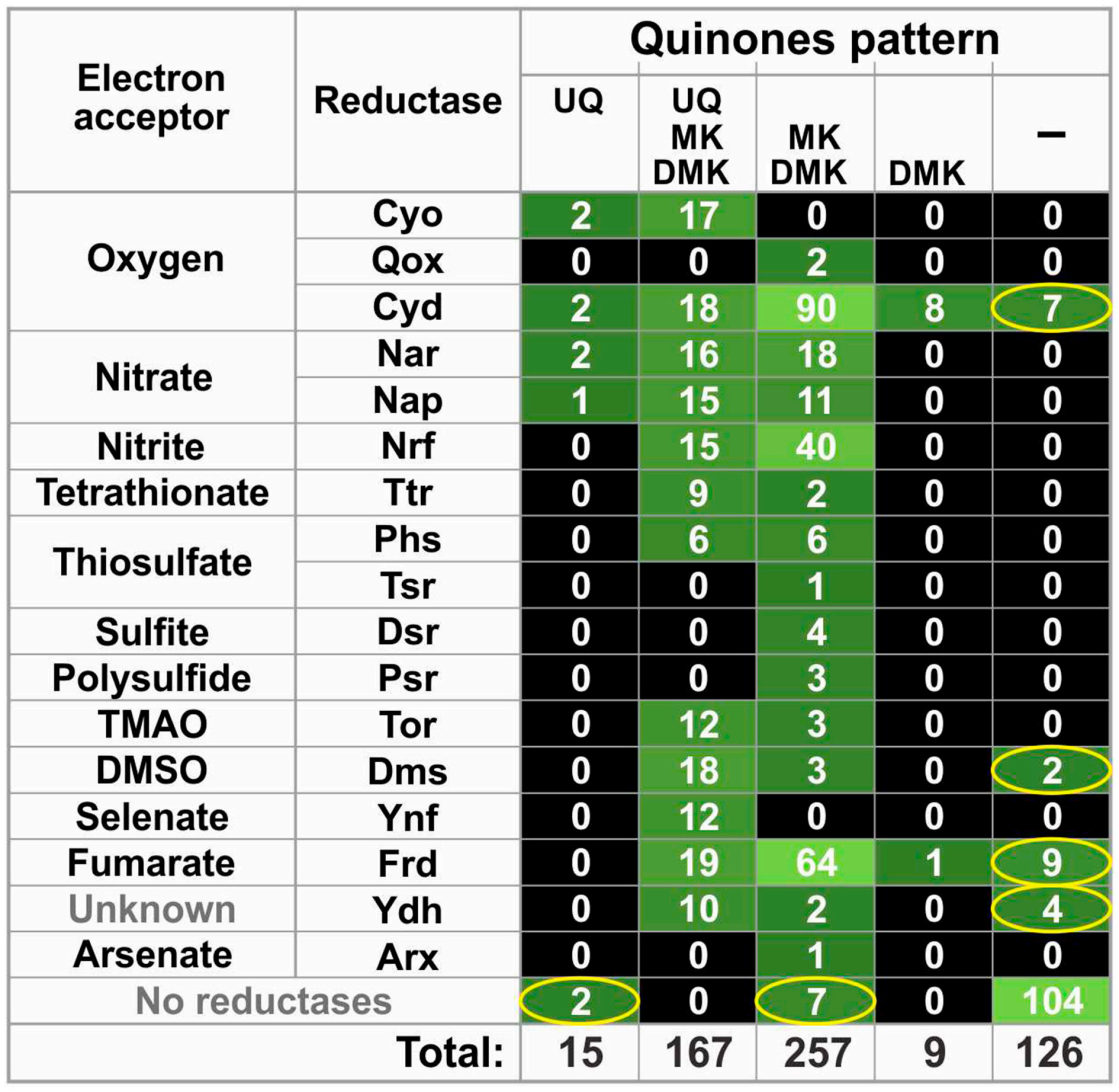
**Co-distribution of quinone biosynthetic pathways and quinone-dependent reductases for electron acceptors**. The number of genomes is shown. Inconsistencies between the reductase and quinone patterns are indicated by ellipses. TMAO, trimethylamine N-oxide; DMSO, dimethyl sulfoxide.

Of the 254 genomes, 225 (88.6%) genomes demonstrated good agreement between the distributions of reductases and quinones (Figure 3 and Table S2 in Supplementary Materials): 121 genomes (47.6%) encoded both reductases and quinone-synthesis pathways, whereas the remaining 104 genomes (40.9%) encoded neither reductases nor quinone-synthesis pathways. The remaining 29 genomes (11.4%) disagreed in their distributions of quinone-synthesis pathways and reductases. In 20 genomes (7.9%), reductases were identified but no quinone biosynthetic pathways were found. Finally, 9 genomes (3.5%) encoded pathways able to synthesize at least one quinone but contained no identified reductases.

## Discussion

In this study, we analyzed the distribution of three pathways for the biosynthesis of respiratory quinones in 254 genomes, including 250 genomes for microbes commonly found in the healthy human gut and four genomes for model organisms. Our key results are as follows. (i) The HGM distribution of canonical pathways was consistent with previous reports and with the distribution of reductases for electron acceptors. (ii) A comparative genomics analysis identified four alternative forms of the previously known enzymes for quinone biosynthesis. (iii) Genes for a previously unknown part of the futalosine pathway were identified, and the corresponding biochemical reactions, enzymes, and genes were proposed. Furthermore, we discuss the remaining gaps in some of the genomes.

The distributions of the three studied quinone biosynthesis pathways in the HGM correspond to previous data on the distribution of these pathways (Collins and Jones, 1981; Nowicka and Kruk, 2010). For instance, the Men pathway has been previously shown to be more frequent among *Prokaryotes* (Zhi et al., 2014). This observation corresponds to the presence of this pathway in almost half of the analyzed genomes. In comparison, the Ubi and Mqn pathways are present in 9 and 5% of the studied genomes, respectively. Additionally, only *Alpha*-, *Beta*-, and *Gammaproteobacteria* synthesize UQ (Meganathan, 2001b; Cluis et al., 2012). Indeed, among the studied genomes, the UQ biosynthetic genes were not found to be absent in any of analyzed *Beta*- or *Gammaproteobacteria* (no *Alphaproteobacteria* genomes were analyzed in this work). Two-thirds of the studied genomes belong to genera for which experimental data on the production of respiratory quinones is available (Table S4 in Supplementary Materials). The predictions from the genomic analysis were consistent with the experimental data for all HGM genomes. A similar concordance was observed when the distributions of quinone biosynthetic pathways and of quinone-dependent reductases for electron acceptors were compared (Figure 3). For instance, the genes for the UQ-interacting aerobic reductase complex CyoABCD (Abramson et al., 2000) were found only in genomes encoding the UQ biosynthetic pathway. Additionally, anaerobic reductases for tetrathionate, thiosulfate, polysulfide, sulfite, fumarate, trimethylamine N-oxide, dimethyl sulfoxide, selenate, and arsenate were found exclusively in the genomes encoding the MK/DMK biosynthetic pathways, but not in genomes having only the UQ or DMK biosynthetic pathways. A comparative genomics approach can thus be used to accurately annotate quinone biosynthetic pathways.

Comparative genomic analysis of the quinone biosynthetic pathways revealed four non-orthologous replacements for previously known enzymes. A total of four alternative enzymes were predicted: one for the Ubi pathway, two for the Men Pathway, and one for the Mqn pathway. These predictions were made possible by analysis of multiple genome sequences and by the use of multiple comparative genomics methods. For example, the prediction of MenY, a non-orthologous replacement for the previously known protein MenH, is based on the following assumptions. (1) The MenY protein belongs to the superfamily of HAD hydrolases, i.e., its general function is relevant. (2) The MenY protein forms protein fusions (multi-domain proteins) with the MenI protein. (3) The *menY* gene is co-located on the chromosome with other *men* genes in the 31 studied genomes. (4) The *menY* gene is present only in genomes that lack the *menH* gene. Each of these assumptions alone is not enough to confirm the prediction that MenY is a MenH-replacing enzyme, but collectively, they provide a sufficient basis for such a prediction. Thus, the combinatorial use of multiple methods can increase the impact of genome-based predictions. Additionally, the use of comparative genomics for the prediction of alternative enzyme forms provides a good basis for further experimental validation.

The most notable result of this work is the prediction of previously unknown stages of the futalosine pathway and of genes encoding the corresponding enzymes: *mqnP, mqnL*, and *mqnM* (Figure 1). This prediction was permitted by the application of multiple genomic techniques to large numbers of analyzed genomes. Further experimental verification is required to confirm the validity of this prediction. This re-annotation also resolves the incomplete Ubi pathway in a number of genomes outside of *Alpha*-, *Beta*-, and *Gammaproteobacteria*, making the annotation consistent with data on the taxonomic distribution of this pathway (Meganathan, 2001b; Cluis et al., 2012).

The detection of enzymes for previously unknown stages of the Mqn pathway increases our knowledge of the evolutionary history of the quinone biosynthetic pathways. The Mqn pathway was shown to be the primordial pathway for the MK biosynthesis, whereas the Men pathway appeared later in evolution (Zhi et al., 2014). The narrow taxonomic distribution of the Ubi pathway clearly indicates that it is the most recently evolved pathway. The results of the current work specify the details of pathway evolution. Quinone biosynthetic pathways that newly emerge during evolution may use parts of preexisting pathways. Thus, the Men pathway adopted a methyltransferase and, because the MenA is a homolog of the MqnP, possibly a polyprenyltransferase from the more ancient Mqn pathway. The Ubi pathway, the youngest of the three studied pathways, adopted three proteins from the Mqn pathway: polyprenyltransferase and two non-homologous carboxy-lyases. Additionally, the Ubi pathway adopted a methyltransferase from the Mqn or Men pathway. Thus, younger pathways contain more enzymes adopted from older pathways. This assumption is based on only the three analyzed pathways and is therefore quite speculative. Nonetheless, this assumption can lead to an interesting conclusion. For example, the alternative isoprenoid quinones, such as sulfolobusquinone, caldariellaquinone, and benzodithiophenoquinone, have a very narrow taxonomic distribution (Nowicka and Kruk, 2010; Zhi et al., 2014), and their biosynthetic pathways should be younger than the Men and Mqn pathways. The pathway for the biosynthesis of the alternative quinones would thus be expected to contain homologs of enzymes from more ancient pathways. This expectation could be used to predict such pathways. Of course, such predictions will also be quite speculative and will require experimental validation. However, if this assumption is true, it can be used for the prediction of various novel pathways beyond quinone biosynthesis.

## Further directions

Our study resulted in the functional predictions for a number of genes involved in quinone biosynthesis. Such predictions illustrate the power of comparative analysis of individual genomes. Nonetheless, some problems related to microbial quinone biosynthesis still remain unresolved. The first problem is the presence of incomplete biosynthesis pathways. For instance, incomplete Men pathways were found in 43 genomes. Three non-exclusive hypotheses might explain this pathway incompleteness: (1) the incompleteness of genome sequences, (2) inter-microbe exchange of metabolites, and (3) non-orthologous replacements. Two-thirds of the analyzed genomes have a draft status, and some genes for the quinone biosynthetic pathways may thus be absent from the current version of the genome. Completion of the sequences of current draft genomes may complete the Men pathway. For example, the draft genome sequence of *Escherichia* sp. 1_1_43 lacks genes for the Men pathway and lacks some genes in the Ubi pathway (Table S1 in Supplementary Materials). Nevertheless, in all the complete genomes of *Escherichia* spp., all the genes for these two pathways were found. Thus, we can be confident that the quinone biosynthesis genes missing from the current genome of *Escherichia* sp. 1_1_43 will be detected in the finished version of this genome. Thus, the first further direction is an update of the study results using a novel, complete version of previously incomplete genomes.

**Figure 4:**
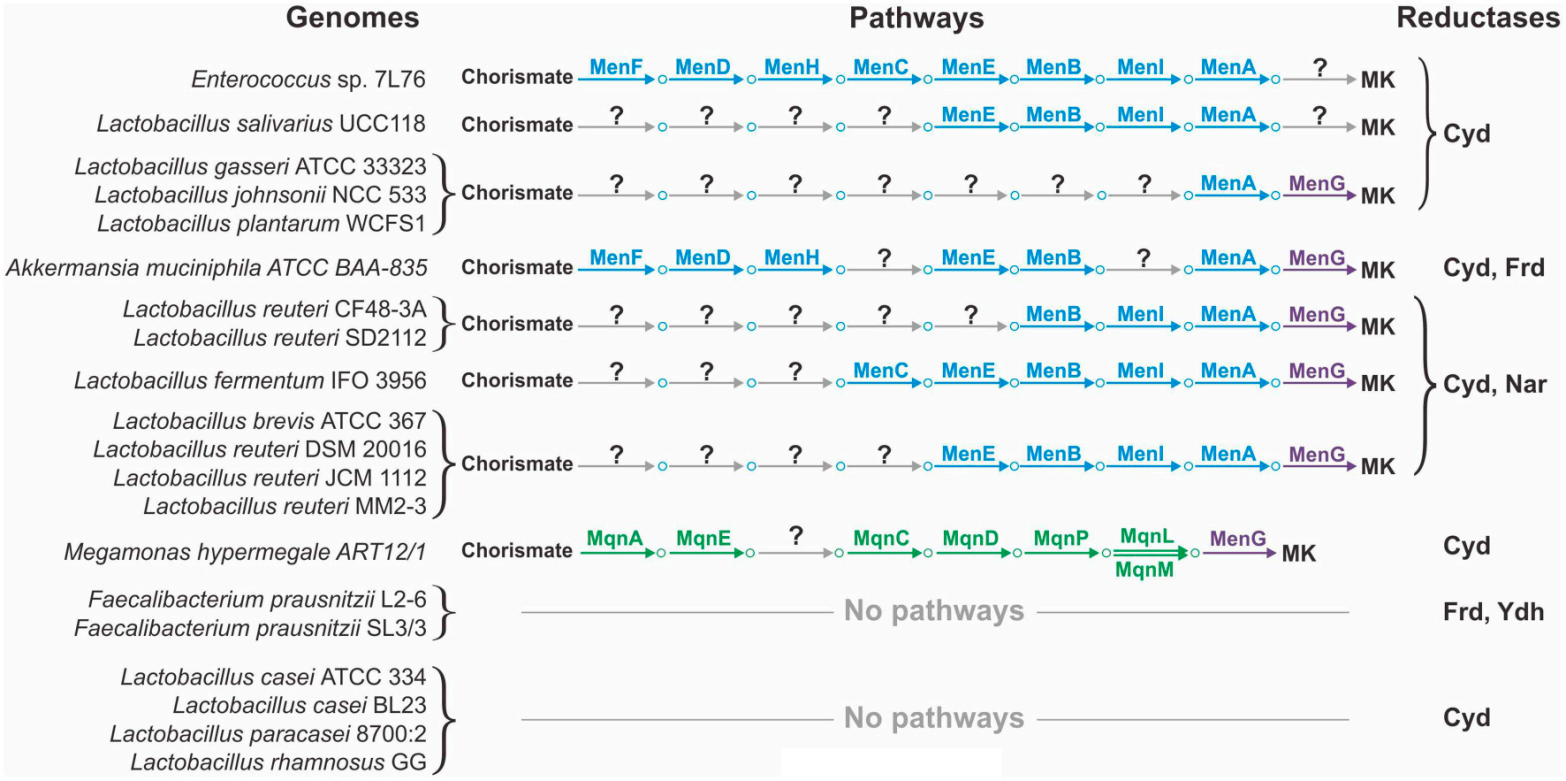
**Incompleteness or loss of quinone biosynthetic pathways in the finished genomes**.

The availability of finished genomes for all the studied organisms will only partially resolve the problem of pathway incompleteness, as incomplete pathways were also found in a number of finished genomes (Figure 4). For example, the multiple finished genomes of *Lactobacillus* spp. showed incomplete Men pathways. The common feature of these incomplete pathways is that early steps of the pathway are missing whereas late steps are present, at least the steps required for the polyprenylation and methylation catalyzed by MenA and MenG, respectively (Figure 4). Because the addition of a hydrophobic polyprenyl group takes place at the penultimate step of the Men pathway, all MK precursors from isochorismate to 1,4-dihydroxy-2-naphthoate are soluble; thus, exchange of these metabolites between different microorganisms could be possible. For example, 1,4-dihydroxy-2-naphthoate could be used by microbes having only the *menAG* genes, whereas O-succinylbenzoate could be used by microbes having the *menEBIAG* genes. We hypothesize that such organisms could utilize soluble precursors of MK. Hence, the corresponding transport genes should be present in their genomes, but remain to be annotated. Two types of transporters would be required, (1) transporters for the export of soluble quinone precursors in the producing organisms and (2) transporters for the import of these precursors in the consuming organisms. Whereas nothing is known about the exchange of quinone precursors in microbial communities, inter-species exchange of metabolites has been demonstrated for the HGM. For instance, HGM organisms can exchange acetate, extracellular polysaccharides, formate, fucose, molecular hydrogen, secondary bile acids, short-chain fatty acids, sialic acid, and succinate (Stams and Plugge, 2009; De Vuyst and Leroy, 2011; Ng et al., 2013; Kovács, 2014; Vogt et al., 2015). Thus, the proposed exchange of soluble quinone precursors is very likely.

The hypothesis that MK precursors may be exchanged between microbes is tempting but remains speculative. To confirm this hypothesis, the transporters of the corresponding MK precursors must be computationally predicted and then experimentally validated. For better support of the exchange hypothesis, co-presence of the producing and consuming organisms in various HGM samples would need to be analyzed. These inter-microbe interactions should also be validated experimentally and/or tested computationally using the mathematical models (see below).

Even if the exchange hypothesis were true, the problem of incomplete pathways could not be sufficiently resolved. In some finished genomes, such as *Akkermansia muciniphila* or *Megamonas hypermegale*, the pathways lack internal steps (Figure 4). For these genomes, an important next step would be the search for non-orthologous replacements in incomplete pathways. Non-orthologous replacements are well known for quinone biosynthetic pathways. For example, in *E. coli*, the decarboxylation of 3-polyprenyl-4-hydroxybenzoate can be catalyzed by two non-homologous proteins, UbiD and UbiX (Cox et al., 1969; Alexander and Young, 1978; Gulmezian et al., 2007). Another example is the existence of multiple non-orthologous replacements for enzymes catalyzing the early steps of the Mqn pathway (Arakawa et al., 2011). Additionally, four non-orthologous replacements were predicted in this study. The non-orthologous replacement hypothesis is very promising because these replacements can be successfully determined even using computational methods alone. If such replacements are found, the problem of pathway incompleteness could be resolved.

The other unresolved problem is the inconsistency between the distributions of quinone biosynthetic pathways and quinone-dependent reductases for electron acceptors (Figure 3). Two main types of inconsistencies were observed: (1) absence of reductases in the presence of quinone biosynthetic pathways and (2) absence of quinone biosynthetic pathways in the presence of reductases. Of course, both of these problems may be partially resolved by the availability of finished genomes. On the other hand, such inconsistencies were also found in finished genomes (Table S1 in Supplementary Materials). The absence of reductases could be explained by the use of unknown reductases by an organism. In a previous study, we have predicted two novel membrane reductases among HGM genomes (Ravcheev and Thiele, 2014), and at least one of them, thiosulfate reductase Tsr, was predicted as quinone-dependent (Table S3 in Supplementary Materials). Further systematic analysis of respiratory enzymes in the HGM genomes could resolve this type of inconsistencies.

The absence of quinone biosynthetic pathways could be explained by the existence of alternative quinone biosynthetic pathways. For instance, the alternative Mqn pathway for MK biosynthesis has been discovered in 2008 (Hiratsuka et al., 2008). Additionally, alternative types of quinones may be used for respiration in organisms having reductases but lacking the Ubi, Men, and Mqn pathways. In this study, we limited our analysis to the biosynthesis of UQ, MK, and DMK, but the diversity of microbial respiratory quinones is much wider (Collins and Jones, 1981; Nowicka and Kruk, 2010). Nonetheless, the biosynthetic pathways of alternative quinones are poorly understood. We anticipate that increasing wealth of experimental and genomic data will substantially improve our understanding of these pathways.

In this study, the non-orthologous displacements and genes for previously unknown reactions were only computationally predicted; thus requiring experimental confirmation. For example, the predicted 2-succinyl-5-enolpyruvyl-6-hydroxy-3-cyclohexene-1-carboxylate dehydrogenase MenY could be verified using model organisms, such as *Corynebacterium* spp. or *Bacteroides* spp. Similarly, the novel enzymes in the Mqn pathway could be experimentally analyzed using *Helicobacter* spp.

Furthermore, the respiratory systems in the HGM organisms are not limited to reductases and quinones. For broader coverage of the respiratory systems, our study will need to be extended to the analysis of ATP synthases and dehydrogenases for electron donors. Other pathways of relevance to human health, such as the B-vitamin synthesis capability of the HGM (Magnúsdóttir et al., 2015), should be also considered. The identification of novel enzymes and pathways will lead to better understanding of the HGM biochemistry and physiology. This is a pre-requisite to understand the HGM's contribution to human health and disease as well as to rationally alter HGM. Current approaches aiming to modify the HGM, such as fecal transplants (Kelly et al., 2015) and probiotics, lack mechanistic bases, which is partially due to insufficient biochemical knowledge.

The quinone biosynthesis needs also to be analyzed in the context human-microbe interactions. An inability of human cells to synthetize MK, together with the importance of this compound for the human health (Shearer and Newman, 2008, 2014; Booth, 2009; Van Winckel et al., 2009; Walther et al., 2013), raises the question about a role of gut microbiota in the MK supply of the host. So far, MK biosynthesis has been studied in monocultures of model organisms (Bentley and Meganathan, 1982; Ramotar et al., 1984; Fernandez and Collins, 1987; Walther et al., 2013) or in animal-microbe models (Kindberg et al., 1987; Davidson et al., 1998) but the information about the human-microbe exchange of MK is still very scarce. Nonetheless, rat models demonstrated that luminal concentrations of MK produced by *Escherichia coli* and *Bacteroides vulgatus* could reach 6–7 and 8 μg per g of dry feces, respectively. Assuming a similar ratio for humans and a daily fecal output of 25-50 g solid matter in healthy individuals (Wyman et al., 1978), microbial produced and excreted MK could range from 150 to 400 μg feces. The recommended dietary intake of vitamin K, of which MK is a minor part, is for sucking infants 0.86–3.15 μg per day for sucking infants (Canfield et al., 1990, 1991; Greer et al., 1991; Shearer and Newman, 2008) and for adults 75–90 μg per day (Frick et al., 1967; Booth and Al Rajabi, 2008; Shearer et al., 2012). In fact, the microbial contribution to MK requirements has been suggested to be approximately 50% (Wyman et al., 1978), but evidence is still missing.

Computational modeling (Palsson, 2006; Orth et al., 2010) of HGM metabolism could be used to systematically elucidate the MK biosynthesis potential of different HGM representatives. In fact, genome-scale metabolic models for numerous HGM microorganisms have been published (Thiele et al., 2005, 2011, 2012; Orth et al., 2011; Branco Dos Santos et al., 2013; Heinken et al., 2013, 2014; Bauer et al., 2015), but still require better coverage of the respiratory chain and quinone biosynthesis pathways. At the same time, computational models for human metabolism (Duarte et al., 2007; Sahoo et al., 2012, 2015; Heinken et al., 2013; Thiele et al., 2013a,b) are available, thereby, enabling the *in silico* study of HGM and their interactions with each other (Freilich et al., 2011; Zomorrodi and Maranas, 2012; Khandelwal et al., 2013; Heinken and Thiele, 2015a) as well as with the human host (Thiele et al., 2013a; Bauer et al., 2015; Heinken and Thiele, 2015b,c). Particularly, such a modeling approach could help us to estimate the microbial contribution to the MK requirements in humans.

Quinone biosynthesis has been studied in many taxonomically diverse microbial species (Collins and Jones, 1981; Meganathan, 2001b; Shearer and Newman, 2008; Nowicka and Kruk, 2010; Cluis et al., 2012), but no systematic analysis of quinone biosynthesis has yet been done for microbes found in a particular ecosystem. Considering that many microbes remain unculturable, as their culturing conditions remain unidentified, it is crucial to search their genomes for potential exchanges of metabolites with other community members. Particularly, quinones may play an important role in the co-metabolism of microbial communities, as they are main electron transfer molecules in microbial respiratory chains. Quinones influence the energy production and further, through cellular redox status and central metabolism, they affect the utilization of carbon and nitrogen sources and biosynthesis of indispensable compounds, such as amino and fatty acids. Thus, the ability of a microbe to *de novo* synthetize, salvage, or utilize quinones determines vital cellular properties, such as growth and replication. The presented results of the distribution of quinone biosynthetic pathways could be further expanded to include strains with high relevance in the biotechnological industry, in ecology, and in human health. Such large-scale comparative genomics effort could provide further insight into evolutionary mechanisms, including co-evolution of microbiomes with the host.

## Author contributions

DR and IT conceived and designed the research project and wrote the manuscript. DR performed genomic analysis of the quinone biosynthetic pathway. All authors read and approved the final manuscript.

### Conflict of interest statement

The authors declare that the research was conducted in the absence of any commercial or financial relationships that could be construed as a potential conflict of interest.

## References

[B1] Abramson J. Riistama S. Larsson G. Jasaitis A. Svensson-Ek M. Laakkonen L.. (2000). The structure of the ubiquinol oxidase from *Escherichia coli* and its ubiquinone binding site. Nat. Struct. Biol. 7, 910–917. 10.1038/82824 11017202

[B2] Alexander K. Young I. G. (1978). Alternative hydroxylases for the aerobic and anaerobic biosynthesis of ubiquinone in *Escherichia coli*. Biochemistry 17, 4750–4755. 10.1021/bi00615a024 365223

[B3] Altschul S. F. Madden T. L. Schäffer A. A. Zhang J. Zhang Z. Miller W.. (1997). Gapped BLAST and PSI-BLAST: a new generation of protein database search programs. Nucleic Acids Res. 25, 3389–3402. 10.1093/nar/25.17.3389 9254694 PMC146917

[B4] Arakawa C. Kuratsu M. Furihata K. Hiratsuka T. Itoh N. Seto H.. (2011). Diversity of the early step of the futalosine pathway. Antimicrob. Agents Chemother. 55, 913–916. 10.1128/AAC.01362-10 21098241 PMC3028782

[B5] Barta M. L. Thomas K. Yuan H. Lovell S. Battaile K. P. Schramm V. L.. (2014). Structural and biochemical characterization of *Chlamydia trachomatis* hypothetical protein CT263 supports that menaquinone synthesis occurs through the futalosine pathway. J. Biol. Chem. 289, 32214–32229. 10.1074/jbc.M114.594325 25253688 PMC4231696

[B6] Bauer E. Laczny C. C. Magnusdottir S. Wilmes P. Thiele I. (2015). Phenotypic differentiation of gastrointestinal microbes is reflected in their encoded metabolic repertoires. Microbiome 3, 55. 10.1186/s40168-015-0121-6 26617277 PMC4663747

[B7] Bentley R. Meganathan R. (1982). Biosynthesis of vitamin K (menaquinone) in bacteria. Microbiol. Rev. 46, 241–280. 6127606 10.1128/mr.46.3.241-280.1982 PMC281544

[B8] Blattner F. R. Plunkett G. Bloch C. A. Perna N. T. Burland V. Riley M.. (1997). The complete genome sequence of *Escherichia coli* K-12. Science 277, 1453–1474. 10.1126/science.277.5331.1453 9278503

[B9] Booth S. L. (2009). Roles for vitamin K beyond coagulation. Annu. Rev. Nutr. 29, 89–110. 10.1146/annurev-nutr-080508-141217 19400704

[B10] Booth S. L. Al Rajabi A. (2008). Determinants of vitamin K status in humans. Vitam. Horm. 78, 1–22. 10.1016/S0083-6729(07)00001-5 18374187

[B11] Branco Dos Santos F. De Vos W. M. Teusink B. (2013). Towards metagenome-scale models for industrial applications–the case of Lactic Acid Bacteria. Curr. Opin. Biotechnol. 24, 200–206. 10.1016/j.copbio.2012.11.003 23200025

[B12] Canfield L. M. Hopkinson J. M. Lima A. F. Martin G. S. Sugimoto K. Burr J.. (1990). Quantitation of vitamin K in human milk. Lipids 25, 406–411. 10.1007/BF02537985 2395420

[B13] Canfield L. M. Hopkinson J. M. Lima A. F. Silva B. Garza C. (1991). Vitamin K in colostrum and mature human milk over the lactation period–a cross-sectional study. Am. J. Clin. Nutr. 53, 730–735. 2000828 10.1093/ajcn/53.3.730

[B14] Chen M. Ma X. Chen X. Jiang M. Song H. Guo Z. (2013). Identification of a hotdog fold thioesterase involved in the biosynthesis of menaquinone in *Escherichia coli*. J. Bacteriol. 195, 2768–2775. 10.1128/JB.00141-13 23564174 PMC3697248

[B15] Cho I. Blaser M. J. (2012). The human microbiome: at the interface of health and disease. Nat. Rev. Genet 13, 260–270. 10.1038/nrg3182 22411464 PMC3418802

[B16] Clemente J. C. Ursell L. K. Parfrey L. W. Knight R. (2012). The impact of the gut microbiota on human health: an integrative view. Cell 148, 1258–1270. 10.1016/j.cell.2012.01.035 22424233 PMC5050011

[B17] Cluis C. P. Pinel D. Martin V. J. (2012). The production of coenzyme Q10 in microorganisms. Subcell. Biochem. 64, 303–326. 10.1007/978-94-007-5055-5_15 23080257

[B18] Collins M. D. Jones D. (1981). Distribution of isoprenoid quinone structural types in bacteria and their taxonomic implication. Microbiol. Rev. 45, 316–354. 7022156 10.1128/mr.45.2.316-354.1981 PMC281511

[B19] Conly J. M. Stein K. (1992). Quantitative and qualitative measurements of K vitamins in human intestinal contents. Am. J. Gastroenterol. 87, 311–316. 1539565

[B20] Cowan D. Meyer Q. Stafford W. Muyanga S. Cameron R. Wittwer P. (2005). Metagenomic gene discovery: past, present and future. Trends Biotechnol. 23, 321–329. 10.1016/j.tibtech.2005.04.001 15922085

[B21] Cox G. B. Young I. G. McCann L. M. Gibson F. (1969). Biosynthesis of ubiquinone in *Escherichia coli* K-12: location of genes affecting the metabolism of 3-octaprenyl-4-hydroxybenzoic acid and 2-octaprenylphenol. J. Bacteriol. 99, 450–458. 4897112 10.1128/jb.99.2.450-458.1969 PMC250037

[B22] Davidson R. T. Foley A. L. Engelke J. A. Suttie J. W. (1998). Conversion of dietary phylloquinone to tissue menaquinone-4 in rats is not dependent on gut bacteria. J. Nutr. 128, 220–223. 9446847 10.1093/jn/128.2.220

[B23] Dehal P. S. Joachimiak M. P. Price M. N. Bates J. T. Baumohl J. K. Chivian D.. (2010). MicrobesOnline: an integrated portal for comparative and functional genomics. Nucleic Acids Res. 38, D396–D400. 10.1093/nar/gkp919 19906701 PMC2808868

[B24] De Vuyst L. Leroy F. (2011). Cross-feeding between bifidobacteria and butyrate-producing colon bacteria explains bifdobacterial competitiveness, butyrate production, and gas production. Int. J. Food Microbiol. 149, 73–80. 10.1016/j.ijfoodmicro.2011.03.003 21450362

[B25] Disz T. Akhter S. Cuevas D. Olson R. Overbeek R. Vonstein V.. (2010). Accessing the SEED genome databases via Web services API: tools for programmers. BMC Bioinformatics 11:319. 10.1186/1471-2105-11-319 20546611 PMC2900279

[B26] Duarte N. C. Becker S. A. Jamshidi N. Thiele I. Mo M. L. Vo T. D.. (2007). Global reconstruction of the human metabolic network based on genomic and bibliomic data. Proc. Natl. Acad. Sci. U.S.A. 104, 1777–1782. 10.1073/pnas.0610772104 17267599 PMC1794290

[B27] Eckburg P. B. Bik E. M. Bernstein C. N. Purdom E. Dethlefsen L. Sargent M.. (2005). Diversity of the human intestinal microbial flora. Science 308, 1635–1638. 10.1126/science.1110591 15831718 PMC1395357

[B28] Fernandez F. Collins M. D. (1987). Vitamin K composition of anaerobic gut bacteria. FEMS Microbiol. Lett. 41, 175–180. 10.1111/j.1574-6968.1987.tb02191.x

[B29] Finn R. D. Bateman A. Clements J. Coggill P. Eberhardt R. Y. Eddy S. R.. (2014). Pfam: the protein families database. Nucleic Acids Res. 42, D222–D230. 10.1093/nar/gkt1223 24288371 PMC3965110

[B30] Flint H. J. Scott K. P. Duncan S. H. Louis P. Forano E. (2012). Microbial degradation of complex carbohydrates in the gut. Gut Microbes 3, 289–306. 10.4161/gmic.19897 22572875 PMC3463488

[B31] Franceschini A. Szklarczyk D. Frankild S. Kuhn M. Simonovic M. Roth A.. (2013). STRING v9.1: protein-protein interaction networks, with increased coverage and integration. Nucleic Acids Res. 41, D808–D815. 10.1093/nar/gks1094 23203871 PMC3531103

[B32] Freilich S. Zarecki R. Eilam O. Segal E. S. Henry C. S. Kupiec M.. (2011). Competitive and cooperative metabolic interactions in bacterial communities. Nat. Commun. 2, 589. 10.1038/ncomms1597 22158444

[B33] Frick P. G. Riedler G. Brögli H. (1967). Dose response and minimal daily requirement for vitamin K in man. J. Appl. Physiol. 23, 387–389. 6047959 10.1152/jappl.1967.23.3.387

[B34] Gill S. R. Pop M. Deboy R. T. Eckburg P. B. Turnbaugh P. J. Samuel B. S.. (2006). Metagenomic analysis of the human distal gut microbiome. Science 312, 1355–1359. 10.1126/science.1124234 16741115 PMC3027896

[B35] Gosalbes M. J. Abellan J. J. Durbán A. Pérez-Cobas A. E. Latorre A. Moya A. (2012). Metagenomics of human microbiome: beyond 16s rDNA. Clin. Microbiol. Infect. 18(Suppl. 4), 47–49. 10.1111/j.1469-0691.2012.03865.x 22647049

[B36] Greer F. R. Marshall S. Cherry J. Suttie J. W. (1991). Vitamin K status of lactating mothers, human milk, and breast-feeding infants. Pediatrics 88, 751–756. 1896278

[B37] Guindon S. Dufayard J. F. Lefort V. Anisimova M. Hordijk W. Gascuel O. (2010). New algorithms and methods to estimate maximum-likelihood phylogenies: assessing the performance of PhyML 3.0. Syst. Biol. 59, 307–321. 10.1093/sysbio/syq010 20525638

[B38] Gulmezian M. Hyman K. R. Marbois B. N. Clarke C. F. Javor G. T. (2007). The role of UbiX in *Escherichia coli* coenzyme Q biosynthesis. Arch. Biochem. Biophys. 467, 144–153. 10.1016/j.abb.2007.08.009 17889824 PMC2475804

[B39] Hehemann J. H. Correc G. Barbeyron T. Helbert W. Czjzek M. Michel G. (2010). Transfer of carbohydrate-active enzymes from marine bacteria to Japanese gut microbiota. Nature 464, 908–912. 10.1038/nature08937 20376150

[B40] Heinken A. Khan M. T. Paglia G. Rodionov D. A. Harmsen H. J. M. Thiele I. (2014). A functional metabolic map of Faecalibacterium prausnitzii, a beneficial human gut microbe. J. Bacteriol. 196, 3289–3302. 10.1128/JB.01780-14 25002542 PMC4135701

[B41] Heinken A. Sahoo S. Fleming R. M. Thiele I. (2013). Systems-level characterization of a host-microbe metabolic symbiosis in the mammalian gut. Gut Microbes 4, 28–40. 10.4161/gmic.22370 23022739 PMC3555882

[B42] Heinken A. Thiele I. (2015a). Anoxic conditions promote species-specific mutualism between gut microbes *in silico*. Appl. Environ. Microbiol 81, 4049–4061. 10.1128/AEM.00101-15 25841013 PMC4524141

[B43] Heinken A. Thiele I. (2015b). Systematic prediction of health-relevant human-microbial co-metabolism through a computational framework. Gut Microbes 6, 120–130. 10.1080/19490976.2015.1023494 25901891 PMC4615372

[B44] Heinken A. Thiele I. (2015c). Systems biology of host-microbe metabolomics. Wiley Interdiscip. Rev. Syst. Biol. Med. 7, 195–219. 10.1002/wsbm.1301 25929487 PMC5029777

[B45] Hiratsuka T. Furihata K. Ishikawa J. Yamashita H. Itoh N. Seto H.. (2008). An alternative menaquinone biosynthetic pathway operating in microorganisms. Science 321, 1670–1673. 10.1126/science.1160446 18801996

[B46] Hong H. A. Khaneja R. Tam N. M. Cazzato A. Tan S. Urdaci M.. (2009). *Bacillus subtilis* isolated from the human gastrointestinal tract. Res. Microbiol. 160, 134–143. 10.1016/j.resmic.2008.11.002 19068230

[B47] Hooper L. V. Midtvedt T. Gordon J. I. (2002). How host-microbial interactions shape the nutrient environment of the mammalian intestine. Annu. Rev. Nutr. 22, 283–307. 10.1146/annurev.nutr.22.011602.092259 12055347

[B48] Huson D. H. Richter D. C. Rausch C. Dezulian T. Franz M. Rupp R. (2007). Dendroscope: an interactive viewer for large phylogenetic trees. BMC Bioinformatics 8:460. 10.1186/1471-2105-8-460 18034891 PMC2216043

[B49] Jiang M. Cao Y. Guo Z. F. Chen M. Chen X. Guo Z. (2007). Menaquinone biosynthesis in *Escherichia coli*: identification of 2-succinyl-5-enolpyruvyl-6-hydroxy-3-cyclohexene-1-carboxylate as a novel intermediate and re-evaluation of MenD activity. Biochemistry 46, 10979–10989. 10.1021/bi700810x 17760421

[B50] Jiang M. Chen X. Guo Z. F. Cao Y. Chen M. Guo Z. (2008). Identification and characterization of (1R,6R)-2-succinyl-6-hydroxy-2,4-cyclohexadiene-1-carboxylate synthase in the menaquinone biosynthesis of *Escherichia coli*. Biochemistry 47, 3426–3434. 10.1021/bi7023755 18284213

[B51] Kalinina O. V. Mironov A. A. Gelfand M. S. Rakhmaninova A. B. (2004). Automated selection of positions determining functional specificity of proteins by comparative analysis of orthologous groups in protein families. Protein Sci. 13, 443–456. 10.1110/ps.03191704 14739328 PMC2286703

[B52] Kanehisa M. Goto S. Sato Y. Furumichi M. Tanabe M. (2012). KEGG for integration and interpretation of large-scale molecular data sets. Nucleic Acids Res. 40, D109–D114. 10.1093/nar/gkr988 22080510 PMC3245020

[B53] Kelly C. R. Kahn S. Kashyap P. Laine L. Rubin D. Atreja A.. (2015). Update on fecal microbiota transplantation 2015: indications, methodologies, mechanisms, and outlook. Gastroenterology 149, 223–237. 10.1053/j.gastro.2015.05.008 25982290 PMC4755303

[B54] Kelly D. Mulder I. E. (2012). Microbiome and immunological interactions. Nutr. Rev. 70(Suppl. 1), S18–S30. 10.1111/j.1753-4887.2012.00498.x 22861803

[B55] Khandelwal R. A. Olivier B. G. Röling W. F. Teusink B. Bruggeman F. J. (2013). Community flux balance analysis for microbial consortia at balanced growth. PLoS ONE 8:e64567. 10.1371/journal.pone.0064567 23741341 PMC3669319

[B56] Kindberg C. Suttie J. W. Uchida K. Hirauchi K. Nakao H. (1987). Menaquinone production and utilization in germ-free rats after inoculation with specific organisms. J. Nutr. 117, 1032–1035. 3298580 10.1093/jn/117.6.1032

[B57] Kinross J. M. Darzi A. W. Nicholson J. K. (2011). Gut microbiome-host interactions in health and disease. Genome Med. 3, 14. 10.1186/gm228 21392406 PMC3092099

[B58] Koonin E. V. Mushegian A. R. Bork P. (1996). Non-orthologous gene displacement. Trends Genet. 12, 334–336. 10.1016/0168-9525(96)20010-1 8855656

[B59] Koonin E. V. Tatusov R. L. (1994). Computer analysis of bacterial haloacid dehalogenases defines a large superfamily of hydrolases with diverse specificity. Application of an iterative approach to database search. J. Mol. Biol. 244, 125–132. 10.1006/jmbi.1994.1711 7966317

[B60] Kovács A. T. (2014). Impact of spatial distribution on the development of mutualism in microbes. Front. Microbiol. 5:649. 10.3389/fmicb.2014.00649 25505463 PMC4241817

[B61] Kurokawa K. Itoh T. Kuwahara T. Oshima K. Toh H. Toyoda A.. (2007). Comparative metagenomics revealed commonly enriched gene sets in human gut microbiomes. DNA Res. 14, 169–181. 10.1093/dnares/dsm018 17916580 PMC2533590

[B62] Larkin M. A. Blackshields G. Brown N. P. Chenna R. McGettigan P. A. McWilliam H.. (2007). Clustal W and Clustal X version 2.0. Bioinformatics 23, 2947–2948. 10.1093/bioinformatics/btm404 17846036

[B63] LeBlanc J. G. Milani C. De Giori G. S. Sesma F. Van Sinderen D. Ventura M. (2013). Bacteria as vitamin suppliers to their host: a gut microbiota perspective. Curr. Opin. Biotechnol. 24, 160–168. 10.1016/j.copbio.2012.08.005 22940212

[B64] Lee J. Michael A. J. Martynowski D. Goldsmith E. J. Phillips M. A. (2007). Phylogenetic diversity and the structural basis of substrate specificity in the beta/alpha-barrel fold basic amino acid decarboxylases. J. Biol. Chem. 282, 27115–27125. 10.1074/jbc.M704066200 17626020

[B65] Lee P. T. Hsu A. Y. Ha H. T. Clarke C. F. (1997). A C-methyltransferase involved in both ubiquinone and menaquinone biosynthesis: isolation and identification of the *Escherichia coli ubiE* gene. J. Bacteriol. 179, 1748–1754. 9045837 10.1128/jb.179.5.1748-1754.1997 PMC178890

[B66] Leimena M. M. Ramiro-Garcia J. Davids M. Van den Bogert B. Smidt H. Smid E. J.. (2013). A comprehensive metatranscriptome analysis pipeline and its validation using human small intestine microbiota datasets. BMC Genomics 14:530. 10.1186/1471-2164-14-530 23915218 PMC3750648

[B67] Lozupone C. A. Stombaugh J. I. Gordon J. I. Jansson J. K. Knight R. (2012). Diversity, stability and resilience of the human gut microbiota. Nature 489, 220–230. 10.1038/nature11550 22972295 PMC3577372

[B68] Magnúsdóttir S. Ravcheev D. A. De Crécy-Lagard V. Thiele I. (2015). Systematic genome assessment of B-vitamin biosynthesis suggests co-operation among gut microbes. Front. Genet. 6:148. 10.3389/fgene.2015.00148 25941533 PMC4403557

[B69] Magrane M. Consortium U. (2011). UniProt Knowledgebase: a hub of integrated protein data. Database 2011:bar009. 10.1093/database/bar009 21447597 PMC3070428

[B70] Marchler-Bauer A. Zheng C. Chitsaz F. Derbyshire M. K. Geer L. Y. Geer R. C.. (2013). CDD: conserved domains and protein three-dimensional structure. Nucleic Acids Res. 41, D348–D352. 10.1093/nar/gks1243 23197659 PMC3531192

[B71] Markowitz V. M. Chen I. M. Palaniappan K. Chu K. Szeto E. Pillay M.. (2014). IMG 4 version of the integrated microbial genomes comparative analysis system. Nucleic Acids Res. 42, D560–D567. 10.1093/nar/gkt963 24165883 PMC3965111

[B72] Maurice C. F. Haiser H. J. Turnbaugh P. J. (2013). Xenobiotics shape the physiology and gene expression of the active human gut microbiome. Cell 152, 39–50. 10.1016/j.cell.2012.10.052 23332745 PMC3552296

[B73] Mazin P. V. Gelfand M. S. Mironov A. A. Rakhmaninova A. B. Rubinov A. R. Russell R. B.. (2010). An automated stochastic approach to the identification of the protein specificity determinants and functional subfamilies. Algorithms Mol. Biol. 5:29. 10.1186/1748-7188-5-29 20633297 PMC2914642

[B74] Meganathan R. (2001a). Biosynthesis of menaquinone (vitamin K2) and ubiquinone (coenzyme Q): a perspective on enzymatic mechanisms. Vitam. Horm. 61, 173–218. 10.1016/S0083-6729(01)61006-9 11153266

[B75] Meganathan R. (2001b). Ubiquinone biosynthesis in microorganisms. FEMS Microbiol. Lett. 203, 131–139. 10.1111/j.1574-6968.2001.tb10831.x 11583838

[B76] Melzer M. Heide L. (1994). Characterization of polyprenyldiphosphate: 4-hydroxybenzoate polyprenyltransferase from *Escherichia coli*. Biochim. Biophys. Acta 1212, 93–102. 10.1016/0005-2760(94)90193-7 8155731

[B77] Mironov A. A. Vinokurova N. P. Gel'fand M. S. (2000). Software for analyzing bacterial genomes. Mol. Biol. 34, 253–262. 10.1007/BF02759643 10779952

[B78] Nelson K. E. Weinstock G. M. Highlander S. K. Worley K. C. Creasy H. H. Wortman J. R.. (2010). A catalog of reference genomes from the human microbiome. Science 328, 994–999. 10.1126/science.1183605 20489017 PMC2940224

[B79] Ng K. M. Ferreyra J. A. Higginbottom S. K. Lynch J. B. Kashyap P. C. Gopinath S.. (2013). Microbiota-liberated host sugars facilitate post-antibiotic expansion of enteric pathogens. Nature 502, 96–99. 10.1038/nature12503 23995682 PMC3825626

[B80] Nowicka B. Kruk J. (2010). Occurrence, biosynthesis and function of isoprenoid quinones. Biochim. Biophys. Acta 1797, 1587–1605. 10.1016/j.bbabio.2010.06.007 20599680

[B81] Olivares-Hernández R. Sunner H. Frisvad J. C. Olsson L. Nielsen J. Panagiotou G. (2010). Combining substrate specificity analysis with support vector classifiers reveals feruloyl esterase as a phylogenetically informative protein group. PLoS ONE 5:e12781. 10.1371/journal.pone.0012781 20877647 PMC2943907

[B82] Orth J. D. Conrad T. M. Na J. Lerman J. A. Nam H. Feist A. M.. (2011). A comprehensive genome-scale reconstruction of *Escherichia coli* metabolism–2011. Mol. Syst. Biol. 7, 535. 10.1038/msb.2011.65 21988831 PMC3261703

[B83] Orth J. D. Thiele I. Palsson B. O. (2010). What is flux balance analysis? Nat. Biotechnol. 28, 245–248. 10.1038/nbt.1614 20212490 PMC3108565

[B84] Overbeek R. Begley T. Butler R. M. Choudhuri J. V. Chuang H. Y. Cohoon M.. (2005). The subsystems approach to genome annotation and its use in the project to annotate 1000 genomes. Nucleic Acids Res. 33, 5691–5702. 10.1093/nar/gki866 16214803 PMC1251668

[B85] Palsson B. (2006). Systems Biology: Properties of Reconstructed Networks. Cambridge: Cambridge University Press.

[B86] Qin J. Li R. Raes J. Arumugam M. Burgdorf K. S. Manichanh C.. (2010). A human gut microbial gene catalogue established by metagenomic sequencing. Nature 464, 59–65. 10.1038/nature08821 20203603 PMC3779803

[B87] Ramakrishna B. S. (2013). Role of the gut microbiota in human nutrition and metabolism. J. Gastroenterol. Hepatol. 28(Suppl. 4), 9–17. 10.1111/jgh.12294 24251697

[B88] Ramotar K. Conly J. M. Chubb H. Louie T. J. (1984). Production of menaquinones by intestinal anaerobes. J. Infect. Dis. 150, 213–218. 10.1093/infdis/150.2.213 6470528

[B89] Ratnikov B. I. Cieplak P. Gramatikoff K. Pierce J. Eroshkin A. Igarashi Y.. (2014). Basis for substrate recognition and distinction by matrix metalloproteinases. Proc. Natl. Acad. Sci. U.S.A. 111, E4148–E4155. 10.1073/pnas.1406134111 25246591 PMC4210027

[B90] Ravcheev D. A. Thiele I. (2014). Systematic genomic analysis reveals the complementary aerobic and anaerobic respiration capacities of the human gut microbiota. Front. Microbiol. 5:674. 10.3389/fmicb.2014.00674 25538694 PMC4257093

[B91] Reddy S. K. Rosengren A. Klaubauf S. Kulkarni T. Karlsson E. N. De Vries R. P.. (2013). Phylogenetic analysis and substrate specificity of GH2 beta-mannosidases from Aspergillus species. FEBS Lett. 587, 3444–3449. 10.1016/j.febslet.2013.08.029 24021641

[B92] Sahoo S. Franzson L. Jonsson J. J. Thiele I. (2012). A compendium of inborn errors of metabolism mapped onto the human metabolic network. Mol. Biosyst. 8, 2545–2558. 10.1039/c2mb25075f 22699794

[B93] Sahoo S. Haraldsdóttir H. S. Fleming R. M. Thiele I. (2015). Modeling the effects of commonly used drugs on human metabolism. FEBS J. 282, 297–317. 10.1111/febs.13128 25345908

[B94] Shearer M. J. Fu X. Booth S. L. (2012). Vitamin K nutrition, metabolism, and requirements: current concepts and future research. Adv. Nutr. 3, 182–195. 10.3945/an.111.001800 22516726 PMC3648719

[B95] Shearer M. J. Newman P. (2008). Metabolism and cell biology of vitamin K. Thromb. Haemost. 100, 530–547. 10.1160/th08-03-0147 18841274

[B96] Shearer M. J. Newman P. (2014). Recent trends in the metabolism and cell biology of vitamin K with special reference to vitamin K cycling and MK-4 biosynthesis. J. Lipid Res. 55, 345–362. 10.1194/jlr.R045559 24489112 PMC3934721

[B97] Simon C. Daniel R. (2011). Metagenomic analyses: past and future trends. Appl. Environ. Microbiol. 77, 1153–1161. 10.1128/AEM.02345-10 21169428 PMC3067235

[B98] Sonnenburg J. L. Chen C. T. Gordon J. I. (2006). Genomic and metabolic studies of the impact of probiotics on a model gut symbiont and host. PLoS Biol. 4:e413. 10.1371/journal.pbio.0040413 17132046 PMC1661682

[B99] Stams A. J. Plugge C. M. (2009). Electron transfer in syntrophic communities of anaerobic bacteria and archaea. Nat. Rev. Microbiol. 7, 568–577. 10.1038/nrmicro2166 19609258

[B100] Suttie J. W. (1995). The importance of menaquinones in human nutrition. Annu. Rev. Nutr. 15, 399–417. 10.1146/annurev.nu.15.070195.002151 8527227

[B101] Suzuki T. A. Worobey M. (2014). Geographical variation of human gut microbial composition. Biol. Lett. 10:20131037. 10.1098/rsbl.2013.1037 24522631 PMC3949373

[B102] Thiele I. Fleming R. M. Que R. Bordbar A. Diep D. Palsson B. O. (2012). Multiscale modeling of metabolism and macromolecular synthesis in *E. coli* and its application to the evolution of codon usage. PLoS ONE 7:e45635. 10.1371/journal.pone.0045635 23029152 PMC3461016

[B103] Thiele I. Heinken A. Fleming R. M. (2013a). A systems biology approach to studying the role of microbes in human health. Curr. Opin. Biotechnol. 24, 4–12. 10.1016/j.copbio.2012.10.001 23102866

[B104] Thiele I. Hyduke D. R. Steeb B. Fankam G. Allen D. K. Bazzani S.. (2011). A community effort towards a knowledge-base and mathematical model of the human pathogen *Salmonella Typhimurium* LT2. BMC Syst. Biol. 5:8. 10.1186/1752-0509-5-8 21244678 PMC3032673

[B105] Thiele I. Swainston N. Fleming R. M. Hoppe A. Sahoo S. Aurich M. K.. (2013b). A community-driven global reconstruction of human metabolism. Nat. Biotechnol. 31, 419–425. 10.1038/nbt.2488 23455439 PMC3856361

[B106] Thiele I. Vo T. D. Price N. D. Palsson B. O. (2005). Expanded metabolic reconstruction of *Helicobacter pylori* (iIT341 GSM/GPR): an *in silico* genome-scale characterization of single- and double-deletion mutants. J. Bacteriol. 187, 5818–5830. 10.1128/JB.187.16.5818-5830.2005 16077130 PMC1196094

[B107] Tyakht A. V. Kostryukova E. S. Popenko A. S. Belenikin M. S. Pavlenko A. V. Larin A. K.. (2013). Human gut microbiota community structures in urban and rural populations in Russia. Nat. Commun. 4, 2469. 10.1038/ncomms3469 24036685 PMC3778515

[B108] Van Winckel M. De Bruyne R. Van De Velde S. Van Biervliet S. (2009). Vitamin K, an update for the paediatrician. Eur. J. Pediatr. 168, 127–134. 10.1007/s00431-008-0856-1 18982351

[B109] Vogt S. L. Peña-Díaz J. Finlay B. B. (2015). Chemical communication in the gut: effects of microbiota-generated metabolites on gastrointestinal bacterial pathogens. Anaerobe 34, 106–115. 10.1016/j.anaerobe.2015.05.002 25958185

[B110] Walker A. W. Duncan S. H. Louis P. Flint H. J. (2014). Phylogeny, culturing, and metagenomics of the human gut microbiota. Trends Microbiol. 22, 267–274. 10.1016/j.tim.2014.03.001 24698744

[B111] Walther B. Karl J. P. Booth S. L. Boyaval P. (2013). Menaquinones, bacteria, and the food supply: the relevance of dairy and fermented food products to vitamin K requirements. Adv. Nutr. 4, 463–473. 10.3945/an.113.003855 23858094 PMC3941825

[B112] Widhalm J. R. Van Oostende C. Furt F. Basset G. J. (2009). A dedicated thioesterase of the Hotdog-fold family is required for the biosynthesis of the naphthoquinone ring of vitamin K1. Proc. Natl. Acad. Sci. U.S.A. 106, 5599–5603. 10.1073/pnas.0900738106 19321747 PMC2660889

[B113] Winter S. E. Thiennimitr P. Winter M. G. Butler B. P. Huseby D. L. Crawford R. W.. (2010). Gut inflammation provides a respiratory electron acceptor for *Salmonella*. Nature 467, 426–429. 10.1038/nature09415 20864996 PMC2946174

[B114] Wu G. D. Chen J. Hoffmann C. Bittinger K. Chen Y. Y. Keilbaugh S. A.. (2011). Linking long-term dietary patterns with gut microbial enterotypes. Science 334, 105–108. 10.1126/science.1208344 21885731 PMC3368382

[B115] Wyman J. B. Heaton K. W. Manning A. P. Wicks A. C. (1978). Variability of colonic function in healthy subjects. Gut 19, 146–150. 10.1136/gut.19.2.146 631630 PMC1411830

[B116] Xu J. Bjursell M. K. Himrod J. Deng S. Carmichael L. K. Chiang H. C.. (2003). A genomic view of the human-*Bacteroides thetaiotaomicron* symbiosis. Science 299, 2074–2076. 10.1126/science.1080029 12663928

[B117] Yatsunenko T. Rey F. E. Manary M. J. Trehan I. Dominguez-Bello M. G. Contreras M.. (2012). Human gut microbiome viewed across age and geography. Nature 486, 222–227. 10.1038/nature11053 22699611 PMC3376388

[B118] Zhi X. Y. Yao J. C. Tang S. K. Huang Y. Li H. W. Li W. J. (2014). The futalosine pathway played an important role in menaquinone biosynthesis during early prokaryote evolution. Genome Biol. Evol. 6, 149–160. 10.1093/gbe/evu007 24398376 PMC3914697

[B119] Zomorrodi A. R. Maranas C. D. (2012). OptCom: a multi-level optimization framework for the metabolic modeling and analysis of microbial communities. PLoS Comput. Biol. 8:e1002363. 10.1371/journal.pcbi.1002363 22319433 PMC3271020

