# Supplementary material for: Genomic Analysis of the Human Gut Microbiome Suggests Novel Enzymes Involved in Quinone Biosynthesis
Source: Front Microbiol. 2016 Feb 9;7:128. doi: 10.3389/fmicb.2016.00128 (PMC4746308; doi:10.3389/fmicb.2016.00128)
Supplement: Supplementary file 3 [file Table3.PDF]

Table S3. Membrane quinone-interacting subunits of respiratory reductases.

Reductase	Quinone-interacting subunit(s), *Organism*	Evidence
Cyo	CyoAB, *Escherichia coli*	Experimental (Yap et al., 2010)
Qox	QoxAB, *Bacillus subtilis*	Predicted (Bossis et al., 2014)
Cyd	CydA, *Escherichia coli*	Experimental (Matsumoto et al., 2006)
Nar	NarI, *Escherichia coli*	Experimental (Zhao et al., 2003)
Nap	NapGH, *Escherichia coli*	Experimental (Brondijk et al., 2004)
	NapC, *Escherichia coli*	Experimental (Brondijk et al., 2002)
Nrf	NrfD, *Escherichia coli*	Experimental (Hussain et al., 1994;Berks et al., 1995)
	NrfH, *Wolinella succinogenes*	Experimental (Simon et al., 2000)
Ttr	TtrC, *Salmonella typhimurium*	Predicted (Hensel et al., 1999)
Phs	PhsC, *Salmonella typhimurium*	Experimental (Stoffels et al., 2012)
Tsr	TsrF, *Burkholderiales bacterium* 1_1_47	Predicted: homolog of the *E. coli* NrfD protein (this study)
Dsr	DsrP, *Allochromatium vinosum*	Experimental (Grein et al., 2010)
Psr	PsrC, *Wolinella succinogenes*	Experimental (Dietrich and Klimmek, 2002)
Tor	TorC, *Escherichia coli*	Experimental (Wissenbach et al., 1992)
	TorY, *Escherichia coli*	Predicted (Gon et al., 2000)
Dms	DmsC, *Escherichia coli*	Experimental (Geijer and Weiner, 2004)
	DmsH, *Gordonibacter pamelaeae*	Predicted: homolog of the *E. coli* NrfD protein (this study)
Ynf	YnfH, *Salmonella typhimurium*	Predicted (Guymer et al., 2009)
Frd	FrdCD, *Escherichia coli*	Experimental (Iverson et al., 1999)
	FrdC, *Bacteroides fragilis*	Predicted (Baughn and Malamy, 2003)
	FrdC, *Corynebacterium glutamicum*	Experimental (Kurokawa and Sakamoto, 2005)
Ydh	YdhD, *Escherichia coli*	Predicted: homolog of the *E. coli* NrfD protein (this study)
Arx	ArxC, *Ectothiorhodospira* sp. PHS-1	Predicted (Zargar et al., 2012)

REFERENCES

Baughn, A.D., and Malamy, M.H. (2003). The essential role of fumarate reductase in haem-dependent growth stimulation of *Bacteroides fragilis*. *Microbiology* 149**,** 1551-1558.

Berks, B.C., Ferguson, S.J., Moir, J.W., and Richardson, D.J. (1995). Enzymes and associated electron transport systems that catalyse the respiratory reduction of nitrogen oxides and oxyanions. *Biochim Biophys Acta* 1232**,** 97-173.

Bossis, F., De Grassi, A., Palese, L.L., and Pierri, C.L. (2014). Prediction of high- and low-affinity quinol-analogue-binding sites in the aa3 and bo3 terminal oxidases from Bacillus subtilis and Escherichia coli1. *Biochem J* 461**,** 305-314.

Brondijk, T.H., Fiegen, D., Richardson, D.J., and Cole, J.A. (2002). Roles of NapF, NapG and NapH, subunits of the Escherichia coli periplasmic nitrate reductase, in ubiquinol oxidation. *Mol Microbiol* 44**,** 245-255.

Brondijk, T.H., Nilavongse, A., Filenko, N., Richardson, D.J., and Cole, J.A. (2004). NapGH components of the periplasmic nitrate reductase of *Escherichia coli* K-12: location, topology and physiological roles in quinol oxidation and redox balancing. *Biochem J* 379**,** 47-55.

Dietrich, W., and Klimmek, O. (2002). The function of methyl-menaquinone-6 and polysulfide reductase membrane anchor (PsrC) in polysulfide respiration of Wolinella succinogenes. *Eur J Biochem* 269**,** 1086-1095.

Geijer, P., and Weiner, J.H. (2004). Glutamate 87 is important for menaquinol binding in DmsC of the DMSO reductase (DmsABC) from *Escherichia coli*. *Biochim Biophys Acta* 1660**,** 66-74.

Gon, S., Patte, J.C., Mejean, V., and Iobbi-Nivol, C. (2000). The *torYZ* (*yecK-bisZ*) operon encodes a third respiratory trimethylamine N-oxide reductase in *Escherichia coli*. *Journal of Bacteriology* 182**,** 5779-5786.

Grein, F., Pereira, I.A., and Dahl, C. (2010). Biochemical characterization of individual components of the *Allochromatium vinosum* DsrMKJOP transmembrane complex aids understanding of complex function in vivo. *J Bacteriol* 192**,** 6369-6377.

Guymer, D., Maillard, J., and Sargent, F. (2009). A genetic analysis of in vivo selenate reduction by *Salmonella enterica* serovar *Typhimurium* LT2 and *Escherichia coli* K12. *Archives of Microbiology* 191**,** 519-528.

Hensel, M., Hinsley, A.P., Nikolaus, T., Sawers, G., and Berks, B.C. (1999). The genetic basis of tetrathionate respiration in *Salmonella typhimurium*. *Mol Microbiol* 32**,** 275-287.

Hussain, H., Grove, J., Griffiths, L., Busby, S., and Cole, J. (1994). A seven-gene operon essential for formate-dependent nitrite reduction to ammonia by enteric bacteria. *Mol Microbiol* 12**,** 153-163.

Iverson, T.M., Luna-Chavez, C., Cecchini, G., and Rees, D.C. (1999). Structure of the Escherichia coli fumarate reductase respiratory complex. *Science* 284**,** 1961-1966.

Kurokawa, T., and Sakamoto, J. (2005). Purification and characterization of succinate:menaquinone oxidoreductase from *Corynebacterium glutamicum*. *Arch Microbiol* 183**,** 317-324.

Matsumoto, Y., Murai, M., Fujita, D., Sakamoto, K., Miyoshi, H., Yoshida, M., and Mogi, T. (2006). Mass spectrometric analysis of the ubiquinol-binding site in cytochrome bd from Escherichia coli. *J Biol Chem* 281**,** 1905-1912.

Simon, J., Gross, R., Einsle, O., Kroneck, P.M., Kroger, A., and Klimmek, O. (2000). A NapC/NirT-type cytochrome c (NrfH) is the mediator between the quinone pool and the cytochrome c nitrite reductase of *Wolinella succinogenes*. *Mol Microbiol* 35**,** 686-696.

Stoffels, L., Krehenbrink, M., Berks, B.C., and Unden, G. (2012). Thiosulfate reduction in *Salmonella enterica* is driven by the proton motive force. *J Bacteriol* 194**,** 475-485.

Wissenbach, U., Ternes, D., and Unden, G. (1992). An *Escherichia coli* mutant containing only demethylmenaquinone, but no menaquinone: effects on fumarate, dimethylsulfoxide, trimethylamine N-oxide and nitrate respiration. *Arch Microbiol* 158**,** 68-73.

Yap, L.L., Lin, M.T., Ouyang, H., Samoilova, R.I., Dikanov, S.A., and Gennis, R.B. (2010). The quinone-binding sites of the cytochrome bo3 ubiquinol oxidase from Escherichia coli. *Biochim Biophys Acta* 1797**,** 1924-1932.

Zargar, K., Conrad, A., Bernick, D.L., Lowe, T.M., Stolc, V., Hoeft, S., Oremland, R.S., Stolz, J., and Saltikov, C.W. (2012). ArxA, a new clade of arsenite oxidase within the DMSO reductase family of molybdenum oxidoreductases. *Environ Microbiol* 14**,** 1635-1645.

Zhao, Z., Rothery, R.A., and Weiner, J.H. (2003). Transient kinetic studies of heme reduction in Escherichia coli nitrate reductase A (NarGHI) by menaquinol. *Biochemistry* 42**,** 5403-5413.
