# Supplementary material for: Genomic Analysis of the Human Gut Microbiome Suggests Novel Enzymes Involved in Quinone Biosynthesis
Source: Front Microbiol. 2016 Feb 9;7:128. doi: 10.3389/fmicb.2016.00128 (PMC4746308; doi:10.3389/fmicb.2016.00128)
Supplement: Supplementary file 4 [file Table4.PDF]

Table S4. Experimental data on quinone biosynthesis for the genera containing studied genomes.

Genus	Synthesized quinones (this research)	Number of the studied genomes	Reference(s)
Acinetobacter	UQ	1	(1)
Actinomyces	MK+DMK	1	(1, 2)
Alistipes	MK+DMK	1	(3)
Bacillus	MK+DMK	1	(1)
Bacteroides	MK+DMK	44	(2, 4)
Bifidobacterium	-	15	(2, 4)
Butyrivibrio	-	2	(2)
Campylobacter	MK+DMK	2	(1)
Citrobacter	UQ+MK+DMK	2	(5)
Clostridium	-	21	(1, 2)
Corynebacterium	MK+DMK	1	(1)
Desulfovibrio	MK+DMK	2	(1)
Enterobacter	UQ+MK+DMK	1	(1)
Enterococcus	DMK	5	(6)
Escherichia	UQ+MK+DMK	7	(1)
Eubacterium	-	8	(4)
Fusobacterium	MK+DMK	14	(2)
Gordonibacter	MK+DMK	1	(7)
Helicobacter	MK+DMK	5	(8)
Klebsiella	UQ+MK+DMK	1	(1)
Lactobacillus	MK / -	36	(1)
Leuconostoc	MK+DMK	1	(1)
Listeria	MK+DMK	1	(1)
Parabacteroides	MK+DMK	4	(9)
Pediococcus	-	2	(1)
Prevotella	MK+DMK	2	(10)
Proteus	UQ+MK+DMK	1	(1)
Providencia	UQ+MK+DMK	4	(11)
Ralstonia	UQ	1	(12)
Ruminococcus	-	9	(2)
Salmonella	UQ+MK+DMK	1	(1)
Streptococcus	-	4	(1)
Veillonella	MK+DMK	2	(2, 4)

REFERENCES

1. **Meganathan R.** 2001. Biosynthesis of menaquinone (vitamin K2) and ubiquinone (coenzyme Q): a perspective on enzymatic mechanisms. Vitam Horm **61:**173-218.
2. **Fernandez F, Collins MD.** 1987. Vitamin K composition of anaerobic gut bacteria. FEMS Microbiol Lett **41:**175-180.
3. **Nagai F, Morotomi M, Watanabe Y, Sakon H, Tanaka R.** 2010. *Alistipes indistinctus* sp. nov. and *Odoribacter laneus* sp. nov., common members of the human intestinal microbiota isolated from faeces. Int J Syst Evol Microbiol **60:**1296-1302.
4. **Ramotar K, Conly JM, Chubb H, Louie TJ.** 1984. Production of menaquinones by intestinal anaerobes. J Infect Dis **150:**213-218.
5. **Novotny C, Kapralek F.** 1979. Participation of quinone and cytochrome b in tetrathionate reductase respiratory chain of *Citrobacter freundii*. Biochem J **178:**237-240.
6. **Portela CA, Smart KF, Tumanov S, Cook GM, Villas-Boas SG.** 2014. Global metabolic response of *Enterococcus faecalis* to oxygen. J Bacteriol **196:**2012-2022.
7. **Selma MV, Tomas-Barberan FA, Beltran D, Garcia-Villalba R, Espin JC.** 2014. *Gordonibacter urolithinfaciens* sp. nov., a urolithin-producing bacterium isolated from the human gut. Int J Syst Evol Microbiol **64:**2346-2352.
8. **Arakawa C, Kuratsu M, Furihata K, Hiratsuka T, Itoh N, Seto H, Dairi T.** 2011. Diversity of the early step of the futalosine pathway. Antimicrob Agents Chemother **55:**913-916.
9. **Sakamoto M, Benno Y.** 2006. Reclassification of *Bacteroides distasonis*, *Bacteroides goldsteinii* and *Bacteroides merdae* as *Parabacteroides distasonis* gen. nov., comb. nov., *Parabacteroides goldsteinii* comb. nov. and *Parabacteroides merdae* comb. nov. Int J Syst Evol Microbiol **56:**1599-1605.
10. **Sakamoto M, Umeda M, Ishikawa I, Benno Y.** 2005. *Prevotella multisaccharivora*x sp. nov., isolated from human subgingival plaque. Int J Syst Evol Microbiol **55:**1839-1843.
11. **Macinga DR, Cook GM, Poole RK, Rather PN.** 1998. Identification and characterization of aarF, a locus required for production of ubiquinone in *Providencia stuarti*i and *Escherichia coli* and for expression of 2'-N-acetyltransferase in P. stuartii. J Bacteriol **180:**128-135.
12. **Radu V, Frielingsdorf S, Evans SD, Lenz O, Jeuken LJ.** 2014. Enhanced oxygen-tolerance of the full heterotrimeric membrane-bound [NiFe]-hydrogenase of *Ralstonia eutropha*. J Am Chem Soc **136:**8512-8515.
