# Supplementary material for: Genomic Analysis of the Human Gut Microbiome Suggests Novel Enzymes Involved in Quinone Biosynthesis
Source: Front Microbiol. 2016 Feb 9;7:128. doi: 10.3389/fmicb.2016.00128 (PMC4746308; doi:10.3389/fmicb.2016.00128)

Figure S1. Maximum-likelihood trees for the (A) MenI (PF03061) and (B) MenY (PF08282) protein domains. The domain structures of the proteins are labelled with different colours. SEED identifiers for the proteins are shown; for their sequences, see the file Sequences S1 in the Supplementary materials.

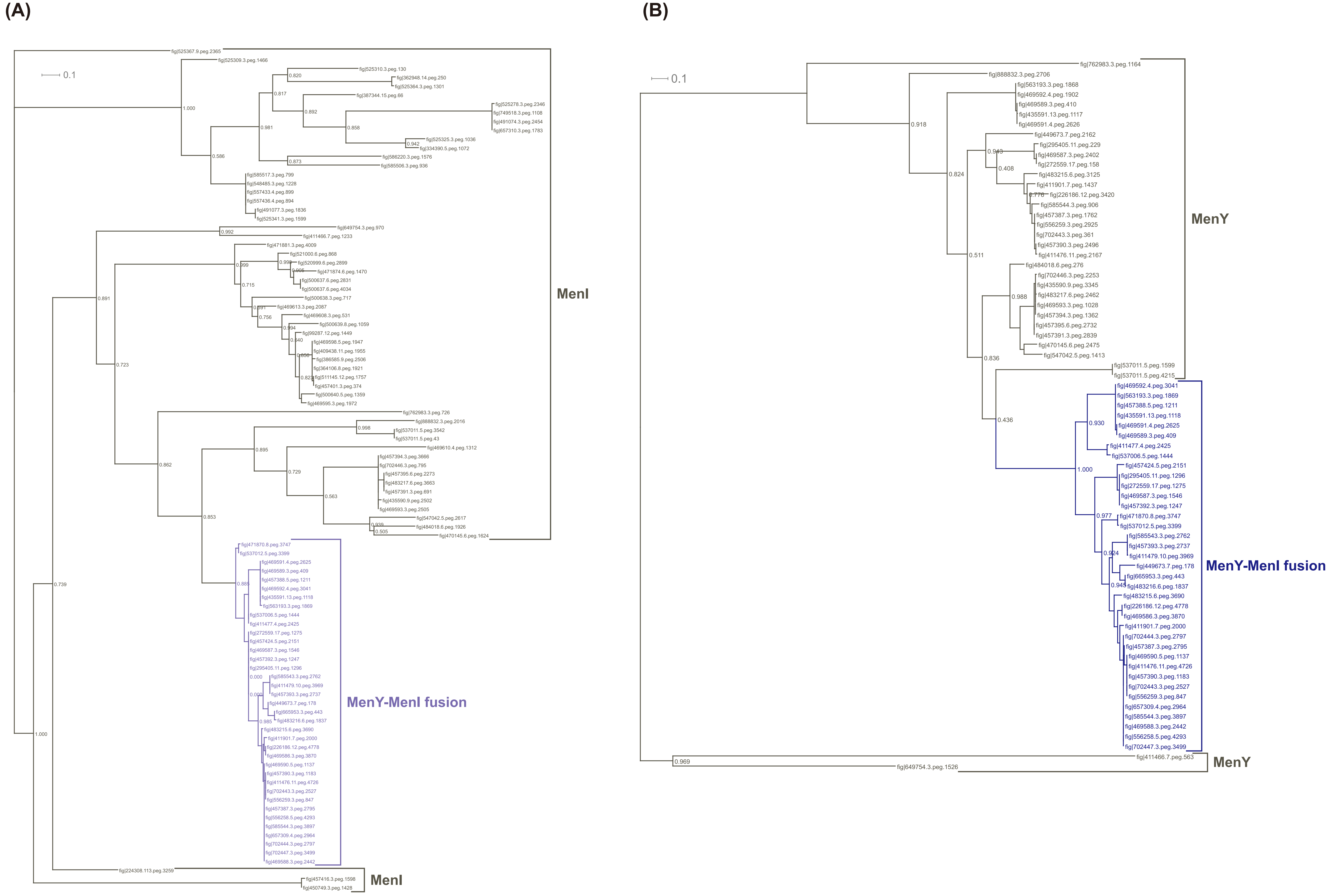

Supplement: Supplementary file 6 [file Image1.PDF]
