# Supplementary material for: Genomic Analysis of the Human Gut Microbiome Suggests Novel Enzymes Involved in Quinone Biosynthesis
Source: Front Microbiol. 2016 Feb 9;7:128. doi: 10.3389/fmicb.2016.00128 (PMC4746308; doi:10.3389/fmicb.2016.00128)

Figure S2. Maximum-likelihood tree for the MenG/UbiE proteins. The co-occurrence of the proteins with quinone biosynthesis pathways is shown by different colours. The SEED identifiers for proteins are shown; for their sequences, see the file Sequences S1 in the Supplementary materials.

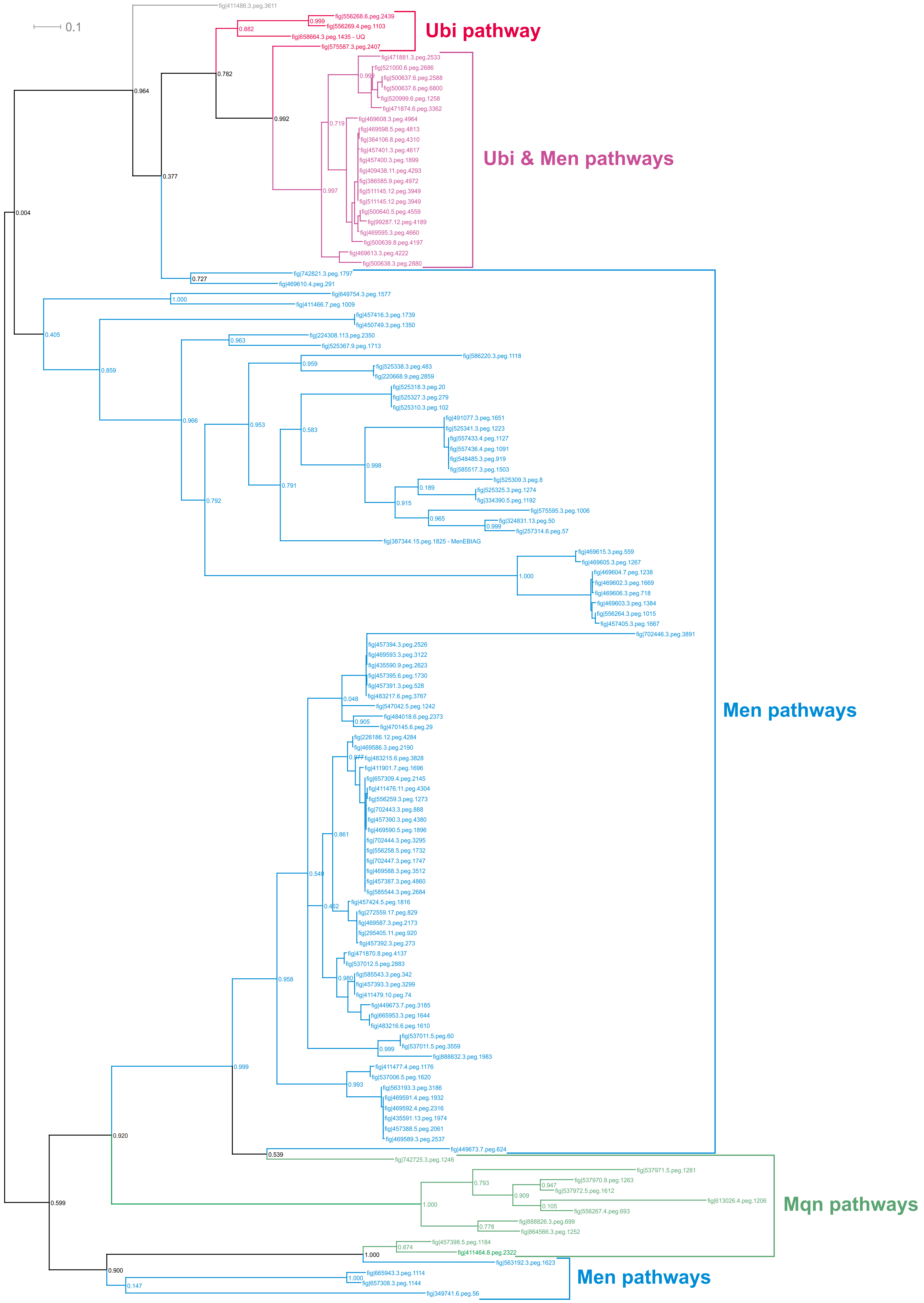

Supplement: Supplementary file 7 [file Image2.PDF]
