# Supplementary material for: Genomic Analysis of the Human Gut Microbiome Suggests Novel Enzymes Involved in Quinone Biosynthesis
Source: Front Microbiol. 2016 Feb 9;7:128. doi: 10.3389/fmicb.2016.00128 (PMC4746308; doi:10.3389/fmicb.2016.00128)
Supplement: Supplementary file 10 [file Image5.PDF]

Figure S5. Multiple alignments of the homologous Ubi and Mqn proteins, (A) UbiA and MqnP, (B) UbiD and MqnL, (C) UbiX and MqnM. Specificity-determining positions (SDP) are shown by light-blue background. SEED identifiers for the proteins are shown; for their sequences, see the file Sequences S1 in the Supplementary materials.

(A) UbiA and MqnP

UbiA

```
fig|364106.8.peg.4488   --------------------MEWSLTQNKLLAFHRLMRTDKPIGALLLLW-PTLWALWVATP-----GVPQLWILAVFVAGVWLMRAAGCVVNDYADRKFDGH
fig|386585.9.peg.5246   --------------------MEWSLTQNKLLAFHRLMRTDKPIGALLLLW-PTLWALWVATP-----GVPQLWILAVFVAGVWLMRAAGCVVNDYADRKFDGH
fig|409438.11.peg.4513  --------------------MEWSLTQNKLLAFHRLMRTDKPIGALLLLW-PTLWALWVATP-----GVPQLWILAVFVAGVWLMRAAGCVVNDYADRKFDGH
fig|457400.3.peg.314    --------------------MEWSLTQNKLLAFHRLMRTDKPIGALLLLW-PTLWALWVATP-----GVPQLWILAVFVAGVWLMRAAGCVVNDYADRKFDGH
fig|457401.3.peg.3312   --------------------MEWSLTQNKLLAFHRLMRTDKPIGALLLLW-PTLWALWVATP-----GVPQLWILAVFVAGVWLMRAAGCVVNDYADRKFDGH
fig|469595.3.peg.4401   --------------------MEWSLTQNKLLAFHRLMRTDKPIGALLLLW-PTLWALWVATP-----GMPQLWILAVFVAGVWLMRAAGCVVNDYADRKFDGH
fig|469598.5.peg.3324   --------------------MEWSLTQNKLLAFHRLMRTDKPIGALLLLW-PTLWALWVATP-----GVPQLWILAVFVAGVWLMRAAGCVVNDYADRKFDGH
fig|469608.3.peg.4217   --------------------MEWSLSQNKLLAFHRLMRTDKPIGALLLLW-PTLWALWVASP-----GVPPLWILAVFVAGVWLMRAAGCVVNDYADRKFDGH
fig|469613.3.peg.2740   ------------------------MTQTKWQAYCRLMRIDKPIGSLLLLW-PTYWALWLAGG-----QVPSLKLLVVFTLGVFFMRAAGCVVNDFADRRVDGH
fig|471874.6.peg.3746   --------------------MEGSMALSKWHAYSRLMRIDRPIGSLLLLW-PTYWALWIAAQ-----SIPSLHILIVFTAGVFLMRAAGCVINDFADRHFDGH
fig|471881.3.peg.3331   ------------------------MTQSKWQAYSRLMRIDKPIGALLLLW-PTYWALWIAAK-----GFPDWHILIVFTIGVFSMRAAGCVINDFADRKIDGS
fig|500637.6.peg.2465   --------------------MEGSMALSKWHAYSRLMRIDRPIGSLLLLW-PTYWALWIAAQ-----GTPSLHLLIVFTAGVFLMRAAGCVINDFADRNFDGY
fig|500637.6.peg.6923   --------------------MEGSMALSKWHAYSRLMRIDRPIGSLLLLW-PTYWALWIAAQ-----GTPSLHLLIVFTAGVFLMRAAGCVINDFADRNFDGY
fig|500638.3.peg.116    --------------------MEGVMTQGKWLAYCRLMRIDKPIGSLLLLW-PTYWALWLAGG-----GIPDVKLLLVFTGGVFFMRAAGCVINDFADRNFDGH
fig|500639.8.peg.2467   --------------------MEWSLTQDKLLAYHRLMRTDKPIGALLLLW-PTLWALWVATP-----GLPPLWILAVFVAGVWLMRAAGCVVNDYADRKFDGH
fig|500640.5.peg.4409   --------------------MEWSLTQSKLLAFHRLMRTDKPIGALLLLW-PTLWALWVATP-----GVPQLWILAVFVAGVWLMRAAGCVVNDYADRKFDGH
fig|511145.12.peg.4157  --------------------MEWSLTQNKLLAFHRLMRTDKPIGALLLLW-PTLWALWVATP-----GVPQLWILAVFVAGVWLMRAAGCVVNDYADRKFDGH
fig|520999.6.peg.1468   --------------------MEGSMALSKWHAYSRLMRIDRPIGSLLLLW-PTYWALWIAAQ-----GSPSLHILIVFTAGVFFMRAAGCVINDFADRHFDGH
fig|521000.6.peg.17     ------------------------MTLSKWHAYSRLMRIDRPIGSLLLLW-PTYWALWIAAQ-----GTPSLHLLIVFTAGVFFMRAAGCVINDFADRHFDGH
fig|556268.6.peg.2272   -------------------------MENRFLLYLRLIRMDKPVGTLLLLW-PTLCALWIAAE-----GKPSVTLVVIFCLGTVLMRSAGCAINDFADRDFDRH
fig|556269.4.peg.1008   ------------------------------MLYLRLVRLDKPIGSLLLLW-PTLCALWIASD-----GKPDWYLVLVFVLGTVLMRSAGCAINDFADRDFDRH
fig|575587.3.peg.376    -----------------METVQHITWRQRLEAYYYLCRFDKPIGTELVFW-PTMWALWIASK-----GIPDLKILLIMALGCLFMRAAGCAINDFADRKVDAH
fig|658664.3.peg.2752   ---------------MVISMQSSAAQPSRLMLYARLMRMDKPIGTLLLLW-PTLWALWMAAD-----GHPPLSLVVIFTVGTFLMRSAGCAVNDWADRDFDKH
fig|99287.12.peg.4454   --------------------MEWSLTQSKLLAFHRLMRTDKPIGALLLLW-PTLWALWVATP-----GMPQLWILAVFVAGVWLMRAAGCVVNDYADRKFDGH
```

MqnP

```
fig|100226.15.peg.4561  ---------------MTSASAALPQQPGRTKAFLRLVMIEHSVFALPFAYIAALTAMFLWDE---NIHWGRLLLVTVAMVGL---RTFAMAVNRIIDREIDAR
fig|411464.8.peg.2008   MKLPKFTLPRFSLPSCRPGALCGRFSVRGFVSFCRMIRIEHSIFALPYAW----AGSVMAAG-----GMPPWDKIILLTIAMVGVRSFAMGINRIFDLPFDRE
fig|457398.5.peg.2595   ----------------------MRTPFGQFADICRMIKIEHSVFALPYAW----AGAFLAAR-----GLPPLRSLIFLTIGMVAVRSFAMAFNRLVDLPFDRD
fig|500635.8.peg.1449   --------------------------MISIKAHINNVALHHTIFDLPFAF----MGAVLAAN-----GHPRLIDLFWVAMAITTGRAAAMAIDNLADLKYDKQ
fig|537970.9.peg.1538   -------------------------MLQKIKDFSELVMFQHSIFSMPFIF----IAMLTAAQ-----GWFGWKLLAFGVIASISARNFAMAFNRYADRKFDST
fig|537971.5.peg.1298   -------------------MSVLQNLSKQIKNFSELVAFEHTIFSSSFIL----IAMVVASMQQYGVAWCGWETFLLCVLALITARNFAMGFNRFKDRDIDAQ
fig|537972.5.peg.1533   -------------------------MFAKIKDFSELVMFQHSIFSMPFIF----IAMLTAAN-----GWFGWKLFAFGVIASISARNFAMAFNRYADRKFDST
fig|556267.4.peg.915    -----------------------MQFLQKVKDFRELVMFEHSIFSMPFIF----IAMVTAAQ-----GWFGVKLLIFGVIASVSARNFAMAFNRFADRKFDAT
fig|613026.4.peg.1062   -------------------------------------MFEHTIFSASFIV----IAMIVSSNAFYDSGFFGFKTLFLCAFALITARNFAMAFNRLCDRDIDST
fig|657316.3.peg.1682   --------------------------MSKLSAHINNIALHHSVFALPFAY----MGLFLAAK-----GIPTFHDFLWVTLAMIGARSSALAMDNLVDLKFDKQ
fig|742725.3.peg.1930   --------------------------MKTVARYASLVTFSHTVFAMPFALIAYFYALWSTDT------PFEWLLLVKVLLAMVFARNTAMGFNRYADRSIDAM
fig|864566.3.peg.1076   ----------------------MNTIWNKFKDILELVVFKHSIFALPFLF----SSMIVASKLVNDSAWFGFKALILGIICAVSARNFAMATNRLMDEDIDKD
fig|888826.3.peg.1565   ----------------------MSVLWVKIKDILELVVFKHSIFALPFLL----SAMIVASKLKNDSVWFGFSALILGIICAVSARNFAMATNRLMDEDIDKD
                                                                :.:            .                  .          *  .   :   *   *
```

(A) UbiA and MqnP, continuation

```
fig|364106.8.peg.4488     VKRTANRPLPSGAVTEKEARALFVVLVLISFLLVLTLNTMTILLSIAALALAWVYPFMKRYTHLPQVVLGAAFGWSIPMAFAAVS-ESVPLSCWLMFLANILW
fig|386585.9.peg.5246     VKRTANRPLPSGAVTEKEARALFVVLVLISFLLVLTLNTMTILLSIAALALAWVYPFMKRYTHLPQVVLGAAFGWSIPMAFAAVS-ESVPLSCWLMFLANILW
fig|409438.11.peg.4513    VKRTANRPLPSGAVTEKEARALFVVLVLISFLLVLTLNTMTILLSIAALALAWVYPFMKRYTHLPQVVLGAAFGWSIPMAFAAVS-ESVPLSCWLMFLANILW
fig|457400.3.peg.314      VKRTANRPLPSGAVTEKEARALFVVLVLISFLLVLTLNTMTILLSIAALALAWVYPFMKRYTHLPQVVLGAAFGWSIPMAFAAVS-ESVPLSCWLMFLANILW
fig|457401.3.peg.3312     VKRTANRPLPSGAVTEKEARALFVVLVLISFLLVLTLNTMTILLSIAALALAWVYPFMKRYTHLPQVVLGAAFGWSIPMAFAAVS-ESVPLSCWLMFLANILW
fig|469595.3.peg.4401     VKRTANRPLPSGAVTEKEARTLFVVLVLLAFLLVLTLNTMTILLSVAALALAWVYPFMKRYTHLPQVVLGAAFGWSIPMAFAAVS-ESVPLSCWLMFLANILW
fig|469598.5.peg.3324     VKRTANRPLPSGAVTEKEARALFVVLVLISFLLVLTLNTMTILLSIAALALAWVYPFMKRYTHLPQVVLGAAFGWSIPMAFAAVS-ESVPLSCWLMFLANILW
fig|469608.3.peg.4217     VKRTARRPLPSGDVTEKEARTLFIVLVLLSFLLVLTLNTMTILLSVAALALAWVYPFMKRYTHLPQVVLGAAFGWSIPMAFSAVS-ESLPLSCWLMFLANILW
fig|469613.3.peg.2740     VKRTAQRPLPSGAVTEKESKALFVILVLISFGLVLTLNAMTIWLSVAGLALAWVYPFMKRFTHLPQVVLGMAFGWSIPMAYAAVS-ESLPLSCWLLFLANICW
fig|471874.6.peg.3746     VERTKHRPLPSGDVTEKEAKILFASLVGLSFLLVLTLNSMTIWLSVAGLALAWIYPFVKRVSHLPQVVLGAAFGWSIPMGFSAVS-ESLPLVCWVLFLVNILW
fig|471881.3.peg.3331     VERTKNRPLPSGAVTEKESKILFIVLVLLSFALVLTLNTMTIWLSVAGLALAWFYPFVKRFSNLPQLILGMAFGWSIPMGFAAVS-ETLPLVCWLLFLVNIVW
fig|500637.6.peg.2465     VERTKHRPLPSGDVTEKEAKILFACLVGFSFLLVLTLNSMTIWLSVAGLALAWIYPFVKRVSNLPQVVLGAAFGWSIPMGFSAVS-ESLPLVCWLLFLVNIIW
fig|500637.6.peg.6923     VERTKHRPLPSGDVTEKEAKILFACLVGFSFLLVLTLNSMTIWLSVAGLALAWIYPFVKRVSNLPQVVLGAAFGWSIPMGFSAVS-ESLPLVCWLLFLVNIIW
fig|500638.3.peg.116      VKRTCQRPLPSGALSVREAKALFVLLVALSFALVLTLNALTIWLSVAALTLAWIYPFMKRFTHLPQVILGMAFGWSIPMAYAAVS-ESLPLSCWLLFAANICW
fig|500639.8.peg.2467     VKRTANRPLPSGQVTEKEARTLFVVLIALSFLLVLTLNTMTILLSVAALALAWVYPFMKRYTHLPQVVLGAAFGWSIPMAFAAVS-ESVPLSCWLMFLANILW
fig|500640.5.peg.4409     VKRTANRPLPSGAVTEKEARTLFVVLVLLAFLLVLTLNTMTILLSVAALALAWVYPFMKRYTHLPQVVLGAAFGWSIPMAFAAVS-ESVPLSCWLMFLANILW
fig|511145.12.peg.4157    VKRTANRPLPSGAVTEKEARALFVVLVLISFLLVLTLNTMTILLSIAALALAWVYPFMKRYTHLPQVVLGAAFGWSIPMAFAAVS-ESVPLSCWLMFLANILW
fig|520999.6.peg.1468     VERTKHRPLPSGDVTEKEAKILFAALVGLSFLLVLTLNTMTIWLSVAGLALAWIYPFVKRVSHLPQVVLGAAFGWSIPMGFSAVS-ESLPLVCWLLFLVNIIW
fig|521000.6.peg.17       VERTKHRPLPSGDVTEKEAKILFASLVGLSFLLVLTLNSMTIWLSVAGLALAWVYPFVKRVSNLPQVVLGAAFGWSIPMSFSAVG-ETLPVVCWLLFLVNIIW
fig|556268.6.peg.2272     VKRTAKRPLTSGKLKPSEAIWVAVVLSALSFLLVLPLNNLTIGLSVAAVLIAGTYPFFKRFFALPQAYLGIAFGFGIPMAFAAVQ-NQVPPIAWLLLLGNVFW
fig|556269.4.peg.1008     VKRTADRPLTSGKLRPLEAVWVACSLAFLSFLLVLSLNWLTVGLSVIALVVAATYPLFKRFFALPQAYLGIAFGFGIPMAFAAVQ-NNVPWIAWFLLLANIFW
fig|575587.3.peg.376      VERTKNRPLATGVIKAKEAVFVFLALVVLSASLLLFLPIEAFYWSFGALLLAFIYPFMKRYTHLPQVFLGAAFSWSIPMAYAAVG-QTPDLTCWLLYFGNLAW
fig|658664.3.peg.2752     VKRTKERPITAGLIAPWEALAVAAVLSLIAFTLILPLNALTKWMSVAAIVIAGTYPFFKRFFAIPQAYLGIAFGFGIPMAYAAVQ-DQVPVLAWLMLAGNVFW
fig|99287.12.peg.4454     VKRTVNRPLPSGAVTEKEARNLFVVLVLLAFLLVLTLNAMTILLSVAALALAWVYPFMKRYTHLPQVVLGAAFGWSIPMAFAAVS-ESLPLSCWLMFLANILW
fig|100226.15.peg.4561    NPRTAHRELVTGAMSVKHAWTGALIALVVFLGAAALLNPLCLALAPVAVIPMVVYPYGKRFTNFPQAILGLAQAMGPIGGWLAIT-GEWSWEAVILGLAVGVW
fig|411464.8.peg.2008     NPRTANRHLVTGEISVRQAWIFSLVMAAIFVLACAAINTVCLILSVPALLFAAVYSLTKRFSAICHFWLGATLGLAPLAGALAVNPEGLDMGPIMLFFAVTFW
fig|457398.5.peg.2595     NPRTMDRPLVTGDISVAQTWAFCGVMALIFIIACACLNSLCFWLAVPALLFAAAYSLLKRFTALCHFWLGATLGLAPLAGWISVNPASMDLSPVLLFLAVTFW
fig|500635.8.peg.1449     QPRMAYRAMVAGRISKREALAFIVICLVLMVLSVLQLQPICIYLLPVAAIPFIIYPFMKRVTGWVHMFLGLAIAMAPAGGWVGVS-GTITAPLIVLCTAVALW
fig|537970.9.peg.1538     NPRTKNRPSVDGRISPFAMLIFIFINALIFILMGWLINPLCFYLSFPILLILASYSLMKRLTSAAHLVLGLSLGLAPIAGVAAVS-GEIPLWSVWLCCGVLFW
fig|537971.5.peg.1298     NLRTNSRPSVDGRIKLSALIAFNIINALLFVAVSYLINTLAFYLSVPFLAILAFYSYTKRFSAVAHWVLGVCLGLAPIAGVIAVM-GEVPLWSVLLSLGVLFW
fig|537972.5.peg.1533     NPRTKNRPSVDGRISPFAMLVFILINAIVFIFMGWLINPLCFYLSVPILLILASYSLMKRFTSAAHLVLGLSLGLAPIAGVAAVS-GEIPLWSVWLCCGVLFW
fig|556267.4.peg.915      NPRTKNRPSVDGRISKGAMLLFIFVNALIFVAMGYVINPLCFYLSFPILIIILASYSLMKRFSSAAHLVLGLSLGLAPIAGVVAVS-GEIPLWSVYLCIGVLFW
fig|613026.4.peg.1062     NERTKNRPSVDGRLSLPALLLFCVINAVLFVFVSYLINDLAFKLSFPFLFILGVYSVMKRFSCLAHLVLGLSLGLAPIAGDIAVS-GAVHVWTIFLSLGVLFW
fig|657316.3.peg.1682     QNRMSKRPLVTGAIKPIEVIILIIVSLCIFLYSASQLAPICLKLTPICLFFLLFYPYTKRFTFLCHYFLGVALAMAPAGGYIAVT-GELPFGIILLSGGVCLW
fig|742725.3.peg.1930     NPRTAQRDIPAGRISARNALWFIIVNALLFAATAAWINFLAFCLSPLALTVLLGYSLTKRFTAWCHIVLGIALGIAPVGAYLAVT-GQFAVLPILLTGLVITW
fig|864566.3.peg.1076     NPRCANRPNVSGKIGRKSVWIFIILNAIIFILCSYFINTLAFYLSFPVLFVLAIYSAFKRFSSLAHLVLGFCLGLAPIAGSVIVM-GEIHIYSVILCLGVTFW
fig|888826.3.peg.1565     NPRCKDRPNISGRIGKMSVWGFILVNALVFIVCSYFINPLAFYLSFPVLFLLAFYSAFKRFSSLAHLVLGFCLGLAPVAGSIIVL-GEIHLFSVILCLGVTFW
                             *   *    * :              .       :                 *. **      :  **   . .   .   :           :
*
```

UbiA

MqnP

(A) UbiA and MqnP, continuation

UbiA

```
fig|364106.8.peg.4488     AVAYDTQYAMVDRDDDVKIGIKSTAILFGQYDKLIIG-IFQIGVLALMAIIGEL---NGLGWGY-YWSILV-AGALF--VYQQKLIANREREACFKAFMN-NN
fig|386585.9.peg.5246     AVAYDTQYAMVDRDDDVKIGIKSTAILFGQYDKLIIG-ILQIGVLALMAIIGEL---NGLGWGY-YWSILV-AGALF--VYQQKLIANREREACFKAFMN-NN
fig|409438.11.peg.4513    AVAYDTQYAMVDRDDDVKIGIKSTAILFGQYDKLIIG-ILQIGVLALMAIIGEL---NGLGWGY-YWSIVV-AGALF--VYQQKLIANREREACFKAFMN-NN
fig|457400.3.peg.314      AVAYDTQYAMVDRDDDVKIGIKSTAILFGQYDKLIIG-ILQIGVLALMAIIGEL---NGLGWGY-YWSILV-AGALF--VYQQKLIANREREACFKAFMN-NN
fig|457401.3.peg.3312     AVAYDTQYAMVDRDDDVKIGIKSTAILFGQYDKLIIG-ILQIGVLALMAIIGEL---NGLGWGY-YWSILV-AGALF--VYQQKLIANREREACFKAFMN-NN
fig|469595.3.peg.4401     AVAYDTQYAMVDRDDDLKIGIKSTAILFGQNDKLIIG-ILQVGVLVLMALVGWL---NGLGLGY-YWSLLV-AGALF--VYQQKLIVNRERDACFKAFMN-NN
fig|469598.5.peg.3324     AVAYDTQYAMVDRDDDVKIGIKSTAILFGQYDKLIIG-IFQIGVLALMAIIGEL---NGLGWGY-YWSILV-AGALF--VYQQKLIANREREACFKAFMN-NN
fig|469608.3.peg.4217     AVAYDTQYAMVDRDDDVKIGIKSTAILFGENDRLIIG-ILQVAVLALMGAVGWL---NGLGWEY-YWSLFV-AAGLF--GWQQKLIFNRERDNCFKAFMN-NN
fig|469613.3.peg.2740     TVAYDTQYAMVDRDDDLKIGIKSTAILFGRFDKLIIG-LLQLATLLLMVWIGYL---NQLSGAY-YWGILL-AGALF--IHQQKLIAQRERMPCFQAFLN-NN
fig|471874.6.peg.3746     SVIYDTQYAMVDRNDDLKIGVKSTAILFGQYDKLIIG-ILQIVMIVLLVLVGSL---ADLGAVY-YIALSL-SALLF--IYQQKLMVDRERAPCFKAFLN-NN
fig|471881.3.peg.3331     SVIYDTQYAMVDRNDDIKIGVKSTAILFGRYDKIIIG-ILQLVMLALLVGIGIL---LNLKGIY-YWSLLL-VTALF--IYQQKLIAERERAPCFQAFMN-NN
fig|500637.6.peg.2465     SVIYDTQYAMVDRNDDLKIGIKSTAILFGNYDKLIIG-LLQVVMVGLLVVIGFL---ANLGMVY-YAALVL-TSALF--VYQQQLMVNRERAPCFKAFMN-NN
fig|500637.6.peg.6923     SVIYDTQYAMVDRNDDLKIGIKSTAILFGNYDKLIIG-LLQVVMVGLLVVIGFL---ANLGMVY-YAALVL-TSALF--VYQQQLMVNRERAPCFKAFMN-NN
fig|500638.3.peg.116      TVAYDTQYAMVDRDDDLRIGIKSTAILFGRYDRLIVA-LLQLATLLLLLWVGRL---NPLGAVY-YWGLLL-VGGGF--VYQQRLIVKRARPACFQAFMS-NN
fig|500639.8.peg.2467     AVAYDTQYAMVDRDDDLKIGIKSTAILFGRHDKLIIG-IMQVAVVALMVAIGRL---NGLNWEF-YWSVLV-AGLLF--AYQQKLIAKREREACFKAFLN-NN
fig|500640.5.peg.4409     AVAYDTQYAMVDRDDDLKIGIKSTAILFGQNDKLIIG-ILQVGVLALMALVGWL---NGLGLGY-YWSLLV-AGALF--VYQQKLIANRERDACFKAFMN-NN
fig|511145.12.peg.4157    AVAYDTQYAMVDRDDDVKIGIKSTAILFGQYDKLIIG-ILQIGVLALMAIIGEL---NGLGWGY-YWSILV-AGALF--VYQQKLIANREREACFKAFMN-NN
fig|520999.6.peg.1468     SVIYDTQYAMVDRNDDLKIGVKSTAILFGQYDKRIIG-LLQVVMIGLLVVIGVL---ANLGLVY-YGSLVL-AAALF--VYQQQLMINRERAPCFKAFMN-NN
fig|521000.6.peg.17       SVIYDTQYAMVDRDDDLKIGVKSTAILFGQYDKLIIG-LLQLLMVGLLLVIGSL---AGLGTLY-YISLVL-VAGLF--IYQQQLMVNRERAPCFKAFMN-NN
fig|556268.6.peg.2272     ALAYDTEYAMVDRDDDLKIGICTSAITFGRFDVLAVM-FCYLIMMSIALFAGWQ---LQFGGWF-FAGW---CGALFCVVYHFRLIRNRDRDGCFQAFRH-NN
fig|556269.4.peg.1008     ALAYDTEYAMVDRDDDLKIGIFTSAITFGRYDVLAVM-ACYFMMLIIVLFAGIF---TGLGKWF-VMGWSL-AVLCA--AYHYRLIRNRDRNACFFAFRH-NN
fig|575587.3.peg.376      TVAYDTQYAITDREYDLKIGVKSTAILFGRYDIQIIG-VLQLVSLVLIGIALYI---ESLLVPFGIAGLIV-VAGDF--IYQTFKTLDRDPQLCFWAFRH-NR
fig|658664.3.peg.2752     AVAYDTAYAMVDRDDDLLIGIKTSAITFGRFDVAAIM-LCYAGFFGIMAWAGHV---MALSVAY-WIGFAA-AVALS--LWYFPMLQTRDRMKCF--------
fig|99287.12.peg.4454     AVAYDTQYAMVDRDDDIKIGIKSTAILFGRYDTLIIG-ILQLGVMALMALIGWL---NGLGWGY-YWAVLV-AGALF--VYQQKLIANREREACFKAFMN-NN
```

MqnP

```
fig|100226.15.peg.4561    IGGFDLIYACQDVETDRQVGVKSVPARFGVPAAIWGARACHTVTTALFAWYAVA---TDAGVFF-WLGLLI-VAGAF--LYEHTIVRPTDLSRLNRAFFSVNG
fig|411464.8.peg.2008     VGAFDIYYSFQDYDFDVAFRLCSVPSVYGPDTALAIAGFSHAVTSIFLFLTGIA---AGLAWP--WYVLWAGVSAIL--FWEHHLMRPQDLSRVNMVFFTLNG
fig|457398.5.peg.2595     VGAFDIYYAFQDLDFDLAFDLRSVPSVYGPDTALALAAFSHVMTAIFLLLTGLA---AHLSWP--WYAICLGIGVLL--LVEHRLMRPQDLRHVNTAFFTLNG
fig|500635.8.peg.1449     IGAFDAMYGAQDEAFDRSQGLHSLAVSYGAAGAFRIATAAHVICILCFLAVGFM---MHLSALY-FVGVLI-AAATL--CYQHRIVSPTDFSRVTQRYFMRNG
fig|537970.9.peg.1538     VAGFDLLYALQDIEHDKKEGLYSIPSVFGVQNTLWISRIFHLLTLIFWALYIIS---SDSGVFM-WIGLLI-STIAL--GYEQFLVS-RNFQNIPKAFFAVNG
fig|537971.5.peg.1298     VAGFDLLYSIQDMDFDKAKNLHSIPAYFGLRATLWISRLCHIVAVLFWGCFVYE---AHLGVVA-WIGVGL-GALML--SYEQYLVS-VHLKNIPKAFFVTNG
fig|537972.5.peg.1533     VAGFDLLYSLQDIEHDKKEGLHSVPRVFGIQNTLWISRLFHLLTLIFWGLFIMQ---SNRGFLM-WFGLII-AVFAL--AYEHFLVS-KNFHNIPKAFFVVNG
fig|556267.4.peg.915      VAGFDLLYALQDINHDKKEGLYSVPSVFGVQKTLWISRIFHALTLIFWALFIKE---AGLGMWM-WIGLVV-AVVAL--SVEQYLVS-KNFEHIPRAFFTING
fig|613026.4.peg.1062     VAGFDILYSLQDIEHDKRHNLYSIPARFGAENALQIARIFHIFSVFFWALFLRF---STTYHLA-IVGLIL-SACML--FYEH-LIVRKDFCNIPRAFFTTNG
fig|657316.3.peg.1682     IGSFDVIYGSQDRQFDLDHKLHSMATQFGVANAHKIAAFFHFISILCFIGAGIY---FDLSFIY-YIGVLI-AILTL--IYQHSLITPYDFSRLTQVYFMRNG
fig|742725.3.peg.1930     VSGFDVIYALQDAEFDRQHALHSIPARFGIRGAIGISILLHLITVYAIALIGSY---YGAGTFY-WIGAAI-FVALL--IFQHTIVTPRHLDRIGPTFGLLNG
fig|864566.3.peg.1076     TAGFDLLYSLQDMEYDKKVGLHSIPAKFGSKATLFISAFCHVLAVLFWLLFVWQVWGVALGNIA-LFGVII-SGIIL--AFEHKIVH-KNFAHIDKAFFTLNG
fig|888826.3.peg.1565     TAGFDLLYSLQDMEYDKKVGLHSVPAKFGASATLFISAFCHFLATLFWLLFVWV---APLGGLA-FFGVLI-CGAIL--MAEH-LIVRKNFAHIDRAFFTLNG
                                :*  *.  *   *   :  :  .   :*
```

(A) UbiA and MqnP, ending

Sequence ID	Alignment	Group
fig|364106.8.peg.4488	YVGLVLFLGLAMSYWHF----	UbiA
fig|386585.9.peg.5246	YVGLVLFLGLAMSYWHF----	UbiA
fig|409438.11.peg.4513	YVGLVLFLGLAMSYWHF----	UbiA
fig|457400.3.peg.314	YVGLVLFLGLAMSYWHF----	UbiA
fig|457401.3.peg.3312	YVGLVLFLGLAMSYWHF----	UbiA
fig|469595.3.peg.4401	YVGLVLFLGLAMSYWHF----	UbiA
fig|469598.5.peg.3324	YVGLVLFLGLAMSYWHF----	UbiA
fig|469608.3.peg.4217	YVGLVLFLGLAMSYL------	UbiA
fig|469613.3.peg.2740	YAGLVLFIGIVLSIWS-----	UbiA
fig|471874.6.peg.3746	YVGLILFIGIFLSYL------	UbiA
fig|471881.3.peg.3331	YVGFVLFAGILFSYF------	UbiA
fig|500637.6.peg.2465	YVGFILFIGMVISYL------	UbiA
fig|500637.6.peg.6923	YVGFILFIGMVISYL------	UbiA
fig|500638.3.peg.116	YVGLILFVAIWLAL-------	UbiA
fig|500639.8.peg.2467	YVGLVLFIGLAMSYWS-----	UbiA
fig|500640.5.peg.4409	YVGLVLFLGLAMSYWHL----	UbiA
fig|511145.12.peg.4157	YVGLVLFLGLAMSYWHF----	UbiA
fig|520999.6.peg.1468	YVGLILFFGMVVSYF------	UbiA
fig|521000.6.peg.17	FVGLILFIGIFISYF------	UbiA
fig|556268.6.peg.2272	WLGAFIFAGIALDYMLR----	UbiA
fig|556269.4.peg.1008	WLGAFIFLGIALDYALR----	UbiA
fig|575587.3.peg.376	WVGMIIFLGIFLQLVV-----	UbiA
fig|658664.3.peg.2752	-----LFPA------------	UbiA
fig|99287.12.peg.4454	YVGLVLFLGLAMSYWHF----	UbiA
fig|100226.15.peg.4561	FIGIALFVCALLDLLVRGLTV	MqnP
fig|411464.8.peg.2008	IISPLVLVGVILGVVL-----	MqnP
fig|457398.5.peg.2595	IISPVVLMGVILGIYI-----	MqnP
fig|500635.8.peg.1449	IVSVAIFACTWLSFYI-----	MqnP
fig|537970.9.peg.1538	YLGIVFFGFCVLDLIFRN---	MqnP
fig|537971.5.peg.1298	YLGIIFFVCILLDSVVG----	MqnP
fig|537972.5.peg.1533	YLGIVFFGFCLLDLIFTK---	MqnP
fig|556267.4.peg.915	YLGIAFLGFCIVDFMSR----	MqnP
fig|613026.4.peg.1062	YLGFVFLFFIILDSIVRF---	MqnP
fig|657316.3.peg.1682	IVSIVIFVFTLIDILS-----	MqnP
fig|742725.3.peg.1930	ITSVCFATCVIIDLYLRY---	MqnP
fig|864566.3.peg.1076	YLSIIFFIFIWVDLVWK----	MqnP
fig|888826.3.peg.1565	YLSVVFFIFVLVDLLWR----	MqnP

.

(B) UbiD and MqnL

```
fig|364106.8.peg.4318    MDAMKYNDLRDFLTLLEQQGELKRITLPVDPHLEITEIADRTLRAGG---PALLFENP---------------------------------------------
fig|386585.9.peg.4980    MDAMKYNDLRDFLTLLEQQGELKRITLPVDPHLEITEIADRTLRAGG---PALLFENP---------------------------------------------
fig|409438.11.peg.4301   MDAMKYNDLRDFLTLLEQQGELKRITLPVDPHLEITEIADRTLRAGG---PALLFENP---------------------------------------------
fig|457400.3.peg.1907    MDAMKYNDLRDFLTLLEQQGELKRITLPVDPHLEITEIADRTLRVGG---PALLFENP---------------------------------------------
fig|457401.3.peg.4625    MDAMKYNDLRDFLTLLEQQGELKRITLPVDPHLEITEIADRTLRAGG---PALLFENP---------------------------------------------
fig|469595.3.peg.4668    MDAMKYHDLRDFLTLLEQQGELKRISLEVDPHLEITEIADRTLRAGG---PALLFENP---------------------------------------------
fig|469598.5.peg.4821    MDAMKYNDLRDFLTLLEQQGELKRITLPVDPHLEITEIADRTLRAGG---PALLFENP---------------------------------------------
fig|469608.3.peg.4956    ---MKYHDLRDFLTLLEQQGELKRITLPVDPHLEITEIADRTLRAGG---PALLFENP---------------------------------------------
fig|469613.3.peg.4232    ---MKYRDLREFLSLLEQRGELKRISQPIDPYLEMTEIADRTLRAGG---PALLFENP---------------------------------------------
fig|471874.6.peg.3370    ---MKYRDLRDFIAQLEKQGELKRITMEVDPYLEMTEIADRTLRAGG---PALLFENP---------------------------------------------
fig|471881.3.peg.2519    -------------------------------------------------------------------------------------------------------
fig|471881.3.peg.2520    --MMKYRDLRDFLSLLEEKGELKRITYEIDPYLEMTEIADRTLRAGG---PALLFENP---------------------------------------------
fig|500637.6.peg.2596    ---MKYRDLRDFIAQLEKQGELKRITMEVDPYLEMTEIADRTLRAGG---PALLFENP---------------------------------------------
fig|500637.6.peg.6792    ---MKYRDLRDFIAQLEKQGELKRITMEVDPYLEMTEIADRTLRAGG---PALLFENP---------------------------------------------
fig|500638.3.peg.2890    -------------------------------------------------------------------------------------------------------
fig|500639.8.peg.4205    ---MKYHDLRDFLALLEKQGELKRITLPVDPCLEMTEIADRTLRAGG---PALLFENP---------------------------------------------
fig|500640.5.peg.4567    MDAMKYHDLRDFLTLLEQQGELKRITLEVDPHLEITEIADRTLRAGG---PALLFENP---------------------------------------------
fig|511145.12.peg.3957   MDAMKYNDLRDFLTLLEQQGELKRITLPVDPHLEITEIADRTLRAGG---PALLFENP---------------------------------------------
fig|520999.6.peg.1266    ---MKYRDLRDFIAQLEKQGELKRITMEVDPYLEMTEIADRTLRAGG---PALLFENP---------------------------------------------
fig|521000.6.peg.2694    ---MKYRDLRDFISQLEKQGELKRITMEVDPYLEMTEIADRTLRAGG---PALLFENP---------------------------------------------
fig|556268.6.peg.1962    ---MKYRDLRDFINQLAAENELKEIVVSVSPHLEMTEVSSRVLTQNG---PALLFRKP---------------------------------------------
fig|556269.4.peg.713     ---MKYRDLRDFIEQLGRDGELKEIVVSVSPDLEMTEVSSRVLHQGG---PALLFKNP---------------------------------------------
fig|658664.3.peg.2251    ---MQYRDLRDFLAQLERTGELRRVHRPVSPRLEMTEVCDRLLRAEG---PAVVFEQPVDGA-----------------------------------------
fig|99287.12.peg.4197    MDAMKYHDLRDFLTLLEQQGELKRITLPVDPHLEITEIADRTLRAGG---PALLFENP---------------------------------------------
```

UbiD

```
fig|411464.8.peg.1208    ---MGYRNLQECVADLEKVGQLRRIDVPVDPHLELAHIQRRAFRSKS---PALLFTRV---------------------------------------------
fig|457398.5.peg.2296    -------------------------------------MLPRAKRNGG--------------------------------------------------------
fig|500635.8.peg.1448    ---MAYKDLREFIEALESKGLLKRIKTEVDPELEITEITDRVSKMKGEKNVALLFENV---------------------------------------------
fig|537970.9.peg.988     --------MRQTLDLLKAHNEVRIISEPLDIHLEIPHLAYLEVKKPNS--KALLFTNPIDKQRD---------------------------------------
fig|537971.5.peg.547     ----MALDSRQLIKFLQEHDELTIIDTPLDIYLEIPQLAYIEVKKLDS--KALLFTRPICKKSG---------------------------------------
fig|537972.5.peg.1697    --------MRQTLDLLKAHNELKIITEPLDIELEIPHLAYLEVKKPDS--KALLFTNPTRGN-----------------------------------------
fig|556267.4.peg.1586    --------MRQTIDKLKANNALKVISEPLDVELEIPHLAYLEVKSKDS--KALLFVNPVDKARG---------------------------------------
fig|613026.4.peg.1432    --------MQDFIRFLESHNELKIIDTPLDIELEIPHLAYLEVKKKDS--KALLFTRPIHRKHKDSTLSGTSTECTQHNMQSSNCHAKQSEISQNLDSKPCHT
fig|657316.3.peg.1681    -------------------------------------------------------------------------------------------------------
fig|742725.3.peg.914     ----MYKTLSEYIAALERAGELVRINVPVDPVLEIAELTDREAKSPGGG-RALLFEN----------------------------------------------
fig|864566.3.peg.1472    --------MKEFIQILKENDLLRVIEEPVDVDLEIAHLAYIEAKKGEKG-KALLFKNPIDKKLN---------------------------------------
fig|888826.3.peg.55      --------MRRFIELLKQNELLKIIDEPLDVELEIPHIAYIEAKKFEEG-KALLFTQPIREK-----------------------------------------
```

MqnL

(B) UbiD and MqnL, continuation

UbiD

```
fig|364106.8.peg.4318    ---------------------------------------------------------------------KGYSMPVLCNLFGTPKRVAMGM----GQEDVSAL
fig|386585.9.peg.4980    ---------------------------------------------------------------------KGYSMPVLCNLFGTPKRVAMGM----GQEDVSAL
fig|409438.11.peg.4301   ---------------------------------------------------------------------KGYSMPVLCNLFGTPKRVAMGM----GQEDVSAL
fig|457400.3.peg.1907    ---------------------------------------------------------------------KGYSMPVLCNLFGTPKRVAMGM----GQEDVSAL
fig|457401.3.peg.4625    ---------------------------------------------------------------------KGYSMPVLCNLFGTPKRVAMGM----GQEDVSAL
fig|469595.3.peg.4668    ---------------------------------------------------------------------KGYSMPVLCNLFGTPKRVAMGM----GQEDVSAL
fig|469598.5.peg.4821    ---------------------------------------------------------------------KGYSMPVLCNLFGTPKRVAMGM----GQEDVSAL
fig|469608.3.peg.4956    ---------------------------------------------------------------------KGYSMPVLCNLFGTPRRVALGM----GQEDVSSL
fig|469613.3.peg.4232    ---------------------------------------------------------------------KGYDIPVLCNLFGTAKRVALGM----GQEDVSAL
fig|471874.6.peg.3370    ---------------------------------------------------------------------KGYDMPVLCNLFGTTKRVAMGM----GQEDIKAL
fig|471881.3.peg.2519    -------------------------------------------------------------------------------------------------------
fig|471881.3.peg.2520    ---------------------------------------------------------------------KGYDMPVLCNLFGTPERVAMGM----GQDDVKAL
fig|500637.6.peg.2596    ---------------------------------------------------------------------KGYKMPVLCNLFGTTKRVAMGM----GQEDIKAL
fig|500637.6.peg.6792    ---------------------------------------------------------------------KGYKMPVLCNLFGTTKRVAMGM----GQEDIKAL
fig|500638.3.peg.2890    -------------------------------------------------------------------------------------------------------
fig|500639.8.peg.4205    ---------------------------------------------------------------------KGYSMPVLCNLFGTPRRVAMGM----GQEDVTAL
fig|500640.5.peg.4567    ---------------------------------------------------------------------KGYSMPVLCNLFGTPKRVAMGM----GQEDVSAL
fig|511145.12.peg.3957   ---------------------------------------------------------------------KGYSMPVLCNLFGTPKRVAMGM----GQEDVSAL
fig|520999.6.peg.1266    ---------------------------------------------------------------------KGYNMPVLCNLFGTTKRVAMGM----GQEDIKAL
fig|521000.6.peg.2694    ---------------------------------------------------------------------KGYNMPVLCNLFGTTKRVAMGM----GQEDIKAL
fig|556268.6.peg.1962    ---------------------------------------------------------------------TGYTIPVLTNLFGNTRRIARAM----GGETVQDL
fig|556269.4.peg.713     ---------------------------------------------------------------------SGFSIPVLTNLFGTSRRIAKAM----GGETTGDL
fig|658664.3.peg.2251    ---------------------------------------------------------------------QKYDMPVLANLFGTTRRVALGM----GAESLDEL
fig|99287.12.peg.4197    ---------------------------------------------------------------------KGYAMPVLCNLFGTPKRVAMGM----GQDDVSAL
```

MqnL

```
fig|411464.8.peg.1208    ---------------------------------------------------------------------KGCSFPMLANLFGTKERLHYIF----RD------
fig|457398.5.peg.2296    -------------------------------------------------------------------------APVL--------------------------
fig|500635.8.peg.1448    ---------------------------------------------------------------------KGSKMPVLMNAFGSYERMAMAL----GVEKLDDV
fig|537970.9.peg.988     ---------------------------------------------------------------------INFEIPVLMNLFGSFSRVELLI----G--DTRQI
fig|537971.5.peg.547     ---------------------------------------------------------------------KEFDIPVLMNVFGSHKRLDLLI----N-KPIPKI
fig|537972.5.peg.1697    ---------------------------------------------------------------------TSFEIPVLMNLFGNFKRVELLI----G--NTQEI
fig|556267.4.peg.1586    ---------------------------------------------------------------------VQYETPVLMNLFGSFKLVELLI----G--DVEGQ
fig|613026.4.peg.1432    GPLGEVSNMESKKDISPFRNAQHDKKLHPTTQMTQDLTYTAHIESNLIESNFNSIDSSNQDSNTTTQNIESFHIPVLMNVFGSQKRLELIATHYNSNADFKSL
fig|657316.3.peg.1681    -------------------------------------------------------------------------MPVLMNAFGSMERLALAF----GVNDIEEI
fig|742725.3.peg.914     ---------------------------------------------------------------------TGTPFPVLTNMMGSDRRIAMAL----GVESVDLL
fig|864566.3.peg.1472    ---------------------------------------------------------------------KQYKFPVLMNTFCNKKALNLAF----G-RDYEEV
fig|888826.3.peg.55      ---------------------------------------------------------------------KAYKYPVLMNTFCNQKALNLAL----T-RDYEEV
```

(B) UbiD and MqnL, continuation

UbiD

```
fig|364106.8.peg.4318    -REVGKLLAFLKEPEPPKGFRDLFDKLPQFKQVLNMPTKRLR-GAPCQQKIVSGDDVD-LNRIPIMTCWPEDAAPLITWGLTVTRGPHK---ERQNLGIYRQQ
fig|386585.9.peg.4980    -REVGKLLAFLKEPEPPKGFRDLFDKLPQFKQVLNMPTKRLR-GAPCQQKIVSGDDVD-LNRIPIMTCWPEDAAPLITWGLTVTRGPHK---ERQNLGIYRQQ
fig|409438.11.peg.4301   -REVGKLLAFLKEPEPPKGFRDLFDKLPQFKQVLNMPTKRLR-GAPCQQKIVSGDDVD-LNRIPIMTCWPEDAAPLITWGLTVTRGPHK---ERQNLGIYRQQ
fig|457400.3.peg.1907    -REVGKLLAFLKEPEPPKGFRDLFDKLPQFKQVLNMPTKRLR-GAPCQQKIVSGDDVD-LNRIPIMTCWPEDAAPLITWGLTVTRGPHK---ERQNLGIYRQQ
fig|457401.3.peg.4625    -REVGKLLAFLKEPEPPKGFRDLFDKLPQFKQVLNMPTKRLR-GAPCQQKIVSGDDVD-LNRIPIMTCWPEDAAPLITWGLTVTRGPHK---ERQNLGIYRQQ
fig|469595.3.peg.4668    -REVGKLLAFLKEPEPPKGFRDLFDKLPQFKQVLNMPTKRLR-GAPCQQKIYSGDDVD-LNRIPIMTCWPEDAAPLITWGLTVTRGPHK---ERQNLGIYRQQ
fig|469598.5.peg.4821    -REVGKLLAFLKEPEPPKGFRDLFDKLPQFKQVLNMPTKRLR-GAPCQQKIVSGDDVD-LNRIPIMTCWPEDAAPLITWGLTVTRGPHK---ERQNLGIYRQQ
fig|469608.3.peg.4956    -REVGKLLAFLKEPEPPKGFRDLFDKLPQFKQVLNMPTKRLR-GAPCQQKIIQGDDVD-LNKIPIMTCWPEDAAPLITWGLTVTRGPHK---ERQNLGIYRQQ
fig|469613.3.peg.4232    -REVGKLLAFLKEPEPPKGFRDLFDKLPQFKQVLNMPTKRLN-SAPCQELVWQGDDVD-LGRIPVMHCWPEDAAPLISWGLTITRGPHK---ERQNLGIYRQQ
fig|471874.6.peg.3370    -QDVGKLLAFLKEPDPPKGFRDLFDKLPKFKQVLNMPAKRLS-SAPCQEQIWAGDDVD-LTKIPVMHCWPEDAAPLITWGLTVTRGPNK---ERQNLGIYRQQ
fig|471881.3.peg.2519    ---------------------------------------------------------------------------------------------------------
fig|471881.3.peg.2520    -HEVGKLLAFLKEPDPPKGFRDLFDKLPKFKQVLNMPTKRLS-KAPCQEVVLTGDDVD-LTKIPVMHCWPEDAAPLITWGLTVTRGPLK---ERQNLGIYRQQ
fig|500637.6.peg.2596    -HDVGKLLAFLKEPDPPKGFRDLMDKLPKFKQVLNMPAKRLN-SAPCQEQIWAGDDVD-LTKIPVMHCWPEDAAPLITWGLTVTKGPHK---ERQNLGIYRQQ
fig|500637.6.peg.6792    -HDVGKLLAFLKEPDPPKGFRDLMDKLPKFKQVLNMPAKRLN-SAPCQEQIWAGDDVD-LTKIPVMHCWPEDAAPLITWGLTVTKGPHK---ERQNLGIYRQQ
fig|500638.3.peg.2890    ---------------------------------------------------------------------------------------------------------
fig|500639.8.peg.4205    -REVGKLLAFLKEPEPPKGFRDLFDKLPQFKQVLNMPTKRLR-GAPCQQKVLEGDAVD-LTKIPIMQCWPEDAAPLITWGLTVTRGPHK---ERQNLGIYRQQ
fig|500640.5.peg.4567    -REVGKLLAFLKEPEPPKGFRDLFDKLPQFKQVLNMPTKRLR-GAPCQQKIYSGDDVD-LNRIPIMTCWPEDAAPLITWGLTVTRGPHK---ERQNLGIYRQQ
fig|511145.12.peg.3957   -REVGKLLAFLKEPEPPKGFRDLFDKLPQFKQVLNMPTKRLR-GAPCQQKIVSGDDVD-LNRIPIMTCWPEDAAPLITWGLTVTRGPHK---ERQNLGIYRQQ
fig|520999.6.peg.1266    -HDVGKLLAFLKEPDPPKGFRDLMDKLPKFKQVLNMPAKRLS-SAPCQEEIWAGDDVD-LTKIPVMHCWPEDAAPLITWGLTVTKGPHK---ERQNLGIYRQQ
fig|521000.6.peg.2694    -HDVGKLLAFLKEPDPPKGFRDLFDKLPKFKQVLNMPAKRLS-SAPCQEQIWAGEDVD-LTKIPVMHCWPEDAAPLITWGLTVTKGPHK---ERQNLGIYRQQ
fig|556268.6.peg.1962    -RRIGLFLASLKEPEQPGSLSELFGMGSMLKSVLDMRPKERK-DAPCREIVMENDEID-LGAFPVQTCWPEDAGPLITWGLVVTKGPGK---KRQNLGVYRQQ
fig|556269.4.peg.713     -RRIGQFLASLKEPRQPEKFSDLLGMGAMLKSLMDMRPRELE-NAPCQEIVLEGKDVD-LHSLPIQTCWPEDAAPLITWGLVVTRGPNK---KRQNLGIYRQQ
fig|658664.3.peg.2251    -RDVGRLLSALKEPEPPRGLREAGKLWTMAKAVWDMAPRKVS-SPACQEIVWEGNDVD-LSRIPVQTCWPGDAAPLVTWGLVITRGPHK---KRQNLGIYRQQ
fig|99287.12.peg.4197    -REVGKLLAFLKEPEPPKGFRDLFDKLPQFKQVLNMPTKRLR-GAPCQQKIASGDDVD-LTRLPVMTCWPDDAAPLITWGLTVTRGPHK---ERQNLGIYRQQ
```

MqnL

```
fig|411464.8.peg.1208    -SLAGVEAVLAAKADPAAALKHPLRSLRALPALPHMLPRRTS-KAPVLE-----NRCA-LSDLPRLVGWPMDGGPFITLPLVYSEDPARPGLDASNLGMYRVQ
fig|457398.5.peg.2296    -------------------------------------------ECNCN-----------LADLPRLTCWPGDGGPFITLPLVYSEDPCRPGLNTSNLGMYRVQ
fig|500635.8.peg.1448    ADELREMMKLPY--VSLQNKMSVVTMIPMIKRAINF-PKYVK-HAPCQEVVET-ENPN-LDEIPILKCWPDDGGPFVTLPLVFTKNPKT---GKRNVGMYRLQ
fig|537970.9.peg.988     ANDLAFLLKL----KPPKNFKEIMQTLPKLLSLRYLSPKVVKNRGLCQEVIKTGSEVD-LQSLPILKTWSEDGGAFITMGQCYTQSLDG---SVRNLGMYRLQ
fig|537971.5.peg.547     AHSLQNLLHL----SPPKGIKQIFHKLKELYALRSVFPKYIKKKGQCQEIIKT-QNLN-LFDLPILTTWERDGGAFITMGQVYTQNLNG---TQKNLGMYRLQ
fig|537972.5.peg.1697    AKEIAFMLKL----KPPKNFQEALKFLPRLLNLRHLSPKILNTRGLCQEVIKTNNEIN-LTSLPILKTWSDDGGAFITMGQCYTQSLDG---SVKNLGMYRLQ
fig|556267.4.peg.1586    ASEVAKLLKL----KPPRSFKQALALLPSLLNLRNLSPKILKGRGLCQEVIKQGSEVD-LEAIPVLKTWSDDGGRFITMGQCYTQSLDG---CVRNLGMYRLQ
fig|613026.4.peg.1432    -ESIANVMKNLLNLSMPKTLSEKLAKLKELWAMRHVFPKKYKNKPPCQERIYTKDSID-LFSLPILKTWEEDGGRFITMGQVYTRSLDG---KSNNIGMYRLQ
fig|657316.3.peg.1681    PNELREILRLPY--ISLQNKMDLIHIIPTAKRAINF-PKYVK-KAPCQEVVITDNPT--LDKFPILKCWPQDGGPFITLPLVFTRNPKT---GKRNVGMYRLQ
fig|742725.3.peg.914     TGRIEELFAALTSPRP--DLFDKLRMLPLLGRMSRWLPREVKGRGVCQQVVLQGDAAR-LDLLPVLKCWPQDGGRFVTLPLVHTLDPDT---GVRNVGMYRMQ
fig|864566.3.peg.1472    AEEISKLIKL----HIPTSFKAKMDFFMNLLSFKNIPPKRLKK----NKALYDYEILNSLEELPILKTWEDDAGKFITMGQVYTQNLDK---TQNNLGMYRLQ
fig|888826.3.peg.55      ADEISQLTKL----HIPTSLKAKFDFFTKLSKLRFAPPKRLQK----EKALYRYELLPSLKELPILKTWSLDAAPFITMGQIYTQNPDK---TQNNLGMYRLQ
```

(B) UbiD and MqnL, continuation

```
UbiD
fig|364106.8.peg.4318    L----IGKNKLIMRWLSHRGGALDYQEWCAAHPGERFPVSVALGADPATILGAVTPVPDTLSEYAFAGLLRGTKTEVVKCIS-----NDLEVPASAEIVLEGY
fig|386585.9.peg.4980    L----IGKNKLIMRWLSHRGGALDYQEWCAAHPGERFPISVALGADPATILGAVTPVPDTLSEYAFAGLLRGTKTEVVKCIS-----NDLEVPASAEIVLEGY
fig|409438.11.peg.4301   L----IGKNKLIMRWLSHRGGALDYQEWCAAHPGERFPVSVALGADPATILGAVTPVPDTLSEYAFAGLLRGTKTEVVKCIS-----NDLEVPASAEIVLEGY
fig|457400.3.peg.1907    L----IGKNKLIMRWLSHRGGALDYQEWCAAHPGERFPVSVALGADPATILGAVTPVPDTLSEYAFAGLLRGTKTEVVKCIS-----NDLEVPASAEIVLEGY
fig|457401.3.peg.4625    L----IGKNKLIMRWLSHRGGALDYQEWCAAHPGERFPVSVALGADPATILGAVTPVPDTLSEYAFAGLLRGTKTEVVKCIS-----NDLEVPASAEIVLEGY
fig|469595.3.peg.4668    L----IGKNKLIMRWLSHRGGALDYQEWCAAHPGERFPVSVALGADPATILGAVTPVPDTLSEYAFAGLLRGTKTEVVKCIS-----NDLEVPASAEIVLEGY
fig|469598.5.peg.4821    L----IGKNKLIMRWLSHRGGALDYQEWCAAHPGERFPVSVALGADPATILGAVTPVPDTLSEYAFAGLLRGTKTEVVKCIS-----NDLEVPASAEIVLEGY
fig|469608.3.peg.4956    L----IGKNKLIMRWLSHRGGALDFQEWCAAHPGERFPVSVALGADPATILGAVTPVPDTLSEYAFAGLLRGTKTEVVKCVS-----NDLEVPASAEIVLEGY
fig|469613.3.peg.4232    V----LGKNKVIMRWLSHRGGALDFQEWCQAHPGERFPVSVALGADPATILGAVTPVPDTLSEYAFAGLLRGNKTEVVKCIS-----NDLEVPASAEIVLEGY
fig|471874.6.peg.3370    V----LGKNKVIMRWLSHRGGALDFQEWCQQHPGEKFPVAVALGADPATILGAVTPVPDTLSEYAFAGLLRGNKTEVVKCIS-----NDLEVPAGAEIILEGY
fig|471881.3.peg.2519    -------------------------------------------------------------------------------------------------------
fig|471881.3.peg.2520    V----LGKNKVIMRWLSHRGGALDFQEWCQKHPGERFPVSVALGADPATILGAVTPVPDTLSEYAFAGLLRGNKSEVVKCLS-----NDLEVPASAEIILEGY
fig|500637.6.peg.2596    V----LGKNKVIMRWLSHRGGALDFQEWCQQHPGEKFPVAVALGADPATILAAVTPVPDTLSEYAFAGLLRGNKTEVVKCIS-----NDLEVPAGAEIILEGY
fig|500637.6.peg.6792    V----LGKNKVIMRWLSHRGGALDFQEWCQQHPGEKFPVAVALGADPATILAAVTPVPDTLSEYAFAGLLRGNKTEVVKCIS-----NDLEVPAGAEIILEGY
fig|500638.3.peg.2890    -------------------------------------------------------------------------------------------------------
fig|500639.8.peg.4205    R----IGKNKLIMRWLSHRGGALDFQEWCAAHPGERFPVSVALGADPATILGAVTPVPDTLSEYAFAGLLRGTKTEVVKCIS-----NDLEVPASAEIVLEGY
fig|500640.5.peg.4567    L----IGKNKLIMRWLSHRGGALDYQEWRAAHPGERFPVSVALGADPATILGAVTPVPDTLSEYAFAGLLRGTKTEVVKCIS-----NDLEVPASAEIVLEGY
fig|511145.12.peg.3957   L----IGKNKLIMRWLSHRGGALDYQEWCAAHPGERFPVSVALGADPATILGAVTPVPDTLSEYAFAGLLRGTKTEVVKCIS-----NDLEVPASAEIVLEGY
fig|520999.6.peg.1266    V----LGKNKVIMRWLSHRGGALDFQEWCQAHPGERFPVAVALGADPATILGAVTPVPDTLSEYAFAGLLRGNKTEVVKCIS-----NDLEVPAGAEIILEGY
fig|521000.6.peg.2694    V----LGKNKVIMRWLSHRGGALDFQEWCQAHPGEKFPVAVALGADPATILGAVTPVPDTLSEYAFAGLLRGNKTEVVKCIS-----NDLEVPAGAEIILEGY
fig|556268.6.peg.1962    V----IGRNKLIMRWLAHRGGAQDFREHKIVNKGKPFPVAVVIGADPATLLSAVTPIPDELSEYQFAGLLRGSRTELVRTLG-----SDLMVPANAEMVLEGH
fig|556269.4.peg.713     V----ISGNKVIMRWLAHRGGAQDFREHKIANKGKPFPVAVVIGADPATLLSAVTPIPDELSEYQFAGLLRGQRTELVKTKN-----SDLQVPARAEIVLEGH
fig|658664.3.peg.2251    V----ISRNQLIMRWLAHRGGALDFREHAIANPGQPFPIVVALGADPATILGAVTPVPDTLSEYQFAGLLRGSRTELATCLTPSLAQAQLQVPAGAEIILEGH
fig|99287.12.peg.4197    L----IGKNKLIMRWLSHRGGALDFQEWLAARPGERFPVSVALGADPATILGAVTPVPDTLSEYAFAGLLRGTKTEVVKCLS-----NDLEVPASAEIILEGY

MqnL
fig|411464.8.peg.1208    LAGNDYAADEVGLHYQIHRGIGVHHAEALKRG--QSLPVNIFVGGPPAFTVAAVMPLPEGLSELRFAGLLGGCRAAMHYSRR-----LPLPVLAEADFCISGH
fig|457398.5.peg.2296    LAGNAYAPDEAGLHYQIHRGIGAHHANAIARG--EELPVHVYVGGPPALSVAAVMPLPEGLSELRFAGLLGGRRVELARAPE-----LRLPVLAEADFCISGR
fig|500635.8.peg.1448    K----YDSKTTGMHWHIHKNGAENFRDMKAQG-GERIEAAVAIGTDPVVTYAATAPLPRDIDEMVFAGFLRHKSVEMVKCKT-----VDIEVPATAEIILEGY
fig|537970.9.peg.988     V----YDKNHLGLHWQIHKDSVGIFEEYKKAN--QKMPVSIAIGGNPLYTWCATAPLPYGIFELMLYGFIKKEKARLVKCVS-----NELCVPYDSDLVIEGF
fig|537971.5.peg.547     V----YDETHLGLHWQIHKDSQHFFHEYKKAH--KEMPVSIALGGDPLYIWCGQAPLPLGIFELMLYGFIREKKPVLCPCIT-----NPLFVPYDVDIVIEGW
fig|537972.5.peg.1697    V----YDRNHLGLHWQIHKDSVGIFEEYKKAK--QKMPVSIAIGGDPLYTWCATAPLPYGMFELMLYGFIKKRKAKMVKCVS-----NPLFVPYDSDIVIEGY
fig|556267.4.peg.1586    V----YDKNHLGLHWQIHKDSVHLLEEYRKAG--KKMPVSIAIGGDPLYTWCATAPLPYGMYELMLYGFIKKQRAKLVRCVS-----NDLCVPYDADFVIEGF
fig|613026.4.peg.1432    I----HSHNELLMHWQIHKDATHFFHEYKRAN--KLMPVSIAIGGDPLYIWCAQAPMPPKIFELMLYGLIRAKNPLLAECVS-----NELAVPHDCDIVIEGF
fig|657316.3.peg.1681    K----YDGQTTGMHWHLHKDGASNYRAYQEMG-KDKIEVAVAIGTDPVITYAATAPLPRDIDEMVFAGFLRKKSVEMVKCKT-----VDVEVPACSEIVLEGY
fig|742725.3.peg.914     L----FSPQTTGMHWHLHKTGERHYRAYQRLG--KRMPVTVCLGGDPAYTYAATAPMPDNMDEYLLAGFLRERPVELVKCVT-----NDLRVPADCDIVIEGY
fig|864566.3.peg.1472    M----SDKNELLIHWQIHKDGANFYHEYKNAG-FKKMPVSIAIGGDPLYIWCSQAPLPKGIFELLLYGFIKKTPAKITPC-E-----NGIFVPYDSDVVIEGY
fig|888826.3.peg.55      V----SGENELLMHWQIHKDGANFCREYKEAG-LSKMPVSIAIGGDPLYIWCAQAPLPKGIFELLLYGFIKRQNARLTPC-E-----NGIFVPYDSDFVIEGY
```

(B) UbiD and MqnL, continuation

UbiD

```
fig|364106.8.peg.4318    IEQGETAP-------EGPYGDHTGYYNEVDSFPVFTVTHITQREDAIYHSTYTGRPPDEPAVLGVALNEVFV-PILQKQFPEIVDFYLPPEGCSYRLAVVTIK
fig|386585.9.peg.4980    IEQGEMAP-------EGPYGDHTGYYNEVDSFPVFTVTHITQREDAIYHSTYTGRPPDEPAVLGVALNEVFV-PILQKQFPEIVDFYLPPEGCSYRLAVVTIK
fig|409438.11.peg.4301   IEQGETAP-------EGPYGDHTGYYNEVDSFPVFTVTHITQREDAIYHSTYTGRPPDEPAVLGVALNEVFV-PILQKQFPEIVDFYLPPEGCSYRLAVVTIK
fig|457400.3.peg.1907    IEQGETAP-------EGPYGDHTGYYNEVDSFPVFTVTHITQREDAIYHSTYTGRPPDEPAVLGVALNEVFV-PILQKQFPEIVDFYLPPEGCSYRLAVVTIK
fig|457401.3.peg.4625    IEQGETAP-------EGPYGDHTGYYNEVDSFPVFTVTHITQREDAIYHSTYTGRPPDEPAVLGVALNEVFV-PILQKQFPEIVDFYLPPEGCSYRLAVVTIK
fig|469595.3.peg.4668    IEPGELAP-------EGPYGDHTGYYNEVDSFPVFTVTHITQREDAIYHSTYTGRPPDEPAVLGVALNEVFV-PILQKQFPEIVDFYLPPEGCSYRLAVVTMK
fig|469598.5.peg.4821    IEQGETAP-------EGPYGDHTGYYNEVDSFPVFTVTHITQREDAIYHSTYTGRPPDEPAVLGVALNEVFV-PILQKQFPEIVDFYLPPEGCSYRLAVVTIK
fig|469608.3.peg.4956    IEAGEMAP-------EGPYGDHTGYYNEVDSFPVFTVTHITQREDAIYHSTYTGRPPDEPAVLGVALNEVFV-PILQKQFPEIVDFYLPPEGCSYRLAVVTMK
fig|469613.3.peg.4232    IEPGEMAP-------EGPYGDHTGYYNEIDNFPVFTVTHITQRRNPIYHSTYTGRPPDEPAVMGLALNEVFV-PILQKQFPEIVDFYLPPEGCSYRMAVVTMK
fig|471874.6.peg.3370    IEPGELAP-------EGPYGDHTGYYNEIDSFPVFTVTHVTQRRDAIYHSTYTGRPPDEPAVLGEALNEVLV-PILQKQFPEIVDFYLPPEGCSYRLAVVTMK
fig|471881.3.peg.2519    -----------------------------------------------------------------------------------------------------MK
fig|471881.3.peg.2520    IEPGELAP-------EGPYGDHTGYYNEIDSFPVFTITHLTRRKDAIYHSTYTGRPPDEPAVLGVALNEVLV-PILQKQFPEIVDFLFTP-------------
fig|500637.6.peg.2596    IEPGEMAP-------EGPYGDHTGYYNEIDDFPVFTVTHVTQRRDAIYHSTYTGRPPDEPAVLGEALNEVLV-PILQKQFPEIVDFYLPPEGCSYRLAVVTMK
fig|500637.6.peg.6792    IEPGEMAP-------EGPYGDHTGYYNEIDDFPVFTVTHVTQRRDAIYHSTYTGRPPDEPAVLGEALNEVLV-PILQKQFPEIVDFYLPPEGCSYRLAVVTMK
fig|500638.3.peg.2890    -------------------------------------------------------------------------------------------------MAVVTMK
fig|500639.8.peg.4205    IEQGELAP-------EGPYGDHTGYYNEVDNFPVFTVTHITQRDDPIYHSTYTGRPPDEPAVLGVALNEVFV-PILQKQFPEIVDFYLPPEGCSYRLAVVTIK
fig|500640.5.peg.4567    IEPGEMAP-------EGPYGDHTGYYNEVDSFPVFTVTHITQREDAIYHSTYTGRPPDEPAVLGVALNEVFV-PILQKQFPEIVDFYLPPEGCSYRLAVVTIK
fig|511145.12.peg.3957   IEQGETAP-------EGPYGDHTGYYNEVDSFPVFTVTHITQREDAIYHSTYTGRPPDEPAVLGVALNEVFV-PILQKQFPEIVDFYLPPEGCSYRLAVVTIK
fig|520999.6.peg.1266    IEPGEMAP-------EGPYGDHTGYYNEIDDFPVFTVTHVTQRRDAIYHSTYTGRPPDEPAVLGEALNEVLV-PILQKQFPEIVDFYLPPEGCSYRLAVVTMK
fig|521000.6.peg.2694    IEPGEMAP-------EGPYGDHTGYYNEIDSFPVFTVTHVTQRRDAIYHSTYTGRPPDEPAVLGEALNEVLV-PILQKQFPEIVDFYLPPEGCSYRLAVVTMK
fig|556268.6.peg.1962    IYPDGNHGSGYEHALEGPFGDHTGYYNEPDWFPVMTVERITMRKNPLYLSTYTGRPPDEPAMLGLALNEVFV-PLLQKQFSEIVDFYLPAETCSYRFALVKIR
fig|556269.4.peg.713     IYPDESHESGYEHAMEGPFGDHTGYYNERDSFPVLTIDRITMRKNPIYLSTYTGKPPDEPAMLGLALNEVFV-PLLQKQFSEIIDFYLPAETCSYRFAVVKIR
fig|658664.3.peg.2251    IQPDPTHPSGYQHALEGPFGDHTGYYNEQDWFPVFTVERITMRRDPIYHSTYTGKPPDEPAVLGVALNEVFV-PLLQKQFPEIADFYLPPEGCSYRMALVSMK
fig|99287.12.peg.4197    IEPGEMAP-------EGPYGDHTGYYNEVDSFPVFTVTHITQREDAIYHSTYTGRPPDEPAVLGVALNEVFV-PILQKQFPEIVDFYLPPEGCSYRLAVVTMK
```

MqnL

```
fig|411464.8.peg.1208    ILPH-LKP-------EGPFGDHVGYYSLKHDFPILQVEAVHHRTGAIWPYTAVGRPPQEDTVFGDFIHELTG-ALVPQVFQGVREVHAVDAAGVHPLLLALGS
fig|457398.5.peg.2296    LVPG-LKP-------EGPFGDHVGYYSLRHDFPVLKVDAVYHRRDAVWPFTAVGRPPQEDTVFGDFIHELTA-PLVPQVFQGVREVHAVDVAGVHPLLLALGS
fig|500635.8.peg.1448    VRTDEMRR-------EGPFGDHTGYYSLADDYPVFHITAITHRKDAIYSATVVGKPPMEDCFLAKATERIFL-PLLQQMQPEIIDINMPLEGVFHDCCIVSIK
fig|537970.9.peg.988     VDTNELRD-------EGRFGDHTGFYTPIEPYPVLEVSAITHKKNPIYLASVVGKPPLEDKYLGYPTERIFL-PLLQTTTPSLIDYFMPENGVFHNLILAKIE
fig|537971.5.peg.547     VDTTQMRD-------EGPFGDHTGFYTPIEPYPVLEVSAITMRKKPIFPATIVGKPPLEDKYMGYLTERVFL-PLLQTTAQGLIDYHMPENGIFHNLIFAKIK
fig|537972.5.peg.1697    VDTEVLRD-------EGRFGDHTGFYTPIEPYPVLEVSAITHKQNPIYLATVVGKPPLEDKYLGYPTERIFL-PLLQTTTPSLVDYYMPENGVFHNLILAKIK
fig|556267.4.peg.1586    VEPT-MRD-------EGRFGDHTGFYTPIEPYPVLEVYAITSKKNPVYLATVVGKPPLEDKYLGYPTERIFL-PLLQTTTPDLMEYYMPENGVFHNLILAKIK
fig|613026.4.peg.1432    VDTSRFAP-------EGKFGDHTGFYTPIEPYPIMQVSAITMKHNPVYLATVVGKPPLEDKYMGYMTERLFL-PLLQTSAHGLIDYSMPENGVFHNLILAKVK
fig|657316.3.peg.1681    VNIGETRR-------EGPFGDHTGYYSLADDYPVFHITCITHRKNPIYSATIVGKPPMEDCFLAKATERIFL-PLLQQTLPEIRDINFPLEGVFHDCVMVSIK
fig|742725.3.peg.914     VDPSEEKV------IEGPFGDHTGFYSLEDYYPLFHVTAITHRRGAVYPATIVGVPPQEDAYIAKATEKIFLVPIRAAMLPEVRDLWMPEAGVAHNIAVVDIR
fig|864566.3.peg.1472    VDLEEFKI-------EGPFGDHTGFYTPAELFPVMKVEKIYAKKDAIYQATVVGKPPLEDKIMGLGTERIFL-PLLQTSVPDLIDYNMPENGVFHNLILAKID
fig|888826.3.peg.55      VDLENFKI-------EGPFGDHTGFYTPPELFPVMKVDKIYAKKDAVYQATVVGKPPLEDRVMGLGTERIFL-PLLQTTTPNLLDYKMPENGVFHNLILTKID
```

(B) UbiD and MqnL, continuation

UbiD

```
fig|364106.8.peg.4318     KQYAGH-----AKRVMMGVWSFLR--QFMYTKFVIVC--DDDVNAR--DWNDVIWAITTRMDPARDTVLVENTPIDYLDFASPVSGLGSKMGLDATNKWPG--
fig|386585.9.peg.4980     KQYAGH-----AKRVMMGVWSFLR--QFMYTKFVIVC--DDDVNAR--DWNDVIWAITTRMDPARDTVLVENTPIDYLDFASPVSGLGSKMGLDATNKWPG--
fig|409438.11.peg.4301    KQYAGH-----AKRVMMGVWSFLR--QFMYTKFVIVC--DDDVNAR--DWNDVIWAITTRMDPARDTVLVENTPIDYLDFASPVSGLGSKMGLDATNKWPG--
fig|457400.3.peg.1907     KQYAGH-----AKRVMMGVWSFLR--QFMYTKFVIVC--DDDVNAR--DWNDVIWAITTRMDPARDTVLVENTPIDYLDFASPVSGLGSKMGLDATNKWPG--
fig|457401.3.peg.4625     KQYAGH-----AKRVMMGVWSFLR--QFMYTKFVIVC--DDDVNAR--DWNDVIWAITTRMDPARDTVLVENTPIDYLDFASPVSGLGSKMGLDATNKWPG--
fig|469595.3.peg.4668     KQYAGH-----AKRVMMGVWSFLR--QFMYTKFVIVC--DDDVNAR--DWNDVIWAITTRMDPARDTVLVENTPIDYLDFASPVSGLGSKMGLDATNKWPG--
fig|469598.5.peg.4821     KQYAGH-----AKRVMMGVWSFLR--QFMYTKFVIVC--DDDVNAR--DWNDVIWAITTRMDPARDTVLVENTPIDYLDFASPVSGLGSKMGLDATNKWPG--
fig|469608.3.peg.4956     KQYAGH-----AKRVMMGVWSFLR--QFMYTKFVIVC--DDDVNAR--DWNDVIWAITTRMDPARDTVLVENTPIDYLDFASPVSGLGSKMGLDATNKWPG--
fig|469613.3.peg.4232     KQYPGH-----AKRVMMGVWSFLR--QFMYTKFVIVC--DDDINAR--DWNDVIWAITTRMDPARDTVMIENTPIDYLDFASPVSGLGSKMGLDATNKWPG--
fig|471874.6.peg.3370     KQYAGH-----AKRVMMGVWSYLR--QFMYTKFVIVC--DDDINAR--DWKDVIWAITTRMDPARDTVMMENTPIDYLDFASPVSGLGSKMGLDATNKWPG--
fig|471881.3.peg.2519     KQYAGH-----AKRVMMGVWSYLR--QFMYTKFVIVC--DDDVNAR--DWKDVIWAITTRMDPARDTIMMENTPIDYLDFASPVSGLGSKMGLDATNKWPG--
fig|471881.3.peg.2520     -------------------------------------------------------------------------------------------------------
fig|500637.6.peg.2596     KQYAGH-----AKRVMMGVWSYLR--QFMYTKFVIVC--DDDINAR--DWKDVIWAITTRMDPARDTIMMENTPIDYLDFASPISGLGSKMGLDATNKWPG--
fig|500637.6.peg.6792     KQYAGH-----AKRVMMGVWSYLR--QFMYTKFVIVC--DDDINAR--DWKDVIWAITTRMDPARDTIMMENTPIDYLDFASPISGLGSKMGLDATNKWPG--
fig|500638.3.peg.2890     KQYPGH-----AKRVMMGVWSFLR--QFMYTKFVIVC--DDDINAR--DWQDVIWALTTRMDPARDTLLVENTPIDYLDFASPVSGLGSKMGLDATNKWPG--
fig|500639.8.peg.4205     KQYAGH-----AKRVMMGVWSFLR--QFMYTKFVIVC--DDDVNAR--DWNDVIWAITTRMDPARDTVLVENTPIDYLDFASPVSGLGSKMGLDATNKWPG--
fig|500640.5.peg.4567     KQYAGH-----AKRVMMGVWSFLR--QFMYTKFVIVC--DDDVNAR--DWNDVIWAITTRMDPARDTVLVGNTPIDYLDFASPVSGLGSKMGLDATNKWPG--
fig|511145.12.peg.3957    KQYAGH-----AKRVMMGVWSFLR--QFMYTKFVIVC--DDDVNAR--DWNDVIWAITTRMDPARDTVLVENTPIDYLDFASPVSGLGSKMGLDATNKWPG--
fig|520999.6.peg.1266     KQYAGH-----AKRVMMGVWSYLR--QFMYTKFVIVC--DDDINAR--DWKDVIWAITTRMDPARDTVMMENTPIDYLDFASPISGLGSKMGMDATNKWPG--
fig|521000.6.peg.2694     KQYAGH-----AKRVMMGVWSYLR--QFMYTKFVIVC--DDDINAR--DWKDVIWAITTRMDPARDTVMMENTPIDYLDFASPVSGLGSKMGLDATNKWPG--
fig|556268.6.peg.1962     KAYPGH-----ARRVMFGVWSYLR--QFLYTKFIIVV--DEDIDIR--NWEEVLWAISTRVDPVRDTVLVNNTPIDYLDFASPVSGLGGKMGIDATDKWQG--
fig|556269.4.peg.713      KAYPGH-----ARRVMFGVWSFLR--QFLYTKFIIVV--DEDIDIR--NWGEVFWAISTRVDPARDTILVENTPIDYLDFASPVSGLGSKMGIDATDKWDG--
fig|658664.3.peg.2251     KQYAGH-----AKRVMFGVWSFLR--QFMYTKFIVVV--DDDVNLR--DWKEVIWAITTRVDPARDTVMVENTPIDYLDFASPVSGLGSKMGIDATNKWPG--
fig|99287.12.peg.4197     KQYAGH-----AKRVMMGVWSFLR--QFMYTKFVIVC--DDDVNAR--DWNDVIWAITTRMDPARDTVLVENTPIDYLDFASPVSGLGSKMGLDATNKWPG--
```

MqnL

```
fig|411464.8.peg.1208     ERYTPYEAQRRPRELLTAALHMLGTTQTALAKYVLVAAHEDAPGLRARDVVAFFRHLLERTDFERDLHFITRSTTDTLDYTGFALNEGSKLIWASAGEKRR--
fig|457398.5.peg.2296     ERYTPYEARRRPRELLTAALHLLGATQTALAKYVLLAAHEDAPGLCARDVPAFLRHLLERTDFSRDLHFLTRSTSDTLDYTGCGLHEGSKLIWASAGEKRR--
fig|500635.8.peg.1448     KSYPMQ-----ARKVMHALWG-MG--QMMNVKMIIVV--DAHVDVQ--DLGEVAWRVFNNIDAKHDLEIVE-GPLDVLDHSSPMAKWGAKLGIDATKTWPE--
fig|537970.9.peg.988      ARFPSA-----AKQSMHSFWG-VG--QMSFVKHAIFV--GEDAPSL--ESKDIIPYILNRFSVKN--CLFSEGVCDALDHASPNFAEGGKLGVDCTGEEIEN-
fig|537971.5.peg.547      PEYPSH-----SKQIMHAFWG-VG--QMSFVKHAIFT--DENVGDLA-DYETFGTYILHRFSPQN--LLITEGICDALDHSSPDFAHGGKLGVEAITPVEK--
fig|537972.5.peg.1697     ARFPSQ-----AKQSMHSFWG-VG--QMSFVKHAIFV--GEDSPSL--HTSEIIPYILNRFSVKN--CLFSEGVCDALDHSSPNFAEGGKLGIDCTGNEVEN-
fig|556267.4.peg.1586     ARFPNA-----AKQSMHAFWG-VG--QMSFVKHAIFV--DENAPKL--QDLEIIPYLLNRFSTQH--CLISDGVCDALDHASPEFAKGGKLGVDCTQDYAKDL
fig|613026.4.peg.1432     TDYPAQ-----SLQMMHTFFG-IG--QMSFVKHALFV--SEEAPKLHKDYKILCDYILDRINTKK--LYSTTGNCDALDHACERFAISGKLGIDASGEILN--
fig|657316.3.peg.1681     KTYPQQ-----AKKVMHAIWG-MG--QMMFTKMIIVV--DEHVDVQ--KEKEVWWRVFNNIDAKHDIVMVE-GPLDALDHSSPMAKWGTKIGIDATKTWPE--
fig|742725.3.peg.914      TDYAGQ-----ALKAASSLWG-AG--QMMFNKFMVVT--ASGRPVR--DVAALAD-LLRRIRIPDD-LMFSRGPLDVLDHAAPETGLGGKLMFDATGVDPA--
fig|864566.3.peg.1472     AKYPAH-----AQQIMHAFWG-VG--QMSFVKHAIFV--DKNAPSLK-DYDALIPYMLDRFNTKK--ILISEGICDQLDHASPNSCFGGKAGLDACEEIQV--
fig|888826.3.peg.55       THYPAH-----AQQIMHALWG-VG--QMSFVKHALFV--DKDAPNLE-DYKSLIPYILNRFNVEK--IYISQGICDQLDHASPNACFGGKAALDATCNFNA--
```

(B) UbiD and MqnL, continuation

UbiD

```
fig|364106.8.peg.4318    -------------------------------------------------------------------------------------------------------
fig|386585.9.peg.4980    -------------------------------------------------------------------------------------------------------
fig|409438.11.peg.4301   -------------------------------------------------------------------------------------------------------
fig|457400.3.peg.1907    -------------------------------------------------------------------------------------------------------
fig|457401.3.peg.4625    -------------------------------------------------------------------------------------------------------
fig|469595.3.peg.4668    -------------------------------------------------------------------------------------------------------
fig|469598.5.peg.4821    -------------------------------------------------------------------------------------------------------
fig|469608.3.peg.4956    -------------------------------------------------------------------------------------------------------
fig|469613.3.peg.4232    -------------------------------------------------------------------------------------------------------
fig|471874.6.peg.3370    -------------------------------------------------------------------------------------------------------
fig|471881.3.peg.2519    -------------------------------------------------------------------------------------------------------
fig|471881.3.peg.2520    -------------------------------------------------------------------------------------------------------
fig|500637.6.peg.2596    -------------------------------------------------------------------------------------------------------
fig|500637.6.peg.6792    -------------------------------------------------------------------------------------------------------
fig|500638.3.peg.2890    -------------------------------------------------------------------------------------------------------
fig|500639.8.peg.4205    -------------------------------------------------------------------------------------------------------
fig|500640.5.peg.4567    -------------------------------------------------------------------------------------------------------
fig|511145.12.peg.3957   -------------------------------------------------------------------------------------------------------
fig|520999.6.peg.1266    -------------------------------------------------------------------------------------------------------
fig|521000.6.peg.2694    -------------------------------------------------------------------------------------------------------
fig|556268.6.peg.1962    -------------------------------------------------------------------------------------------------------
fig|556269.4.peg.713     -------------------------------------------------------------------------------------------------------
fig|658664.3.peg.2251    -------------------------------------------------------------------------------------------------------
fig|99287.12.peg.4197    -------------------------------------------------------------------------------------------------------
```

MqnL

```
fig|411464.8.peg.1208    ------ELALEVHDLPPLPEGFGDARCAGPGILVLRGPRHELGRNET-DPRMEELA---------ACLAHWPQRDAFPLVVVADDAAFCAADFDNFLWVAFSR
fig|457398.5.peg.2296    ------ELGLELRGAPDLPEGFSEPRVAGPGLLVVRGPAHSLERGEPDPRMEELA----------RRLEAWPEREAFPLVAVVDDAAFCAAGLDNFLWVVFTR
fig|500635.8.peg.1448    -------------------------------------------------------------------------------------------------------
fig|537970.9.peg.988     PQLEILENEILLKQMKDIFPKA---EILRQYFKETKNPITLLGVKKDSNESLQKYL---------KESLFQTLQKHIKILVLLDWEKNDLENLYMILWRVVNN
fig|537971.5.peg.547     PDFCALSDEVLLKRITAIFSQA---VGLKQYFIHTPNPICIISVDKKSNLLNAVKE---------KMQAFENLHNHLSIVIFVDSHKNKLDNPYMLTWRIVNN
fig|537972.5.peg.1697    PPLEILDNQDLLDNLSSIIPLS---KTLRQYFLDTKNPITLLGVQKDSHSLQKFL----------KKSAFANLQKHLRILILLDDSKNDLENLYMILWRVVNN
fig|556267.4.peg.1586    KDCEVLSDNALQEILANVLKEGGEVKRVRQIYAETKNPIALVAINKKDSIGKAVLDLGHKKLESKNEQKLELLQKHTRIIVVVDNAKNDLENLYMLLWRVVNN
fig|613026.4.peg.1432    HNFVEISESELLKAMKNIAN-----EIESLHIYKHKNPLIICGINKKDTPILE------------YATRFYETLRQYGAFFIFVDSDNILTNYYMLVWRVVNS
fig|657316.3.peg.1681    -------------------------------------------------------------------------------------------------------
fig|742725.3.peg.914     ---------APLETVQLPFPFELSDGIVRVDYALSESWRVLLLSAPRGVRPD-------------VFAFLEKNRVEGIKYVILLDEEVDTSRPGDVLWIASSD
fig|864566.3.peg.1472    EELEILEDEKLLELFKTKVEL----LNLKQFYKESKSPIVCILLDKK-EKIEQSFD---------KLLEF---KKHFRILVFLDAE-NKLENSYMLVWRVVNN
fig|888826.3.peg.55      KELEILSDEMLKSLFEKELEI----HALKQFYTECKSPIVCILYDKK-ESVKASFE---------KLLKY---KKHFRILIFLDKE-NHLENPYMLVWRVVNN
```

(B) UbiD and MqnL, ending

```
fig|364106.8.peg.4318    --------------------------ETQREWG--RPIKKDPDVVAHI----------DAIWDELAIFNNGKSA-----------------------------
fig|386585.9.peg.4980    --------------------------ETQREWG--RPIKKDPDVVAHI----------DAIWDELAIFNNGKSA-----------------------------
fig|409438.11.peg.4301   --------------------------ETQREWG--RPIKKDPDVVAHI----------DAIWDELAIFNNGKSA-----------------------------
fig|457400.3.peg.1907    --------------------------ETQREWG--RPIKKDPDVVAHI----------DAIWDELAIFNNGKSA-----------------------------
fig|457401.3.peg.4625    --------------------------ETQREWG--RPIKKDPDVVAHI----------DAIWDELAIFNNGKSA-----------------------------
fig|469595.3.peg.4668    --------------------------ETQREWG--RPIKKDPEVTARI----------DAIWDELAIFNDGKSA-----------------------------
fig|469598.5.peg.4821    --------------------------ETQREWG--RPIKKDPDVVAHI----------DAIWDELAIFNNGKSA-----------------------------
fig|469608.3.peg.4956    --------------------------ETQREWG--RPIKKDPQVTARI----------DAIWDELAIFK----------------------------------
fig|469613.3.peg.4232    --------------------------ETQREWG--RPIVMDEKVRTRI----------DAIWDELAILEDNHTDAMKSRKPD---------------------
fig|471874.6.peg.3370    --------------------------ETNREWG--RPIVMDEQVKSRI----------DDIWDQLNIFEK---------------------------------
fig|471881.3.peg.2519    --------------------------ETDREWG--RPIVMSDEVKQRV----------DTIWEQLDILK----------------------------------
fig|471881.3.peg.2520    -------------------------------------------------------------------------------------------------------
fig|500637.6.peg.2596    --------------------------ETSREWG--RPIVMTESVKSRV----------DDIWEQLNIFEK---------------------------------
fig|500637.6.peg.6792    --------------------------ETSREWG--RPIVMTESVKSRV----------DDIWEQLNIFEK---------------------------------
fig|500638.3.peg.2890    --------------------------ETQREWG--RPIVMDERVRERV----------DAIWNELNIFANDDEDAQ---------------------------
fig|500639.8.peg.4205    --------------------------ETDREWG--RPIEKDPAVTARI----------DAIWDELAIMSNGKTESDR--------------------------
fig|500640.5.peg.4567    --------------------------ETQREWG--RPIKKDPEVTARI----------DAIWDELAIFNDGKSA-----------------------------
fig|511145.12.peg.3957   --------------------------ETQREWG--RPIKKDPDVVAHI----------DAIWDELAIFNNGKSA-----------------------------
fig|520999.6.peg.1266    --------------------------ETNREWG--RPIVMTQSVKSRV----------DDIWEQLNIIEK---------------------------------
fig|521000.6.peg.2694    --------------------------ETDREWG--RPIVMTESVKSRI----------DDIWEQLNIFDKK--------------------------------
fig|556268.6.peg.1962    --------------------------ETDRTWG--KKISMSAEVKGKV----------DSIWKELDW------------------------------------
fig|556269.4.peg.713     --------------------------ETSRKWG--TKINMKDEVRQKV----------DSIWKDLGL------------------------------------
fig|658664.3.peg.2251    --------------------------ETTREWG--QPIVMDTAVKAKV----------DAMWDTLFQ------------------------------------
fig|99287.12.peg.4197    --------------------------ETQREWG--RPIVKDPEVTARI----------DAIWDELAIFK----------------------------------
UbiD
fig|411464.8.peg.1208    SDPAADVYGTGAV-------------VRARHWSCEGPLLLDARIKPFHAPALEEDPAVQRRVDALAAPGGPLHGLIE--------------------------
fig|457398.5.peg.2296    SDPATDSYG-------------AHAQTRAKHWSCAAPLVLDARLKPFHAPPLEEDPAVTRGVEALAAPGGPLHGYF---------------------------
fig|500635.8.peg.1448    -------------------------EGHSREWP--DEIAMSEDIKAKV----------DAKWKELGLE-----------------------------------
fig|537970.9.peg.988     IDSKRDIRIFGDIIIIDATDK-NIADGYKREWP--KETDCDVKILESLKQKGLLEDFDEEELKEFYRKFHIDKSYATR-------------------------
fig|537971.5.peg.547     IDAKRDILVTQNLVFVDASDK-GKLEGHLREWP--LDTNCNAEVIESLRQKGLLQGIDEAFLERFGILY----------------------------------
fig|537972.5.peg.1697    IDSKRDIRILGEIVVIDATDK-NADDGYHREWP--KETDCDSKTLESLAQKGLLSDFSQESLEEFYRKYHIDKSYSTLLDSNQSS------------------
fig|556267.4.peg.1586    IDAKRDVVILDSIVFIDATDK-DARDGHLREWP--KETDCSIEVLRNLEVKGLLKDYGD--LEKFYRAFHIDKSYNC--------------------------
fig|613026.4.peg.1432    IDVARDLKIIKECAFLDATAK-GKLEGYDREWP--KDTLCSKSILNNLKEQGLLDDIDESFYKQFGIL-----------------------------------
fig|657316.3.peg.1681    -------------------------EGHTREWP--DEINMSDDIKKLV----------DAKWQEFGLE-----------------------------------
fig|742725.3.peg.914     CDPSRDVTIRDGVVLFDCRSKAGGVNGFSRRWP--NVVASSPETIARV----------DGRWNEYGLGKFMLSPSLHYSRLQYGEGAAVPAGGPLPGKTTEDT
fig|864566.3.peg.1472    IDAKRDIFIKEERLGVDASAK-GEAEGYLRAWP--KQTDCTKSVIEDLILRNILEN-NPDLFNKFEIF-----------------------------------
fig|888826.3.peg.55      LDAQRDIFIKNDCIALNATAK-NEFEAYQREWP--KQTDCSKKVVENLILRNIIEE-DTKLFKQFEIFG----------------------------------
MqnL
```

(C) UbiX and MqnM

```
fig|364106.8.peg.2621    -------------MKRLIVGISGASGAIYGVRLLQVLRDVTD---I-ETHLVMSQAARQTLSLETD----FSLREV--QALADV--TH-DARDIAASISSGSF
fig|386585.9.peg.3335    -------------MKRLIVGISGASGAIYGVRLLQVLRDVTD---I-ETHLVMSQAARQTLSLETD----FSLREV--QALADV--TH-DARDIAASISSGSF
fig|409438.11.peg.2782   -------------MKRLIVGISGASGAIYGVRLLQVLRDVTD---I-ETHLVMSQAARQTLSLETD----FSLREV--QALADV--TH-DARDIAASISSGSF
fig|457401.3.peg.1564    -------------MKRLIVGISGASGAIYGVRLLQVLRDVTD---I-ETHLVMSQAARQTLSLETD----FSLREV--QALADV--TH-DARDIAASISSGSF
fig|469595.3.peg.738     -------------MKRLIIGISGASGAIYGVRLLQVLRDVTD---V-ETHLVMSAAARQTLALETD----LSVRDV--QALANV--NH-DTRDIAASISSGSF
fig|469598.5.peg.2906    -------------MKRLIVGISGASGAIYGVRLLQVLRDVTD---I-ETHLVMSQAARQTLSLETD----FSLREV--QALADV--TH-DARDIAASISSGSF
fig|469608.3.peg.955     -------------MKRLIIGISGASGAIYGVRMLQVLRDVPE---I-ETHLILSQAARQTLALETD----FSVRDV--QALADV--VH-DARDIAASISSGSF
fig|469613.3.peg.1007    -------------MKRLIIGISGASGVIYGVRMLEVLRGVPD---I-ETHLIMSNAARQTLALETD----LQLRDV--QSLADV--VH-DARDIAASISSGSF
fig|471874.6.peg.2272    -------------MKKLIIGLTGASGAIYGVRLLEVLKPVKN---V-ETHLVMSAAARQTLSLETH----YSVKEV--QQLADH--HY-DSRDIAAAISSGSF
fig|471881.3.peg.360     -------------MKKLIVGLTGASGAIYGIRLLEILKSVPS---V-ETHLVISQAARRTISLETD----YSLKDI--HALADV--IY-DDRDIGASISSGSF
fig|500637.6.peg.1676    -------------MKKLIIGLTGASGAIYGIRLLEVLKPVQD---V-ETHLVMSAAARQTLALETD----YSVKDV--QQFADH--VY-DSRDIAAAISSGSF
fig|500637.6.peg.5858    ------MREIGADMKKLIIGLTGASGAIYGIRLLEVLKPVQD---V-ETHLVMSAAARQTLALETD----YSVKDV--QQFADH--VY-DSRDIAAAISSGSF
fig|500638.3.peg.1553    -------------MKRLIIGISGASGVIYGIRLLEVLRSHPE---V-ETHLVMSQAARQTLALETD----RSLREV--LALADV--VH-DVRDIAAAISSGSF
fig|500639.8.peg.140     -------------MKRLIIGLSGASGAIYGVRLLQVLRDVPE---V-ETHLVMSQAARQTLSLETD----LSLRDV--QSLADV--VH-DTRDIAASISSGSF
fig|500640.5.peg.2455    -------------MKRLIIGISGASGAIYGVRLLQVLRDVTD---V-ETHLVMSAAARQTLALETD----LSVRDV--QALANV--NH-DTRDIAASISSGSF
fig|511145.12.peg.2406   -------------MKRLIVGISGASGAIYGVRLLQVLRDVTD---I-ETHLVMSQAARQTLSLETD----FSLREV--QALADV--TH-DARDIAASISSGSF
fig|520999.6.peg.3060    -------------MKKLIIGLTGASGAIYGIRLLEVLKPVKD---V-ETHLVMSAAARQTLALETD----FSVKDV--QQLADH--VY-DSRDIAAAISSGSF
fig|521000.6.peg.2344    -------------MKKLIIGLTGASGAIYGVRLLEVLKPLKG---V-ETHLVMSAAARQTLALETT----YTVKDV--QQLADH--NY-DSRDIAAAISSGSF
fig|556268.6.peg.2345    ----------------MVVGMTGASGAIYGVRLMQILHRMDH---V-ETHLVISDAARLTLQHEMG----LSVADV--QLLADK--IY-DVNEMTGRIASGSF
fig|556269.4.peg.1022    ---------MIAQPKRLIVGITGASGAIYGVRLLRLLKNIDS---V-ETHLIISGAGELTLEHEQG----MKAREL--SLLADH--CY-DVNEVGAAVASGSF
fig|658664.3.peg.4432    MTQTANSTAQTPAVRRIIVAITGASGAIYGVRLLQILQSLRESAGV-ESHLLMSPAGLMNVQHELH----MAREEV--EALAHV--VH-NVRDIGATIASGSF
fig|99287.12.peg.2494    -------------MKRLIVGISGASGAIYGVRLLQILRDVDS---V-ETHLVMSQAARQTLALETH----FSLREV--QALADV--TH-DARDIAASISSGSY
fig|411464.8.peg.118     -------------MKRIIVGVSGASGMPLALCLLRHLAALPA---V-ESHLILSPGAHAVLEAECG----LAPSAL--HALADV--SH-DAACLGAAPASGSW
fig|457398.5.peg.457     -----MNGKENKSMRDILVGVSGASGMPLALCVLRALAAMPG---V-RTHCVVSEGAHAVLRAECG----AGPELL--TACADY--VY-EPDNLAAGPASGSW
fig|537970.9.peg.1226    -------------MKRIIIGISGASGAGLGLRFLKALP--KE---L-EKYCVISNGAKQVLFSEEGQNEYLEFEAI--QAKDNKIFML-EDSDMGACIASGSF
fig|537971.5.peg.1124    -----------MSIKKLVVAIGGASGVHLGVKFIECIP--KD---I-ELFVVVSEGAKAVALSEMGQEIESALDRA--RQSRDFR-IY-DEDELDSGISSGSF
fig|537972.5.peg.1595    -------------MKRVIVGISGASGAGLGLKFLESLP--KE---I-EKYCVISEGAKRVLSSEENIQETQKFEQI--KSRVSKVFLF-GDDELGACIASGSF
fig|613026.4.peg.1401    -----------MSYTPIVLGISGGSCIELALHFIKRFPKDRT---L---YVVPTQNAAMLCQAEKK----QNLRNLINAIRTDNLVIH-DS--MTSPLASGSF
fig|657316.3.peg.1506    -----MKTKGFKRKRRIIVGVSGASGAIYAYRLIQVLAD-SG---I-EVHFVASKAGLEVLEYECG----LTMVQL--TQIVHK--VY-DVNQIDSAIASGSF
fig|742725.3.peg.829     ----------MEKKRNIIVAVTGASGSLYARLLCRRLAEANG---VGRIALIVTRNGLAVSAYEDD----PAWMDD--PRFTR----Y-DNDDLFGAPASGSS
fig|864566.3.peg.584     --------------MKILLGISGSSSTHLGLKLLKNLE--NK---C-ELYCIITEGAKISFEAENK----KNLEKICQEQFQNI--CFLNDNNLSASVASGSF
fig|888826.3.peg.857     --------------MKVLLGITGSSSVHLGLKILEILE--QK---V-ELFCIITKGAKKSFEAENK----QNLSQIC-KKFNQC--VFLNDEDLSAGVSSGSF
                                         ::::.: *.*  .  .  .  :            :  :  .     *                                 :  .   :***
```

UbiX

MqnM

(C) UbiX and MqnM, continuation

```
fig|364106.8.peg.2621     ----QTLGMVILPCSIKTLSGIVHSYTDGLLTRAADVVLKERRPLVLCVRETPLHLGHLRLMTQAAEIGAVIMPPVPAFYHRPQSLDDVINQTVNRVLDQFAV
fig|386585.9.peg.3335     ----QTLGMVILPCSIKTLSGIVHSYTDGLLTRAADVVLKERRPLVLCVRETPLHLGHLRLMTQAAEIGAVIMPPVPAFYHRPQSLDDVINQTVNRVLDQFAI
fig|409438.11.peg.2782    ----QTLGMVILPCSIKTLSGIVHSYTDGLLTRAADVVLKERRPLVLCVRETPLHLGHLRLMTQAAEIGAVIMPPVPAFYHRPQSLDDVINQTVNRVLDQFAI
fig|457401.3.peg.1564     ----QTLGMVILPCSIKTLSGIVHSYTDGLLTRAADVVLKERRPLVLCVRETPLHLGHLRLMTQAAEIGAVIMPPVPAFYHRPQSLDDVINQTVNRVLDQFAI
fig|469595.3.peg.738      ----QTAGMVILPCSIKTLSGIVHSYTDGLLTRAADVVLKERRPLVLCVRETPLHLGHLRLMTQAAEIGAVIMPPVPAFYHRPQTLDDVINQTVNRVLDQFDI
fig|469598.5.peg.2906     ----QTLGMVILPCSIKTLSGIVHSYTDGLLTRAADVVLKERRPLVLCVRETPLHLGHLRLMTQAAEIGAVIMPPVPAFYHRPQSLDDVINQTVNRVLDQFAV
fig|469608.3.peg.955      ----KTAGMVILPCSMKTLSGIVHSYTDGLLTRAADVVLKERRPLVLCVRETPFHLGHLRLLVQAAELGAVIMPPVPAFYHRPQSLDDVINQTVNRVLDQFDI
fig|469613.3.peg.1007     ----KTAGMVILPCSMKTLSGIVHSYTDNLLTRAADVVLKERRRLVLCVRETPLHLGHLRMMTTAAELGAIVMPPVPAFYHRPQTLQDIVDQTVNRVIDQFDI
fig|471874.6.peg.2272     ----RTTGMVIMPCTMKTLSGIVHSYTDTLVTRAADVVLKEGKKLVLGVRETPLHLGHLKLLVQAAEMGAVIMPPMPAFYHQPKSIQDIVDQTVNRILDQFDI
fig|471881.3.peg.360      ----RVNGMVILPCSIKSLSGIVHSYTDTLVTRAADVVLKEGRKLVLCVRETPLHLGHLRLMTQASELGAVIMPPVPAFYFQPKTIDDIVNQTVNRVLDQFEI
fig|500637.6.peg.1676     ----RTVGMVIMPCTMKTLSGIVHSYTDTLVTRAADVVLKEGKKLVLGVRETPLHLGHLRLLVQAAEMGAVIMPPMPAFYHRPQAIQDIVDQTVNRVLDQFDI
fig|500637.6.peg.5858     ----RTVGMVIMPCTMKTLSGIVHSYTDTLVTRAADVVLKEGKKLVLGVRETPLHLGHLRLLVQAAEMGAVIMPPMPAFYHRPQAIQDIVDQTVNRVLDQFDI
fig|500638.3.peg.1553     ----KTAGMVILPCSIKTLSGIVHGYSDTLLIRAADVVLKERRPLVLCVRETPLHLGHLRMMTSAAELGAVIMPPVPAFYHRPQRIEEIIDQTVNRVLDQLDI
fig|500639.8.peg.140      ----KTAGMVILPCSIKTLSGIVNSYTDTLVTRAADVVLKERRPLVLCVRETPLHLGHLRLMTAAAELGAVIMPPVPAFYHRPKNLDDVINQTVNRVLDQFDI
fig|500640.5.peg.2455     ----QTAGMVILPCSIKTLSGIVHSYTDGLLTRAADVVLKERRPLVLCVRETPLHLGHLRLMTQAAEIGAVIMPPVPAFYHRPQTLDDVINQTVNRVLDQFDI
fig|511145.12.peg.2406    ----QTLGMVILPCSIKTLSGIVHSYTDGLLTRAADVVLKERRPLVLCVRETPLHLGHLRLMTQAAEIGAVIMPPVPAFYHRPQSLDDVINQTVNRVLDQFAI
fig|520999.6.peg.3060     ----RTAGMVIMPCTMKTLSGIVHSYTDTLVTRAADVVLKEGKKLVLGVRETPLHLGHLKLLVQAAEIGAVIMPPMPAFYHQPQSIQDIVDQTVNRVLDQFDI
fig|521000.6.peg.2344     ----RTAGMVIMPCTMKTLSGIVHSYTDTLVTRAADVVLKEGKKLVLGVRETPLHVGHLRLMVQAAEMGAVIMPPMPAFYHQPETIQDIIDQTVNRILDQFDI
fig|556268.6.peg.2345     ----QCAGMVVAPCSMKTLSAVASGFCDNLLTRAADVILKERRRLVLMVRETPFNLAHLRNMQSVTEMGGIIYPPVPAFYCRPLTIEEMVDQTVRRVLELFD-
fig|556269.4.peg.1022     ----KNAGMIVAPCSMKTLSAIAHGYCDNLITRAADVVLKERRRLVLMVRETPLNLAHIRNMEAVTEMGGIIYPPLPAFYCHPLSIDEMVDQTLRRVLDLFD-
fig|658664.3.peg.4432     ----AAEAMVVAPCSMKTLAAVAHGLSDNLIARAADVTLKERRRLILMVRETPLNLAHLRNMTAVTEMGGIVFPPVPGFYQRPKTIDDLVDHTVGRVLDLLG-
fig|99287.12.peg.2494     ----PTAGMVILPCSIKTLSGIVHSYTDGLLTRAADVILKERRPLVLCVRETPLHIGHLRLMTQAAEIGAVIMPPVPAFYHLPQTLDDVINQTVNRVLDQFDI
fig|411464.8.peg.118      ----QHDGMVVCPCSMSSLASIATGAGTNLLHRAADVTLKERRPLILVPRESPLSRIHLRNMIWASEAGAVIMPFMPAFYSGEDTLEGLMGQFCGRIFDQLG-
fig|457398.5.peg.457      WHGAEPAAMLVVPCSMGTLGALASGATRNLLQRAGDVALKEGLRLVLVTRESPLSAVHLRNMLTLREAGAVIMPFSPGFYLRPQSLEDLLLQTSGRIFDQLG-
fig|537970.9.peg.1226     ----VCEAMAVIPCSQNTLAKIACGISDTLITRAASVMIKEQRKLLLSPREMPLSPIVLENMLKLSRLGVIIAPPIMGYYAG-DNLKSIEEMLIGKWCDSLGI
fig|537971.5.peg.1124     ----GIDAMAIIPTSMNLLAKVAHGLCDDLISRCAAVMLKERRTLLLAPREMPFSPIALEQMSRLSNLGVIISPPNVGYYAKIRNLESMEYFFIGKWLDSLK-
fig|537972.5.peg.1595     ----VCEAMVVIPCSQNTLAKIACGISDTLITRCASVMIKEQRKLLLAPREIPFSQIALENMLKLSQIGVTIAPPVFGYYAG-KNLDEIENMLIGKWCDNLG-
fig|613026.4.peg.1401     ----YFETCIILPTSSNTLSSIANGLQSNLLTRVGAVCLKEKRKLILGVREMPLSAILLENMAKLANLGVYIAPPIAGYYGGICDLESLHDFLVGKYFDMLD-
fig|657316.3.peg.1506     ----PCESMVIVPCSMKTLGSLANGIAGNLLTRAADVTLKEGRKLVLVTRETPVHAIHLENMLKLSHAGARIVPACPGFYHRPQTIEELVDMLVGKICDTLN-
fig|742725.3.peg.829      ----SFDAMVVIPCSMGMAGRIAAGVSNDLASRAADVMLKERRRLILVPREAPLGTIHLRNLTTLSECGAVICPAAPSFYPHPRNIEDLCGTIVERVLTLLDV
fig|864566.3.peg.584      ----SIEKTIIAPCSISSLAKIHAGLADTLLMRAAAVALKERKSLILGVREMPFSTLILEHMHKLSQLGVIIAPPIIASYSHASNLEQMENFIIGKWLDLLG-
fig|888826.3.peg.857      ----GIDKTIIAPCSISTLAKIHAGFCDTLLTRACAVALKERKKLILAVRELPFSTLNLEHMAKLSLMGVIIAPPIYASYSKIKNLGDLENFIIGKWLDLLE-
                                    : * :    . :  .    *  *   * :**   *:*  ** *.    :. :      *  : *   . *     :  :      :    :
```

UbiX

MqnM

(C) UbiX and MqnM, ending

UbiX

```
fig|364106.8.peg.2621    TLPEDLFARWQGA-------------------------------
fig|386585.9.peg.3335    TLPEDLFARWQGA-------------------------------
fig|409438.11.peg.2782   TLPEDLFARWQGA-------------------------------
fig|457401.3.peg.1564    TLPEDLFARWQGA-------------------------------
fig|469595.3.peg.738     TLPQDLFTRWQGA-------------------------------
fig|469598.5.peg.2906    TLPEDLFARWQGA-------------------------------
fig|469608.3.peg.955     SLSQDLFTRWQGAQDCTK--------------------------
fig|469613.3.peg.1007    EISQDLFTRWDGGHKSQ---------------------------
fig|471874.6.peg.2272    ELDQDLFTRWQGGK------------------------------
fig|471881.3.peg.360     DLPDDLFQRWEGDKPAR---------------------------
fig|500637.6.peg.1676    QLEQDLFTRWQGAK------------------------------
fig|500637.6.peg.5858    QLEQDLFTRWQGAK------------------------------
fig|500638.3.peg.1553    TLPHELFPRWQGGHRTPDSAT-----------------------
fig|500639.8.peg.140     DLPEDLFTRWQGA-------------------------------
fig|500640.5.peg.2455    SLPQDLFTRWQGA-------------------------------
fig|511145.12.peg.2406   TLPEDLFARWQGA-------------------------------
fig|520999.6.peg.3060    QLDHDLFTRWQGAK------------------------------
fig|521000.6.peg.2344    ELEQDLFHRWQGAK------------------------------
fig|556268.6.peg.2345    -IVDDRIVRWEGTT------------------------------
fig|556269.4.peg.1022    -MADDQTSRWSGMV------------------------------
fig|658664.3.peg.4432    -LPQTLAPGWPGLRSTD---------------------------
fig|99287.12.peg.2494    PLPHDLFVRWQGA-------------------------------
```

MqnM

```
fig|411464.8.peg.118     -LSHQLCRRWQGPPDGTI--------------------------
fig|457398.5.peg.457     -LAHSL-GRWADA-------------------------------
fig|537970.9.peg.1226    FYK---YNRWGEKMIVLENMLKLSRLGVIIAPPIMGYYAGDNLK
fig|537971.5.peg.1124    -IQNSLYKRWKIDTD-----------------------------
fig|537972.5.peg.1595    -IPFE-YGRWQGCN------------------------------
fig|613026.4.peg.1401    -IPHDLFVRWDGNVRNQLIIP-----------------------
fig|657316.3.peg.1506    -VDNDLFERWTGPSEV----------------------------
fig|742725.3.peg.829     EVPHYEWGAFHPEPDHQ---------------------------
fig|864566.3.peg.584     -ISHNLYERWQNF-------------------------------
fig|888826.3.peg.857     -IDHNLYERWG---------------------------------
                                              :
```
